# Photodegradable polar-functionalized polyethylenes

**DOI:** 10.1093/nsr/nwad039

**Published:** 2023-02-16

**Authors:** Chaoqun Wang, Jian Xia, Yuxing Zhang, Xiaoqiang Hu, Zhongbao Jian

**Affiliations:** State Key Laboratory of Polymer Physics and Chemistry, Changchun Institute of Applied Chemistry, Chinese Academy of Sciences, Changchun 130022, China; School of Applied Chemistry and Engineering, University of Science and Technology of China, Hefei 230026, China; State Key Laboratory of Polymer Physics and Chemistry, Changchun Institute of Applied Chemistry, Chinese Academy of Sciences, Changchun 130022, China; State Key Laboratory of Polymer Physics and Chemistry, Changchun Institute of Applied Chemistry, Chinese Academy of Sciences, Changchun 130022, China; School of Applied Chemistry and Engineering, University of Science and Technology of China, Hefei 230026, China; State Key Laboratory of Polymer Physics and Chemistry, Changchun Institute of Applied Chemistry, Chinese Academy of Sciences, Changchun 130022, China; School of Applied Chemistry and Engineering, University of Science and Technology of China, Hefei 230026, China; State Key Laboratory of Polymer Physics and Chemistry, Changchun Institute of Applied Chemistry, Chinese Academy of Sciences, Changchun 130022, China; School of Applied Chemistry and Engineering, University of Science and Technology of China, Hefei 230026, China

**Keywords:** degradable plastic, homogeneous catalysis, C1 chemistry, non-alternating copolymerization, polyolefin

## Abstract

The degradation of plastics has attracted much attention from the global community. Polyethylenes (PEs), as the most abundant synthetic plastics, are most frequently studied. PE is non-degradable and non-polar because of the sole presence of the pure hydrocarbon components. Concurrent incorporation of both in-chain cleavable and functional groups into the PE chain is an effective pathway to overcome the non-degradable and non-polar issue; however, the method for achieving this pathway remains elusive. Here, we report a strictly non-alternating (>99%) terpolymerization of ethylene with CO and fundamental polar monomers via a coordination–insertion mechanism using late transition metal catalysts, which effectively prevents the formation of undesired chelates originating from both co-monomers under a low CO concentration. High-molecular-weight linear PEs with both in-chain isolated keto (>99%) and main-chain functional groups are prepared. The incorporation of key low-content isolated keto groups makes PEs photodegradable while retaining their desirable bulk material properties, and the introduction of polar functional groups considerably improves their surface properties.

## Teaser

Via the strictly nonalternating terpolymerization of ethylene, CO and polar monomers, high-molecular-weight high-density polar keto-polyethylenes are prepared, retaining the desirable properties of polyethylene materials and possessing both photodegradable ability and improved surface property.

## INTRODUCTION

Polyolefin plastics such as polyethylenes (PEs), which are the most important synthetic polymer used on the largest scale, are ubiquitous in modern society. Such plastics exhibit beneficial mechanical properties and incur low-cost, facile processing advantages [1,2]. However, because of the chemically inert structure of PE, it is extremely difficult for PE-based products to degrade after extensive use, thus causing environmental pollution [3–5]. In addition, the non-polar hydrocarbon nature of PEs makes PE plastics hydrophobic; thus, these plastics suffer from low surface properties and adhere to polar materials with difficulty, thereby limiting their broad-scale application [6–10].

**Figure 1.**
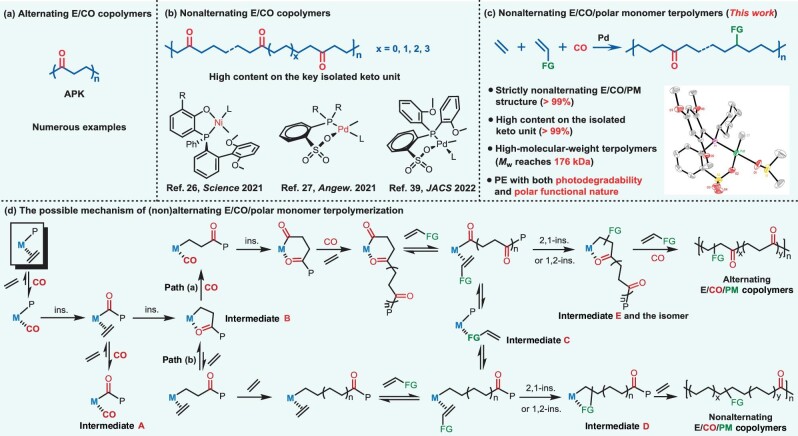
(a–c) Pioneering breakthroughs in E/CO copolymerizations, and this work. E: ethylene; CO: carbon monoxide; PM: polar monomer; FG: functional group. (d) A general mechanistic picture of the terpolymerization of ethylene, CO and polar monomer. The process of the formation of non-alternating E/CO/PM terpolymers in this work.

To overcome the non-degradability of PE materials, a promising approach is to install cleavable groups into the in-chain structure of PEs. The catalytic copolymerization of ethylene (E, C2 resource) with carbon monoxide (CO, C1 resource) stands out [11,12] because the incorporated in-chain keto groups bestow photodegradability to the formed PEs via Norrish-type chain scission mechanisms [13–15]. Starting from the radical route [16–18], coordination–insertion copolymerization promoted by the extensively used Group 10 metal catalysts has been studied. The resulting polymers (Fig. 1) evolve from a strictly alternating polyketone (APK, a material that is difficult to process; *T*_m_ ≈ 260°C), low-molecular-weight non-alternating polyketone and moderately non-alternating high-molecular-weight polyketone, to a recently developed non-alternating copolymer with a low keto content [11,12,19–27]. PEs with a low content of isolated keto groups from the non-alternating copolymerization of E and CO are highly desired because they not only retain desirable bulk material properties but also possess photodegradability [26,27]. Furthermore, to overcome the non-polarity shortcoming of PE materials, a prospective approach is the direct copolymerization of E with polar monomer (PM), which readily introduces polar functional groups into the main chain of PEs [7–11,28,29]. These copolymerizations are also achieved by using Group 10 metal catalysts via a coordination–insertion mechanism; however, the formed polymers typically possess low molecular weights.

Although extensive progress on the copolymerization of E + CO, E + PM and CO + PM has been made, the terpolymerization of E + CO + PM is a great challenge due to the formation of undesired chelates originating from both CO and PM. Thus far, only one successful terpolymerization of E + CO + methyl acrylate has been reported via a coordination–insertion mechanism, although the polymer produced was strictly alternating and featured a very low molecular weight of 3.1 kg mol^−1^ [30,31]. Likewise, the structurally complicated E/CO/acrylate terpolymers with long-chain branchings via a free-radical mechanism were also synthesized at harsh conditions such as 190°C and 2000 bar, and could be used as modifiers or plasticizers for poly(vinyl chloride) (PVC) and poly(acrylonitrile-*co*-butadiene-*co*-styrene) (ABS) [32–34]. However, this strictly non-alternating terpolymerization of E + CO + PM is long-sought but still inaccessible, and such PE materials bearing both photodegradability and polarity remain elusive (Fig. 1).

Here, we report the synthesis of high-molecular-weight high-density PEs with a low content of in-chain isolated keto groups via the non-alternating copolymerization of E with CO using Group 10 catalysts. By using the preferred catalyst, the elusive non-alternating terpolymerization of E, CO and the PM is achieved, yielding high-molecular-weight PEs with a desirably low content of both in-chain isolated keto groups and main-chain polar functional groups. In addition, the polymer properties of the PEs are thoroughly studied.

## RESULTS

### Mechanistic considerations

As elucidated in Fig. 1d, a minimum of five intermediates (**A**–**E**) need to be solved in the terpolymerization of E, CO and a PM [7,11,12,30,35–39]. Due to the stronger binding affinity of CO than that of E, the coordination of CO (**A**) and the chelated keto group via a stable five-membered structure (**B**) inhibits the consecutive insertions of E despite the low CO concentration. Notably, the kinetic preference for CO incorporation easily promotes the formation of alternating copolymers instead of the non-alternating structures. Similarly, the σ-coordination of the PM (**C**) and the chelated functional group (**D**) derived from the incorporated PM impedes the required chain growth. In terms of monomer reactivity, CO is more reactive than E; however, the PM is less reactive. Notably, the presence of intermediates **A–E** typically lowers catalytic activities and polymer molecular weights. All these issues considerably limit the achievement of the desired terpolymerization. Mechanistic insights into copolymerizations of E + PM, E + CO and PM + CO can be found in previous works [30,35–39]. According to the general mechanism in Fig. 1, the catalyst that has a weak affinity toward the free CO and the chelated keto moiety is key to the non-alternating terpolymerization reaction (path b), while reaction conditions such as overall high E + CO pressure with low partial pressure of CO are paramount to favorable non-alternating chain growth (path b).

### Catalyst structure

Regarding the problem of the strong binding affinity of CO and the chelated keto group toward the metal center, the neutral, less-oxophilic Group 10 metal catalysts exhibit a less pronounced preference toward CO binding; thus, these catalysts are promising candidates in this polymerization. These candidate catalysts should tolerate the functional group of PMs while having the ability to copolymerize the PM in the presence of both E and CO. Therefore, neutral phosphinesulfonato palladium(II) catalysts **Pd1–Pd4** were used to potentially mediate the non-alternating terpolymerization of E, CO and the PM. Typically, α-diimine cationic Pd(II) catalysts **Pd5–Pd7**, which are eminent in ethylene polymerization, were selected for comparison (Fig. 3). Previously reported **Pd1–Pd3** and **Pd5–Pd7** were synthesized according to literature procedures [39–47]. The new catalyst, **Pd4**, was prepared using a similar method [48,49] and characterized by multiple techniques, including ^1^H/^13^C/^31^P nuclear magnetic resonance (NMR) spectroscopy, elemental analysis and X-ray diffraction analysis (Fig. 1c, and Supplementary Figs 1–3).

### Non-alternating copolymerization of E and CO

**Figure 2.**
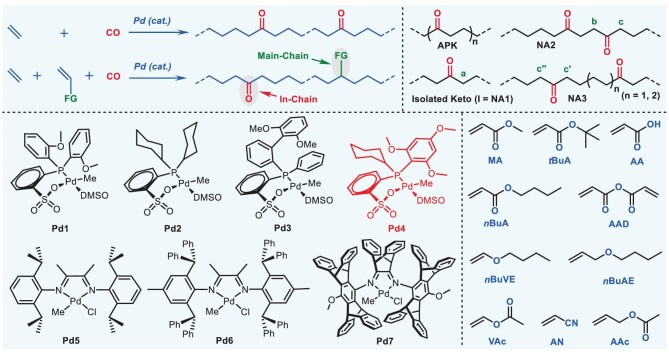
Copolymerization of E with CO and terpolymerization of E, CO, with polar monomer. This figure shows seven palladium catalysts **Pd1–Pd7** and ten fundamental polar monomers used in this work, and alternating polyketone (APK) and the non-alternating (NA) structures of NA1 (the isolated keto structure), NA2 and NA3.

**Figure 3.**
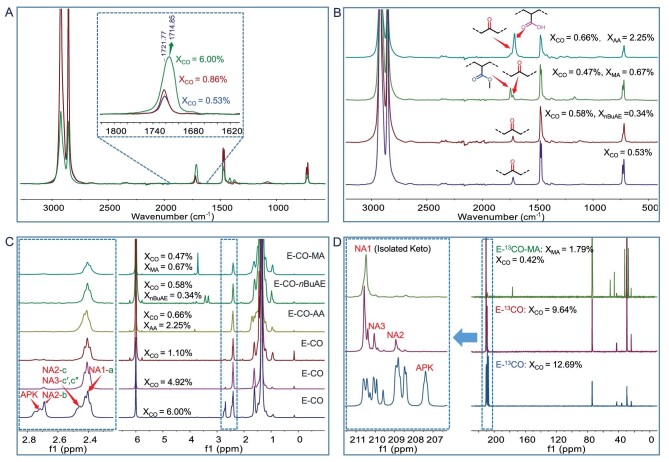
Structural analysis on E/CO and E/CO/PM polymers. (A) IR spectra of E/CO copolymers; Table 1, entries 1, 4 and 5. Reference value of APK: 1692 cm^−1^. Strictly isolated keto structure: 1723 cm^−1^. (B) IR spectra of E/CO/PM terpolymers Table 1, entry 1; Table 2, entries 2, 11 and 18. (C) ^1^H NMR spectra of copolymers and terpolymers; Table 1, entries 2, 3 and 5; Table 2, entries 2, 11 and 18. APK: 2.70–2.77 ppm; NA1: 2.41 ppm (**a**, isolated keto group), NA2: 2.69 ppm (**b**), NA2 + NA3: 2.42–2.51 ppm (**c, c'** and **c''**). (D) ^13^C NMR spectra of ^13^CO-labeled copolymers and terpolymers. (Bottom) **Pd1** (5 μmol), 80°C, 1 h, ^13^CO (30 mL), ethylene (20 bar); (middle) **Pd3** (2 μmol), 80°C, 1 h, ^13^CO (10 mL), ethylene (20 bar); (top) **Pd4** (10 μmol), 100°C, 1 h, ^13^CO (8 mL), ethylene (20 bar), MA (0.3 mol L^−1^).

Typically, the non-alternating copolymerization of E and CO yielded both the APK structure and the non-alternating (NA) copolymer structures, including distinguishable NA1 (the isolated keto unit), NA2 and NA3 (for the structural assignment, see Fig. 3). To maximize the photodegradation of PE materials, the content of the isolated keto unit (I = NA1, as shown in Fig. 2) in the incorporated CO groups should be as high as possible, resulting in spread-out keto groups incorporated into the PE chain. Mecking reported a high content of isolated keto unit [I/(NA + A); I = NA1, NA = NA1 + NA2 + NA3, A = APK] in nickel(II)-catalyzed E/CO copolymerization [29]. Nozaki disclosed a moderate content of isolated keto unit for E/CO copolymerization and a significantly higher content for palladium(II)-catalyzed copolymerization of E with metal carbonyls Fe_2_(CO)_9_ and Mn_2_(CO)_10_ (Fig. 1) [27]. Thus, achieving outstanding selectivity of the isolated keto unit for E/CO copolymerizations remains challenging.

Under the overall E + CO pressure of 20 bar with a low CO concentration of 0.1%, **Pd1–Pd4** were active at 80°C in the copolymerization of E and CO (Table 1, entries 1–4). The formed copolymers were strictly non-alternating E/CO copolymers [NA/(A + NA) > 99%] with a desirably low keto content of 0.53–4.92 mol%, as identified by both infrared radiation (IR) and NMR spectroscopy (see below). Notably, a significantly high content of the isolated keto unit [I/(A + NA) reached 96.5%] was key to retaining the bulk material properties of these PEs with low keto content (see below). Under otherwise identical conditions, an increase in the CO gas concentration from 0.1% to 0.3% in the feed E + CO gas led to a significant enhancement of CO incorporation (4.48–11.7 mol%); however, the change inevitably resulted in decreased contents of both the non-alternating structure and the isolated keto unit (Table 1, entries 5–8). Thus, an alternating polyketone structure was observable. As anticipated, the molecular weights of the resulting polymers also decreased. These findings revealed the crucial role of CO concentration in the feed gas in producing PEs with a high content of the desired isolated keto unit. In the data, **Pd3** and **Pd4** performed notably in the presence of 0.1% CO (E + CO pressure: 20 bar), enabling the formation of high-molecular-weight copolymers (*M*_w_ = 170 and 124 kg mol^−1^) accompanied by a strictly non-alternating structure (>99%) and a high content of the isolated keto unit (96.5% and 94.1%).

**Table 1.** Non-alternating copolymerization of ethylene and CO.^a^

Entry	cat. (umol)	t (min)	Yield (mg)	act.^b^	*M*_w_ (10^3^)^c^	*M*_w_/*M*_n_^c^	X_CO_^d^ (mol%)	NA/(A+NA)^d^	I/(A+NA)^d^	*T*_m_^e^ (°C)
1	**Pd1** (5)	20	960	5.76	40.4	1.90	0.53	>99%	73.9%	131.4
2	**Pd2** (5)	30	56	0.22	13.7	1.65	4.92	>99%	87.3%	127.6
3	**Pd3** (5)	18	475	3.17	170	1.57	1.10	>99%	96.5%	135.7
4	**Pd4** (5)	30	540	2.16	124	1.53	0.86	>99%	94.1%	135.6
5^f^	**Pd1** (5)	50	270	0.65	32.4	2.12	6.00	81.0%	38.5%	131.1
6^f^	**Pd2** (10)	50	70	0.08	11.2	1.58	11.7	97.3%	67.8%	123.0
7^f^	**Pd3** (5)	50	380	0.91	138	1.57	4.48	97.8%	66.8%	132.6
8^f^	**Pd4** (10)	50	274	0.33	59.4	1.52	4.76	97.5%	78.4%	133.0
9^g^	**Pd4** (2)	10	1600	48.0	221.8	2.07	-	-	-	137.7

^a^Reaction conditions: CO/ethylene (0.1%, 20 bar), toluene (100 mL), temperature (80°C), all entries are based on at least two runs, unless noted otherwise.

^b^Activity is in the unit of 10^5^ g mol^−1^ h^−1^.

^c^Determined by GPC in 1,2,4-trichlorobenzene at 150°C vs. linear polystyrene standards, and corrected by universal calibration.

^d^The incorporation ratio of CO and the value of NA/(A + NA) determined by ^1^H NMR analysis; NA = NA1 + NA2 + NA3, A = APK.

^e^Determined by DSC (second heating).

^f^CO/ethylene (0.3%, 20 bar).

^g^ethylene (20 bar) without the addition of CO.

### Non-alternating terpolymerization of E, CO and PMs

The performance of the catalysts was evaluated in terms of the polymer molecular weight and the content of the non-alternating structure and the isolated keto unit. Preferred catalysts **Pd3** and **Pd4** were further tested for the more challenging non-alternating terpolymerization of E, CO and fundamental PMs. In the presence of a high concentration of methyl acrylate (MA, 0.3 mol L^−1^), **Pd3** was able to mediate the terpolymerization reaction, producing a high-molecular-weight terpolymer (152 kg mol^−1^; Table 2, entry 1). The polymer structure was strictly non-alternating (>99%), and the isolated keto unit was predominant (94.0%). However, the desired incorporation of MA was quite low (0.20 mol%), which should be attributed to the large steric bulk of the ligand in **Pd3**.

**Table 2.** Strictly non-alternating terpolymerization of CO and ethylene with polar monomers.^a^

Entry	cat. (umol)	*T* (°C)	PM (mol L^−1^)	Yield (mg)	act.^b^	*M*_w_^c^ (10^3^)	*M*_w/_ *M*_n_^c^	X_PM_ (mol%)^d^	X_CO_ (mol%)^d^	NA/(A + NA)^d^	I/(A + NA)^d^	*T*_m_^e^ (°C)
1	**Pd3** (2)	80	MA (0.3)	160	0.80	152	1.48	0.20	3.10	>99%	94.0%	128.2
2	**Pd4** (10)	80	MA (0.1)	1110	1.11	158	2.08	0.67	0.47	>99%	93.6%	122.8
3	**Pd4** (10)	80	MA (0.15)	690	0.69	138	2.34	0.90	0.80	>99%	93.5%	120.9
4	**Pd4** (10)	80	MA (0.2)	120	0.12	45.9	2.41	1.20	3.99	>99%	93.2%	118.2
5	**Pd4** (10)	100	MA (0.3)	280	0.28	53.8	2.32	2.38	1.68	>99%	95.0%	112.3
6^g^	**Pd5** (10)	60	MA (0.15)	9	0.01	-	-	1.24	32.7	6.6%	<0.1%	-
7^g^	**Pd6** (10)	80	MA (0.15)	25	0.03	12.1	2.02	0.12	6.41	22.3%	<0.1%	94.3
8^g^	**Pd7** (10)	80	MA (0.15)	trace	-	-	-	-	-	-	-	-
9	**Pd4** (10)	80	*t*BuA (0.15)	1900	1.90	176	1.90	0.40	0.37	>99%	>99%	125.2
10	**Pd4** (10)	100	*n*BuA (0.3)	1390	1.39	87.9	1.88	1.52	0.41	>99%	>99%	115.5
11	**Pd4** (20)	100	AA (0.3)	460	0.23	27.8	3.06	2.25^f^	0.66^f^	>99%	>99%	116.3
12	**Pd4** (20)	100	AAD (0.15)	128	0.06	14.8	1.79	1.78	3.90	>99%	93.5%	107.2
13	**Pd4** (10)	100	*n*BuVE (0.3)	3510	3.51	107	1.74	0.18	0.18	>99%	>99%	128.9
14	**Pd4** (20)	100	AN (0.15)	94	0.05	10.3	1.90	0.94^f^	4.94	>99%	92.3%	120.9
15	**Pd4** (10)	100	VAc (0.3)	106	0.11	32.9	2.56	<0.1	4.26	>99%	93.0%	129.6
16	**Pd4** (20)	100	AAc (0.15)	33	0.02	10.1	3.03	0.24	6.30	>99%	90.5%	121.1
17	**Pd4** (10)	80	*n*BuAE (0.15)	230	0.23	56.2	1.83	0.20	2.08	>99%	93.3%	127.8
18	**Pd4** (20)	100	*n*BuAE (0.15)	860	0.43	56.1	1.69	0.34	0.58	>99%	>99%	125.1

^a^Reaction conditions: CO/ethylene (0.1%, 20 bar), toluene (100 mL), time (60 min), radical inhibitor: galvinoxyl (10 mg), all entries are based on at least two runs, unless noted otherwise.

^b^Activity is in the unit of 10^5^ g mol^−1^ h^−1^.

^c^Determined by GPC in 1,2,4-trichlorobenzene at 150°C vs. linear polystyrene standards, and corrected by universal calibration.

^d^Determined by ^1^H NMR analysis.

^e^Determined by DSC (second heating).

^f^Determined by ^13^C NMR analysis.

^g^1.5 eq. NaBArF.

To enhance the incorporation of the PM, we sought out the new catalyst, **Pd4**, and tested it in terpolymerization reactions. Even in the presence of a lower concentration of MA (0.1 mol L^−1^), the incorporation of MA was 3.3 times higher than that achieved with **Pd3**, significantly increasing to 0.67 mol% (Table 2, entry 2), while retaining the strictly non-alternating polymer structure (>99%), in which the isolated keto unit was again predominant (93.6%). To compare the terpolymerization ability of milestone cationic α-diimine Pd(II) catalysts, **Pd5–Pd7** were tested under similar conditions (Table 2, entries 6–8). Extremely low activities were observed, with a product yield of 9–25 mg, contents of the non-alternating structure only reaching 6.6%–22.3%, and no isolated keto units being found. This could be attributed to the more stable cationic five-membered chelate (Fig. 1d, intermediate B) relative to the neutral five-membered chelate. The cationic five-membered chelate could not be easily opened by ethylene and low-pressure CO, thus leading to very low activity. This highlighted the importance of the catalysts and their more challenging application to E/CO/PM terpolymerization than E/CO or E/PM copolymerization.

Due to the significantly higher MA incorporation, the preferred palladium catalyst, **Pd4**, was thus extensively studied for terpolymerization. As expected, increasing the MA concentration resulted in enhanced MA incorporation (0.67–1.20 mol%) in the polymers (Table 2, entries 2–4). Importantly, the molecular weights of the formed polymers were high (45.9–158 kg mol^−1^). Interestingly, at the same temperature the elevated concentration of MA favored the reactivity of CO with enhanced incorporation (0.47–3.99 mol%) into the strictly non-alternating polymers with predominant isolated keto units. This was indicative of increased incorporations of both MA and CO at the same temperature (Table 2, entries 2–4). In contrast, elevating the temperature led to a decrease in CO incorporation but an increase in MA incorporation (Table 2, entries 4 vs. 5 and also for *n*-butyl allyl ether (*n*BuAE) in the following entries, 17 vs. 18). These indicated the effects of enchained/free polar monomer and reaction temperature on the CO insertion process. The highest incorporation (2.38 mol%) of MA was 0.3 mol L^−1^ at 100°C (Table 2, entry 5).

Under otherwise identical conditions, replacing MA with bulkier *tert*-butyl acrylate (*t*BuA) or *n*-butyl acrylate (*n*BuA) resulted in both enhanced activities and increased molecular weights (87.9–176 kg mol^−1^) but decreased incorporations of both the PM and CO (Table 2, entries 9 vs. 3, 10 vs. 5). As shown in Fig. 1d, the presence of both the PM and CO doubly lowered the molecular weight of the polymers formed; thus, high-molecular-weight terpolymers were not easily produced. Notably, a high content of both the non-alternating structure (>99%) and isolated keto units (>99%) were achieved. The more difficult terpolymerization of acrylic acid (AA) was also achieved to produce a polymer with a high content of >99% (Table 2, entry 11). Moreover, the participation of acrylic anhydride (AAD) in terpolymerization was enabled (Table 2, entry 12).

In addition to the acrylic monomer series, the fundamental PM—*n*-butyl vinyl ether (*n*BuVE)—was tested using **Pd4** under similar reaction conditions (Table 2, entry 13). The incorporation of both *n*BuVE and CO was observable, and the polymer molecular weight was high (107 kg mol^−1^). Notably, both the strictly non-alternating structure (>99%) and completely isolated keto unit (>99%) were observed in the terpolymer. Moreover, **Pd4** promoted acrylonitrile (AN) terpolymerization (Table 2, entry 14) despite its extremely low activity and molecular weight, which can be attributed to the strong cyano group chelate. When vinyl acetate (VAc) was used as the PM, terpolymerization proceeded, but no VAc incorporation was detected (Table 2, entry 15).

In addition to polar vinyl monomers, polar allyl monomers were subjected to terpolymerization. Although VAc did not incorporate into the polymer, allyl acetate (AAc) was successfully inserted into the strictly non-alternating polymer at 0.24 mol% incorporation (Table 2, entry 16) [50]. Compared to that in the AAc reaction, significantly higher activity was found for *n*-butyl allyl ether (*n*BuAE) terpolymerization, yielding higher molecular weight polymers incorporated with both *n*BuAE (0.34 mol%) and CO (0.58 mol%) (Table 2, entries 17 and 18). Notably, both contents of the non-alternating structure and the isolated keto unit were >99%.

### Structural analysis on polymers

The microstructures of the E/CO copolymers and E/CO/PM terpolymers were fully characterized by IR spectroscopy, NMR spectroscopy, including ^1^H/^13^C/2D NMR methods, the ^13^CO-labeling technique, differential scanning calorimetry (DSC), thermogravimetric analysis (TGA), wide-angle X-ray diffraction (WXRD) analysis, the tensile test and water contact angle (WCA) measurement. In the IR spectra, the carbonyl (C=O) stretching resonance frequency for pure APK appeared at ∼1692 cm^−1^ [26], whereas that for the strictly isolated keto-modified PE was observed at ∼1723 cm^−1^. This result indicated that the spatial separation between two adjacent incorporated carbonyls in PEs caused the IR resonances (C=O) to gradually shift to a larger wavenumber (blue shift). As shown in Fig. 3A, the relatively low content (38.5%) of the isolated keto unit in the high CO incorporation (6.00 mol%) sample (E/CO copolymer) was attributable to a resonance at 1715 cm^−1^, and the resonance appearing at 1722 cm^−1^ implied a high content (up to 94.1%) of the isolated keto unit. In the E/CO/*n*BuAE terpolymer, only one C=O resonance appeared at 1723 cm^−1^; however, as expected, two C=O resonances appeared at 1723 and 1745 cm^−1^ (COOMe) in the E/CO/MA terpolymer and at 1723 and 1709 cm^−1^ (COOH) in the E/CO/AA terpolymer (Fig. 3B). Again, these terpolymers possessed a high content of the isolated keto unit, as evidenced by the resonance appearing at 1723 cm^−1^.

^1^H NMR spectra provided more detailed information. The E/CO sample corresponding to Table 1 (entry 5) was specially selected for analysis because of its low selectivity. As elucidated in Fig. 3C, the resonance at 2.70–2.77 ppm was assigned to the APK structure; the triplet appearing at 2.41 ppm originated from the isolated keto group (**a**, NA1), the singlet appearing at 2.69 ppm arose from the non-alternating structure NA2 (**b**), and the multiple signals at 2.42–2.51 ppm were attributed to two different and distinct non-alternating structures (**c**, NA2; **c**′ and **c**″, NA3). The disappearance of the signals at 2.70–2.77 ppm (APK) indicated the presence of a strictly non-alternating structure, while the appearance of the sole signal at 2.41 ppm revealed an isolated-keto-group content of >99%. These resonances derived from polar functional groups were also observed (Supplementary Figs 12–33). Notably, negligible olefinic endgroups originating from β-H eliminations in the ^1^H NMR spectra suggested high molecular weights, as confirmed by gel permeation chromatography (GPC) curves. Apparently, the presence and incorporation of both CO and PM did not promote problematic chain transfer reactions. In addition, ^13^C NMR analysis indicated linear polymer microstructures devoid of branches. To enhance the sensitivity of the characteristic carbonyl C=O group to ^13^C NMR spectroscopy, three co- and terpolymerizations were performed under a ^13^CO atmosphere (Fig. 3D). The ^13^C-labeled APK structure appeared at 207.4 ppm, and the ^13^C-labeled isolated keto unit (NA1) gave rise to a resonance at 210.5 ppm. Moreover, the remaining non-alternating structures resonated at 208.4–210.4 ppm. Again, increasing the distance of two adjacent carbonyls in the PE chain led to a down-field shift of the C=O signal, which agreed with the aforementioned IR spectral result. Figure 3D clearly suggests the strictly non-alternating structure and high content of the isolated keto unit in the E/^13^CO/MA terpolymer obtained under reaction conditions similar to those of E/CO/MA terpolymerization (Table 2, entry 5). The results of IR, ^1^H NMR and ^13^C NMR spectroscopy agreed with each other, thus confirming the presence of the microstructures of E/CO copolymers and E/CO/PM terpolymers.

### Polymer properties

**Figure 4.**
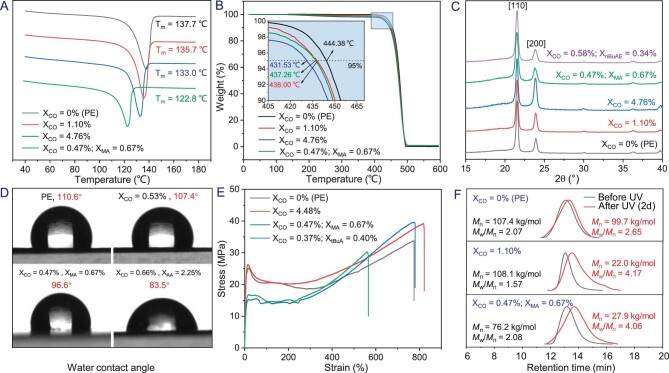
Comparison of polymer properties between PE, PE with in-chain isolated keto group, and PE with in-chain isolated keto and main-chain functional groups. (A) DSC curves of Table 1, entries 3, 8 and 9; Table 2, entry 2. (B) (e) TGA curves of Table 1, entries 3, 8 and 9; Table 2, entry 2. (C) WXRD analyses of Table 1, entries 3, 8 and 9; Table 2, entries 2 and 18. (D) Water contact angles of Table 1, entries 1 and 9; Table 2, entries 2 and 11. (E) Tensile tests of Table 1, entries 7 and 9; Table 2, entries 2 and 9. (F) Photodegradability of Table 1, entries 3 and 9; Table 2, entry 2.

The promising properties of the E/CO copolymers and E/CO/PM terpolymers were further studied for comparison with those of the bulk PE. The DSC profile (Fig. 4A) of the E/CO copolymer sample with a low keto content (1.10 mol%) indicated a slight depression of the melting point in relation to that of bulk PE (*T*_m_ = 137.7°C vs. 135.7°C). Even at a high keto content of 4.76 mol%, the *T*_m_ of the copolymer retained a high value of 133.0°C, which was attributed to both the high content of the isolated keto unit that did not disturb the crystalline structure of PE, and the high molecular weight of copolymer. A high *T*_m_ (122.8°C) was also observed when both CO and the PM were incorporated into PE. TGA data (Fig. 4B) further verified the high thermal decomposition temperatures (*T*_d_ > 430°C) of the generated E/CO copolymers and E/CO/PM terpolymers, which were only slightly lower than those of bulk PE. Both *T*_m_ and *T*_d_ implied processing similar to that of PE instead of APK. In addition, WXRD analysis revealed that the solid-state structures of these copolymers and terpolymers were virtually identical to that of PE (Fig. 4C).

The WCA was preliminarily measured to evaluate the importance of polar functional groups on the surface of materials (Fig. 4D). As expected, the incorporation of a low amount of CO slightly improved the surface of PE materials, while the introduction of PMs such as MA and AA significantly impacted the polarity of PE materials. A large difference of 27° in the WCAs was observed (110.6° vs. 83.5°).

The high-molecular-weight characteristic enabled tensile testing (Fig. 4E). Pure PE showed yield strength *σ*_Y_ of 25.1 MPa (tensile strength: 33.8 MPa), while the E/CO copolymers with 4.48% CO content exhibited *σ*_Y_ of 26.3 MPa (tensile strength: 39.2 MPa). Moreover, high *σ*_Y_ of 15.2 (tensile strength: 39.6 MPa) (X_CO_ = 0.47 mol%, X_MA_ = 0.67 mol%) and 16.7 MPa (tensile strength: 30.3 MPa) (X_CO_ = 0.37 mol%, X_tBuA_ = 0.40 mol%) were observed in the E/CO/PM terpolymers.

Finally, due to the presence of in-chain keto moieties, the photodegradabilities of the copolymers and terpolymers was tested by irradiating the polymer film with UV light (λ = 255/275 nm) at an intensity of 12 mW cm^−2^ at 30°C for 2 days. As anticipated, molecular weights significantly reduced in the copolymers (∼5 times lower) and terpolymers (∼3 times lower), while molecular weight distributions clearly broadened. By testing the samples of E/CO copolymer and E/CO/MA terpolymer after photodegradation, one new signal at δ = 9.80 ppm was observed in ^1^H NMR spectra (cf. Supplementary Data), which could be attributed to the generated terminal aldehyde CHO group. By contrast, as a control experiment, pure PE exhibited a slightly altered molecular weight (Fig. 4F). These comprehensive studies revealed that the low incorporation of the in-chain isolated keto group allowed the retention of the desirable properties of PE while bestowing photodegradability to the materials. Moreover, the addition of the polar functional group significantly improved the surface properties of the materials.

## CONCLUSION

To prevent the formation of undesirable diverse chelates that limit both CO and PM copolymerizations, we developed Group 10 metal catalysts that enable the strictly non-alternating terpolymerization of ethylene, CO and fundamental PMs. Both the catalyst and the low concentration of CO feed were key to the terpolymerization reactions producing high-molecular-weight PEs bearing a high content of isolated in-chain keto group and allowing the reasonable incorporation of the main-chain polar functional group. Owing to the high-molecular-weight characteristic and the low content of the strictly isolated keto group, the PE materials retained their desirable bulk properties while acquiring relatively dispersed break points to photodegrade the materials to the maximum extent. Moreover, the reasonable introduction of polar functional groups considerably improved the surface properties of PEs. We believe that this strictly non-alternating terpolymerization reaction of ethylene + CO + PM can contribute to the development of degradable plastics, which are needed for reducing the problematic environmental persistency of PE waste.

## MATERIALS AND METHODS

All materials and methods can be found in the Supplementary Data.

## Supplementary Material

nwad039_Supplemental_Files

## Data Availability

All data needed to evaluate the conclusions in the paper are present in the paper and/or the Supplementary Data. Additional data related to this paper may be requested from the authors.
