# Supplementary material for: Photodegradable polar-functionalized polyethylenes
Source: Natl Sci Rev. 2023 Feb 16;10(7):nwad039. doi: 10.1093/nsr/nwad039 (PMC10434297; doi:10.1093/nsr/nwad039)
Supplement: nwad039_Supplemental_Files [file nwad039_supplemental_files.zip › Supplementary information.pdf]

Supplementary Information

Photodegradable Polar Functionalized Polyethylenes

Chaoqun Wang,[a,b] Jian Xia,[a] Yuxing Zhang,[a,b] Xiaoqiang Hu,[a,b] and Zhongbao Jian*[a,b]

[a] State Key Laboratory of Polymer Physics and Chemistry, Changchun Institute of Applied Chemistry, Chinese Academy of Sciences, Renmin Street 5625, Changchun 130022, China.
[b] University of Science and Technology of China, Hefei 230026, China.

Contents

1. General Information 1
2. Synthesis of Catalysts 3
3. NNR Figures of Polymers 6
4. GPC Curves of Polymers 27
5. DSC Data of Polymers 40
6. IR spectra of copolymers 52
7. Contact angle measurements 54
8. TGA data of polymers 55
9. WXRD data of polymers 59
10. Tensile experiments 59
11. Photodegradation experiment 60
12. Crystallographic Data 65
13. References 66

1. General Information

Table S1. Effect of Polymerization Time on Nonalternating Terpolymerization of CO/Ethylene/MA [a]

entry	t (min)	T (℃)	yield (mg)	act. [b]	M_w [c] (10^3)	M_w/M_n [c]	X_{PM} (mol%) [d]	X_{CO} (mol%) [d]	T_m [e] (℃)
1	30	80	85	0.17	28.3	1.57	0.86	5.00	120.9
2	60	80	120	0.12	45.9	2.41	1.20	3.99	119.7
3	90	80	420	0.28	135.4	2.02	0.90	1.64	118.2

[a] Reaction conditions: **Pd4** (10umol), CO/ethylene (0.1%, 20 bar), MA (0.2mol/L), toluene (100 mL), radical inhibitor: galvinoxyl (10 mg), all entries are based on at least two runs, unless noted otherwise. [b] Activity is in unit of 10^5 g mol^{-1} h^{-1}. [c] Determined by GPC in 1,2,4-trichlorobenzene at 150 ℃ vs. linear polystyrene standards, and corrected by universal calibration. [d] Determined by ^1H NMR analysis. [e] Determined by DSC (second heating).

General Procedures: All syntheses involving air- and moisture-sensitive compounds were carried out using standard Schlenk-type glassware (or in a glove box) under an atmosphere of nitrogen. All solvents were purified from the MBraun SPS system. NMR spectra for the ligands, complexes, and polymers were recorded on a Bruker AV400 (^1H: 400 MHz, ^{13}C: 100 MHz, ^{31}P: 162 MHz, ^{19}F: 376 MHz) or a Bruker AV500 (^1H: 500 MHz, ^{13}C: 125 MHz, ^{31}P: 202 MHz, ^{19}F: 470 MHz). NMR assignments were confirmed by ^1H–^{13}C HSQC, ^1H–^1H COSY and ^1H–^{13}C HMBC experiments. The molecular weights (M_w) and molecular weight distributions (M_w/M_n) of polyethylenes were measured by means of gel permeation chromatography (GPC) on a PL-GPC 220-type high-temperature chromatograph equipped with three PL-gel 10 μm Mixed-B LS type columns at 160 ℃. Melting point temperature (T_m) of polyethylenes was measured through DSC analyses, which were carried out on a TA Q2000 DSC Instrument under a nitrogen atmosphere at heating and cooling rates of 10 ℃/min (temperature range: 20-180 ℃). Thermogravimetric analyses (TGA) were performed on a TA STD Q600 thermal analyser at a heating rate of 10 ℃/min. Elemental analysis was performed at the National Analytical Research Centre of Changchun Institute of Applied Chemistry. ATR-IR spectra were acquired on a VERTEX 70 Fourier Transform Infrared spectrometer. Tensile experiments were performed at 5 mm/min on an Electromechanical Universal Testing Machine (E43.104) at room temperature. Wide angle X-ray diffractograms (WXRD) were performed on a Rigaku SmartLab instrument. Analysis of water contact angles was performed on a Krüss Drop Shape Analyzer DSA100 with water. The photodegradation experimental device was purchased from Qingdao Jason Electric Co. (www.qdjason.com).

X-Ray diffraction: Data collections were performed at 294.8 K on a Bruker SMART APEX diffractometer with a CCD area detector, using graphite-monochromated Mo Kα radiation (λ = 0.71073 Å). The determination of crystal class and unit cell parameters was carried out by the

SMART program package. [1] The raw frame data were processed using SAINT and SADABS to yield the reflection data file. [2] All structures were solved and refined by full-matrix least-squares procedures on F^2 using SHELXTL or Olex2. [3] Refinement was performed on F^2 anisotropically for all non-hydrogen atoms by the full-matrix least-squares method. The hydrogen atoms were placed at the calculated positions and were included in the structure calculation without further refinement of the parameters.

A general procedure of polymerization using the Pd(II) catalyst: The polymerization experiments were performed in a *Büchi miniclave* reactor with a 200 mL vessel, which was equipped with a mechanical stirrer, a heating and cooling jacket connected to a thermostat, a thermocouple dipping into the polymerization mixture and a nitrogen/vacuum supply. The reactor connected with a high-pressure gas line was firstly dried at 100 ℃ under vacuum for at least 1 h. Adjust to the target temperature on the temperature control system. 98 mL of toluene was added to the reactor under an N_2 atmosphere, then, the desired amount of Pd(II) catalyst in 2 mL of toluene (and co-monomer in 2 mL of toluene) was injected into the polymerization system via syringe. With a rapid stirring, the reactor was pressurized and maintained at 20 bar of CO/ethylene (0.1% or 0.3%). After a specific time, the pressure reactor was vented and the polymerization was quenched via the addition of 100 mL acetone. The obtained polymer was washed 3 times with acetone and dried in a vacuum oven to constant weight.

2. Synthesis of Catalysts

Synthesis of Catalysts: Pd1, Pd2, Pd3, L4, Pd5, Pd6 and Pd7 were synthesized according to the reported works of literature. [4-9]

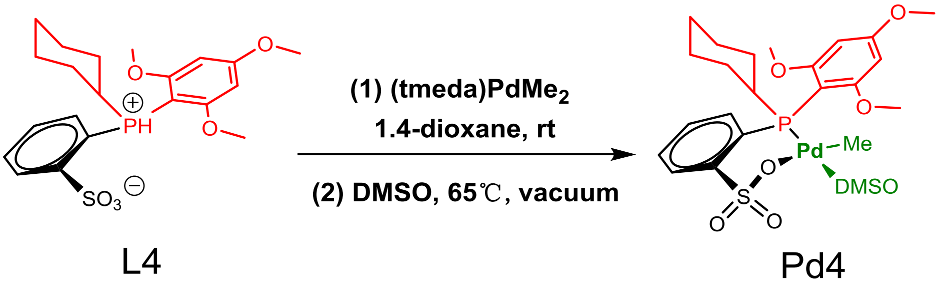

Synthesis of Pd4: The solution of **L4** (300 mg, 0.68 mmol) and (tmeda)$PdMe_2$ (171.8 mg, 0.68 mmol) in 1.4-dioxane (30 mL) was stirred for 24 h at room temperature. The white powder produced during the reaction was filtered out, dried in vacuum and added to 150 ml of DMSO. The mixture was stirred in vacuum at 65 ℃. Until the white solids were all dissolved, the solvent was further removed to give a beige solid, which was washed 3 times with ether and dried in vacuum. (350 mg, 80.8% yield).

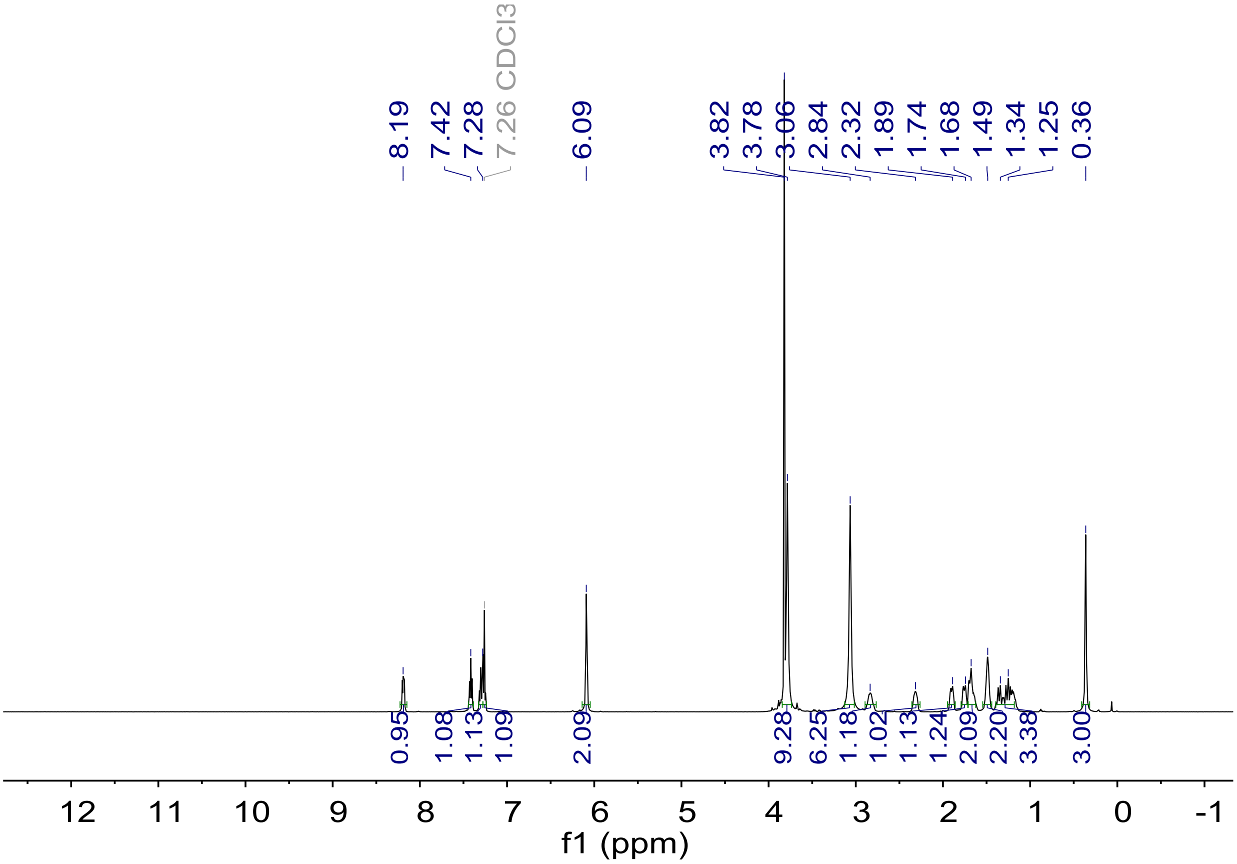

Fig. S1. ^{1}H NMR spectrum of **Pd4** in $CDCl_3$.

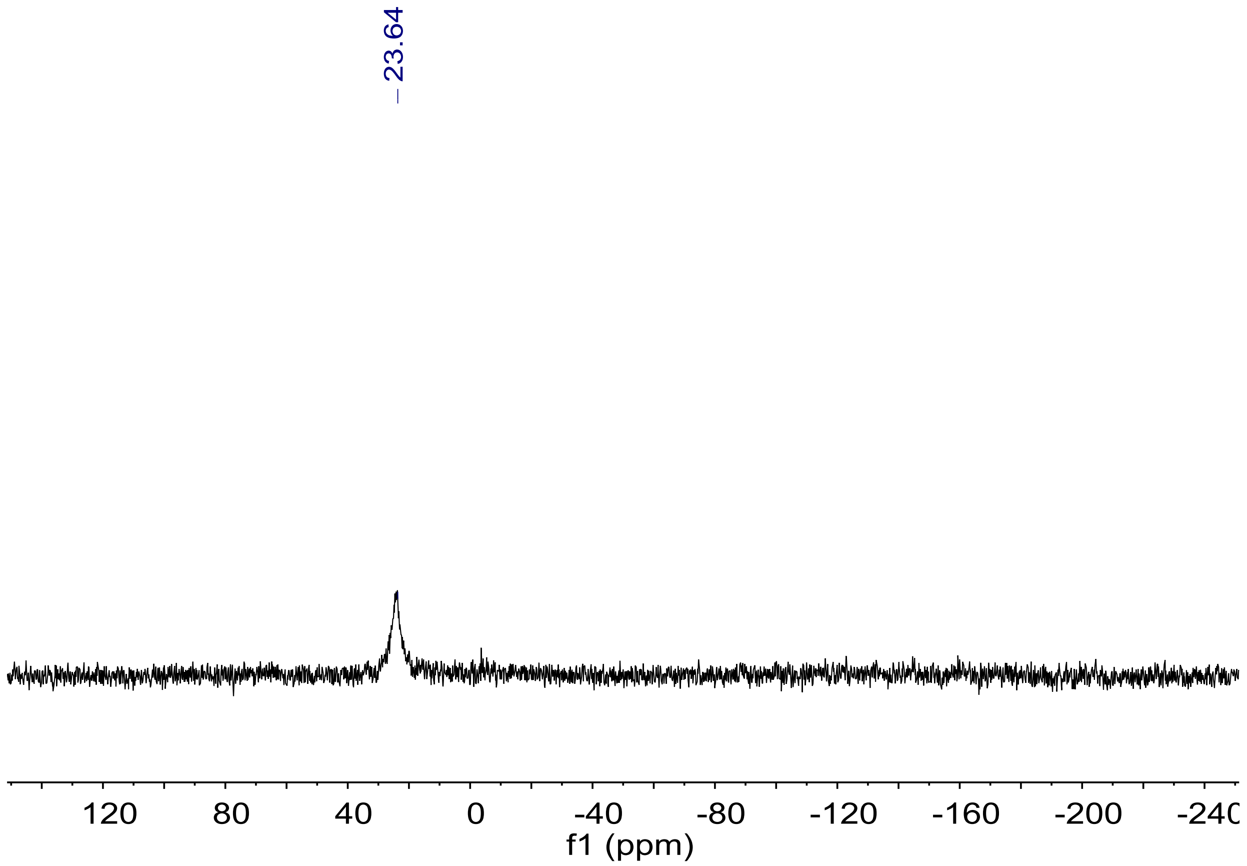

Fig. S2. ^{31}P NMR spectrum of **Pd4** in $CDCl_3$.

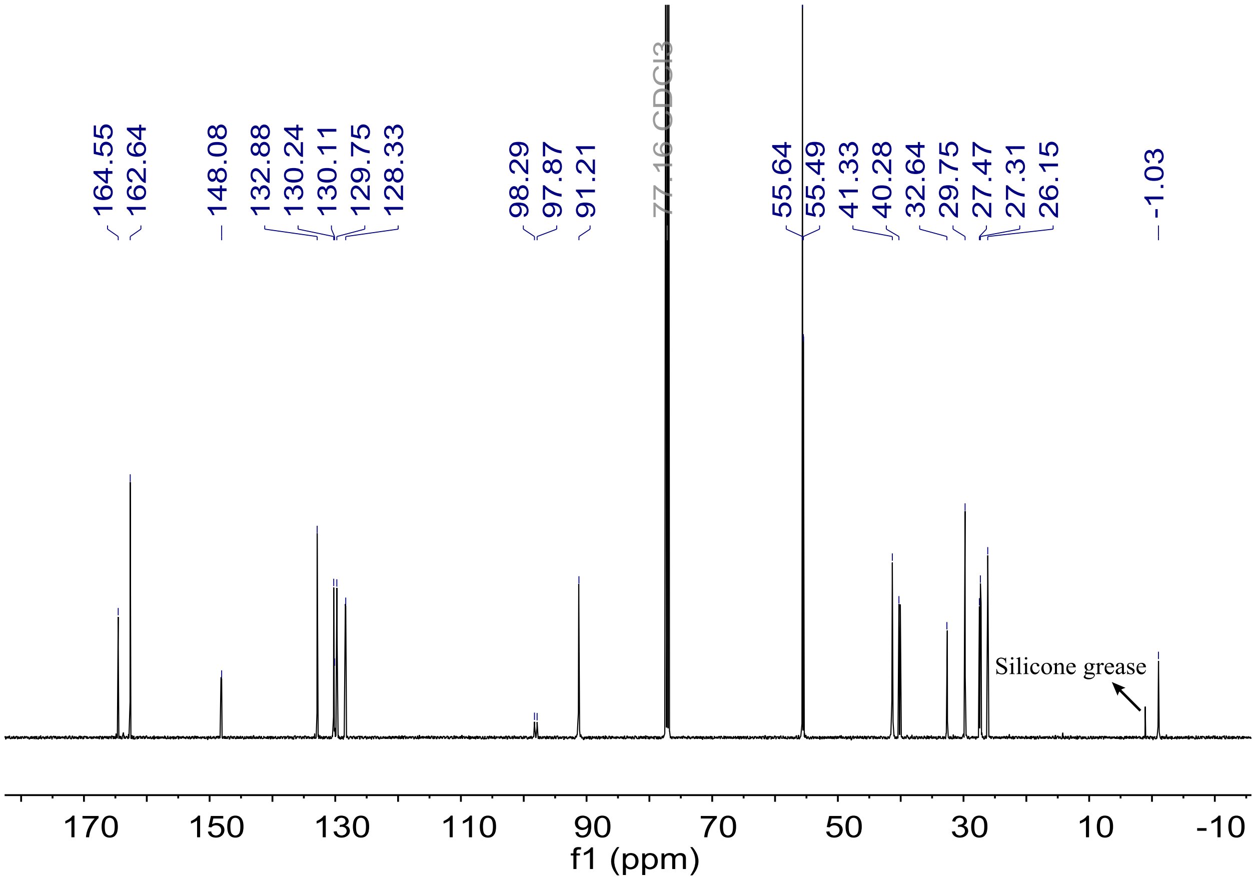

Fig. S3. ^{13}C NMR spectrum of **Pd4** in $CDCl_3$.

^{1}H NMR (500 MHz, 298 K, $CDCl_3$, 7.26 ppm): δ = 8.19 (dd, 1H, aryl-*H*), 7.42 (t, 1H, aryl-*H*), 7.28 (m, 2H, aryl-*H*), 6.09 (s, 2H, aryl-*H*), 3.82 (s, 6H, OCH_3), 3.78 (s, 3H, OCH_3), 3.06 (s, 6H, dmso-*H*), 2.84 (m, 1H, cyclohexane-*H*), 2.32 (m, 1H, cyclohexane-*H*), 1.89 (m, 1H, cyclohexane-*H*), 1.74 (m, 1H, cyclohexane-*H*), 1.68 (m, 2H, cyclohexane-*H*), 1.49 (m, 2H, cyclohexane-*H*), 1.25 (m, 3H, cyclohexane-*H*), 0.36 (s, 3H, Pd-CH_3).
^{31}P NMR (202 MHz, 298 K, $CDCl_3$, 7.26 ppm): δ = 23.64.
^{13}C{^{1}H} NMR (125 MHz, 298 K, $CDCl_3$, 77.16 ppm): δ = 164.55 (*C*-OCH_3), 162.64 (*C*-OCH_3), 148.08 (*C*-SO_3), 132.88 (P-*C*($PhSO_3$)), 130.24, 130.11, 129.75, 128.33, 98.29 (P-*C*($PhOMe_3$)), 91.21, 55.64 (OCH_3), 55.49 (OCH_3), 41.33 (S-CH_3), 40.28 (P-*C*H), 32.64 (CH_2), 29.75 (CH_2), 27.47 (CH_2), 27.31 (CH_2), 26.15 (CH_2), -1.03 (Pd-CH_3).
Elemental analysis: Anal. Calcd for $C_{24}H_{35}O_7PPdS2$: C, 45.25; H, 5.54; Found: C, 45.51; H, 5.42.

3. NNR Figures of Polymers

Take the polymer (from table 1, entry 5) as an example to calculate the values of X_{CO}, NA/(A+NA), and I/(A+NA).

$$X_{CO} = \frac{n(CO)}{n(CO)+n(Ethylene)} \times 100\%$$

$$= \frac{APK/4 + NA1/4 + 2\times NA2/4 + NA3/4}{(APK/4 + NA1/4 + 2\times NA2/4 + NA3/4)\times 2 + \mathrm{B}/4} \times 100\%$$

$$= \frac{1.46 + 2.96 + 2\times 1 + (5.23 - 2.96 - 1)}{[1.46 + 2.96 + 2\times 1 + (5.23 - 2.96 - 1)]\times 2 + 112.77} \times 100\% = 6.00\%$$

$$\frac{NA}{A+NA} = \frac{NA1/4 + 2\times NA2/4 + NA3/4}{APK/4 + NA1/4 + 2\times NA2/4 + NA3/4} \times 100\%$$

$$= \frac{2.96 + 2\times 1 + (5.23 - 2.96 - 1)}{1.46 + 2.96 + 2\times 1 + (5.23 - 2.96 - 1)} \times 100\% = 81.0\%$$

$$\frac{I}{A+NA} = \frac{NA1/4}{APK/4 + NA1/4 + 2\times NA2/4 + NA3/4} \times 100\%$$

$$= \frac{2.96}{1.46 + 2.96 + 2\times 1 + (5.23 - 2.96 - 1)} \times 100\% = 38.5\%$$

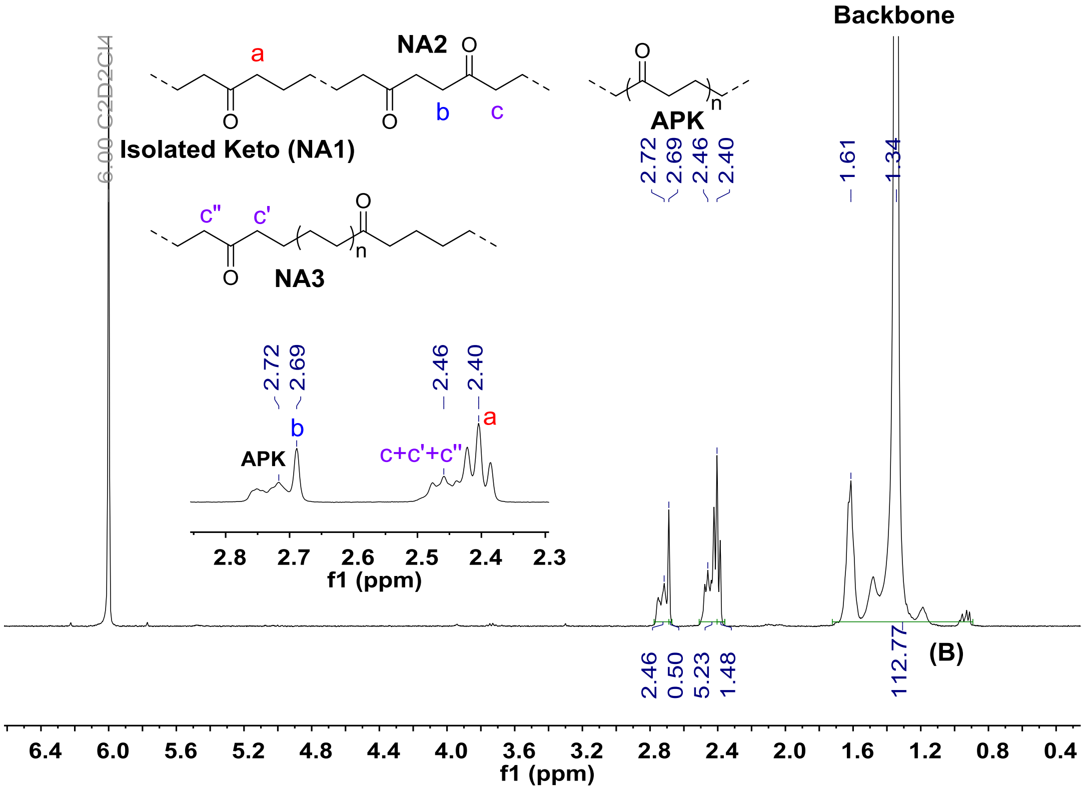

Figure S4 ^{1}H NMR spectrum (400 MHz, $C_2D_2Cl_4$, 110 ℃) of the polymer generated by complex **Pd1** from table 1, entry 5.

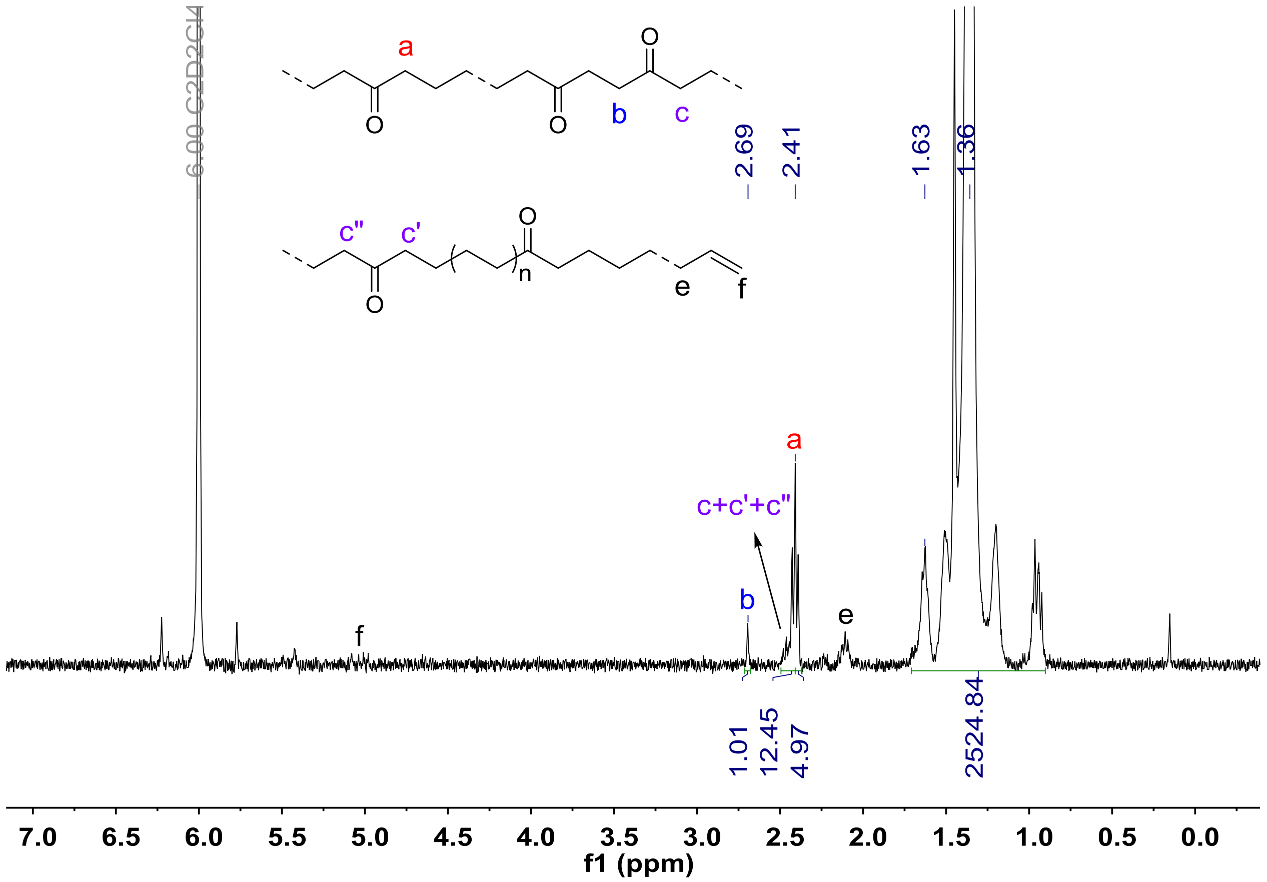

Figure S5. ^{1}H NMR spectrum (400 MHz, $C_2D_2Cl_4$, 110 ℃) of the polymer generated by complex **Pd1** from table 1, entry 1.

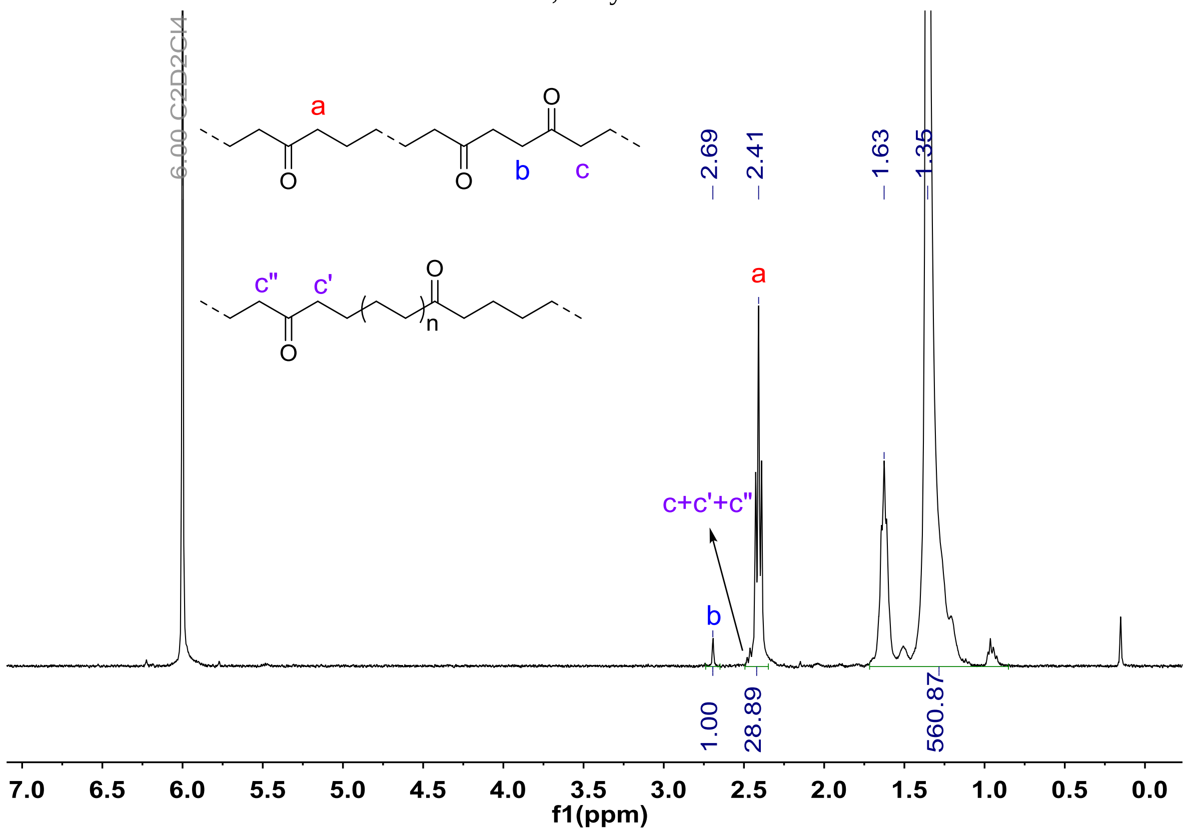

Figure S6. ^{1}H NMR spectrum (400 MHz, $C_2D_2Cl_4$, 110 ℃) of the polymer generated by complex **Pd2** from table 1, entry 2.

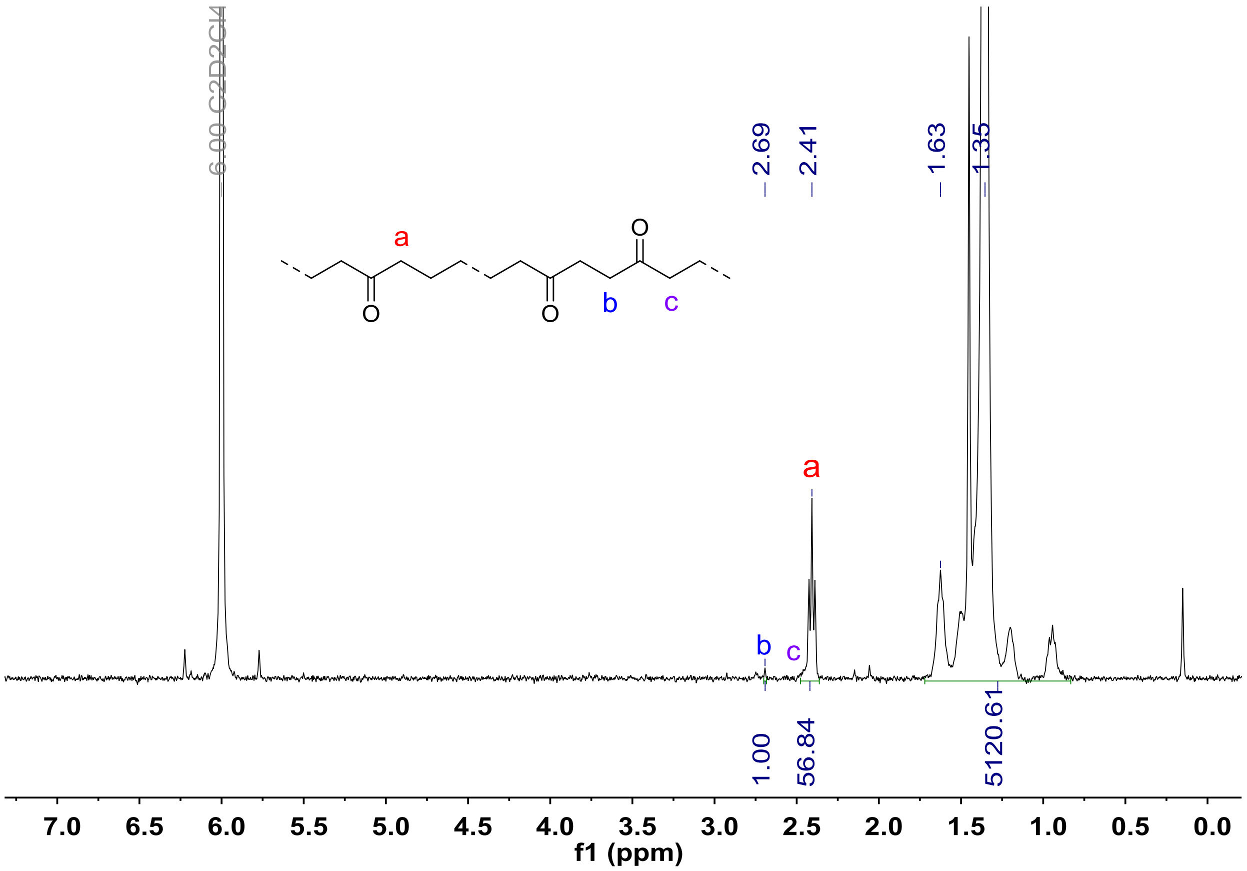

Figure S7. ^{1}H NMR spectrum (400 MHz, $C_2D_2Cl_4$, 110 ℃) of the polymer generated by complex **Pd3** from table 1, entry 3.

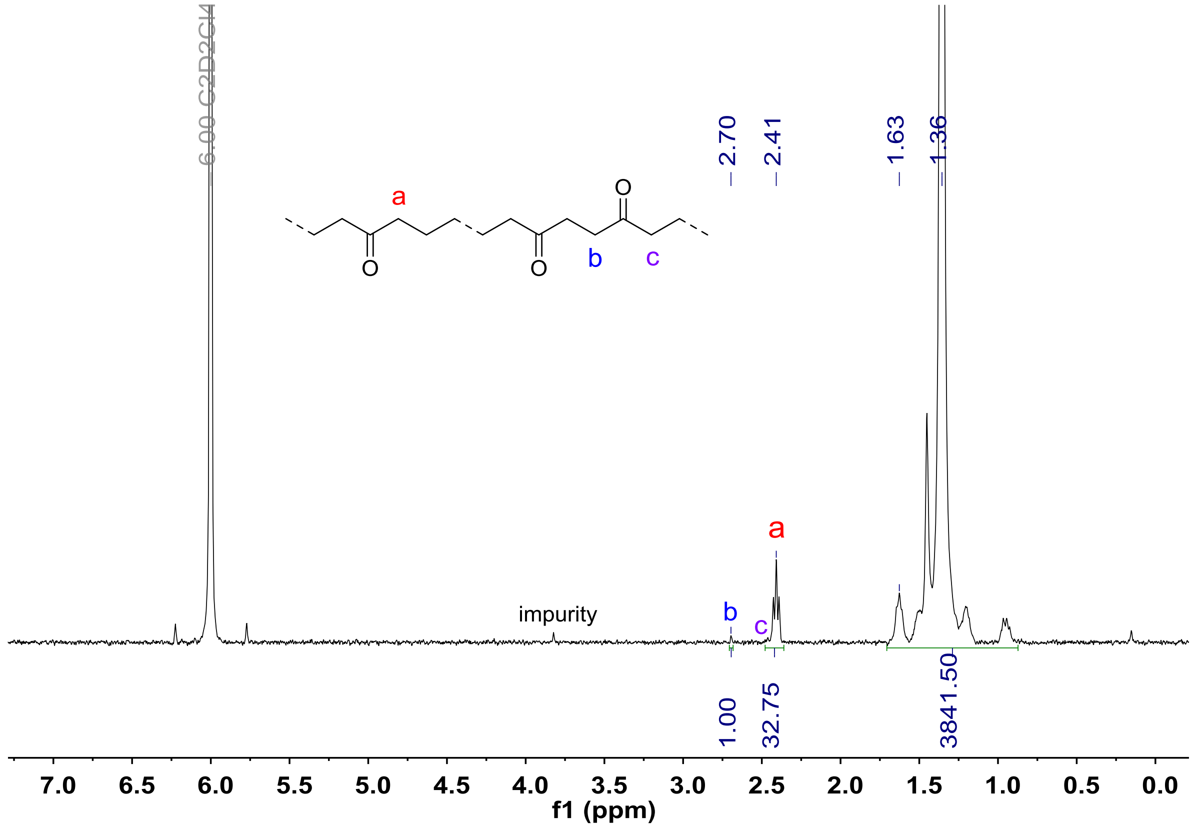

Figure S8. ^{1}H NMR spectrum (400 MHz, $C_2D_2Cl_4$, 110 ℃) of the polymer generated by complex **Pd4** from table 1, entry 4.

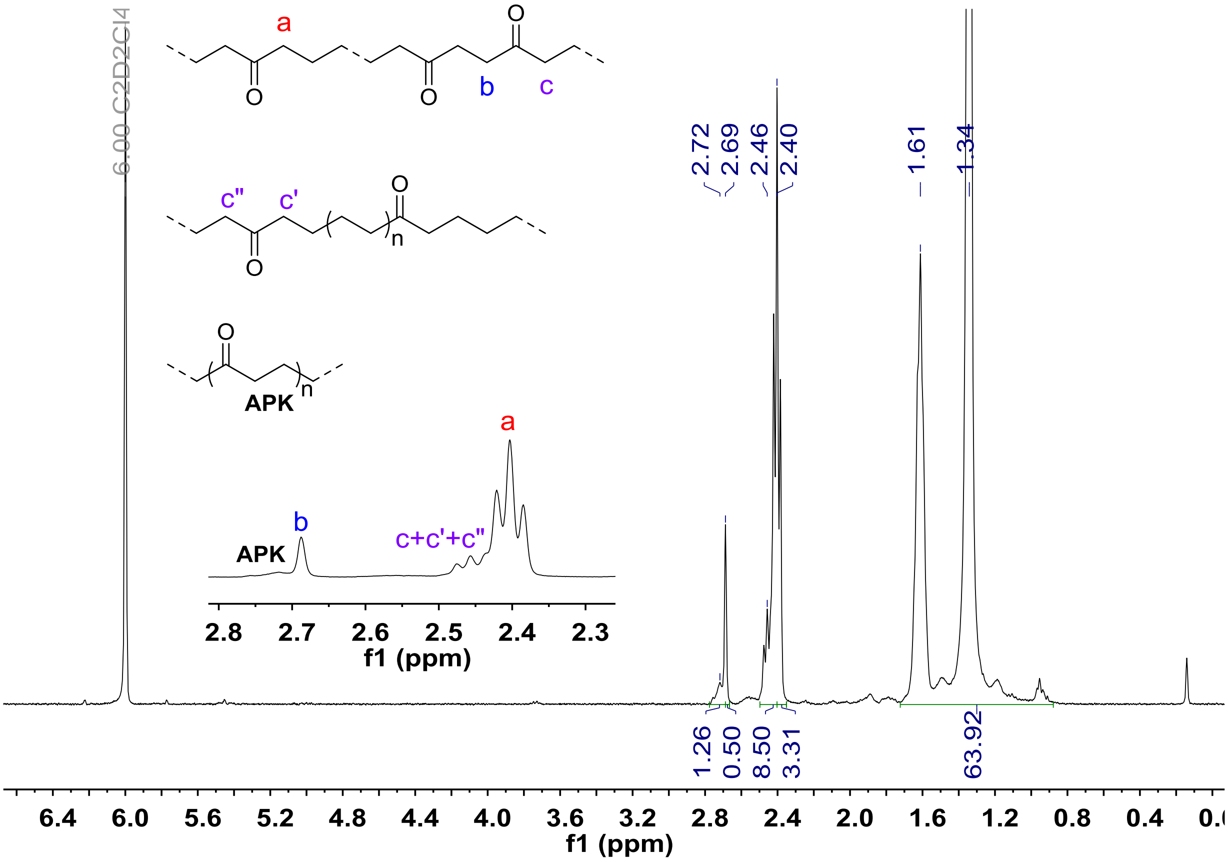

Figure S9. ^{1}H NMR spectrum (400 MHz, $C_2D_2Cl_4$, 110 ℃) of the polymer generated by complex **Pd2** from table 1, entry 6.

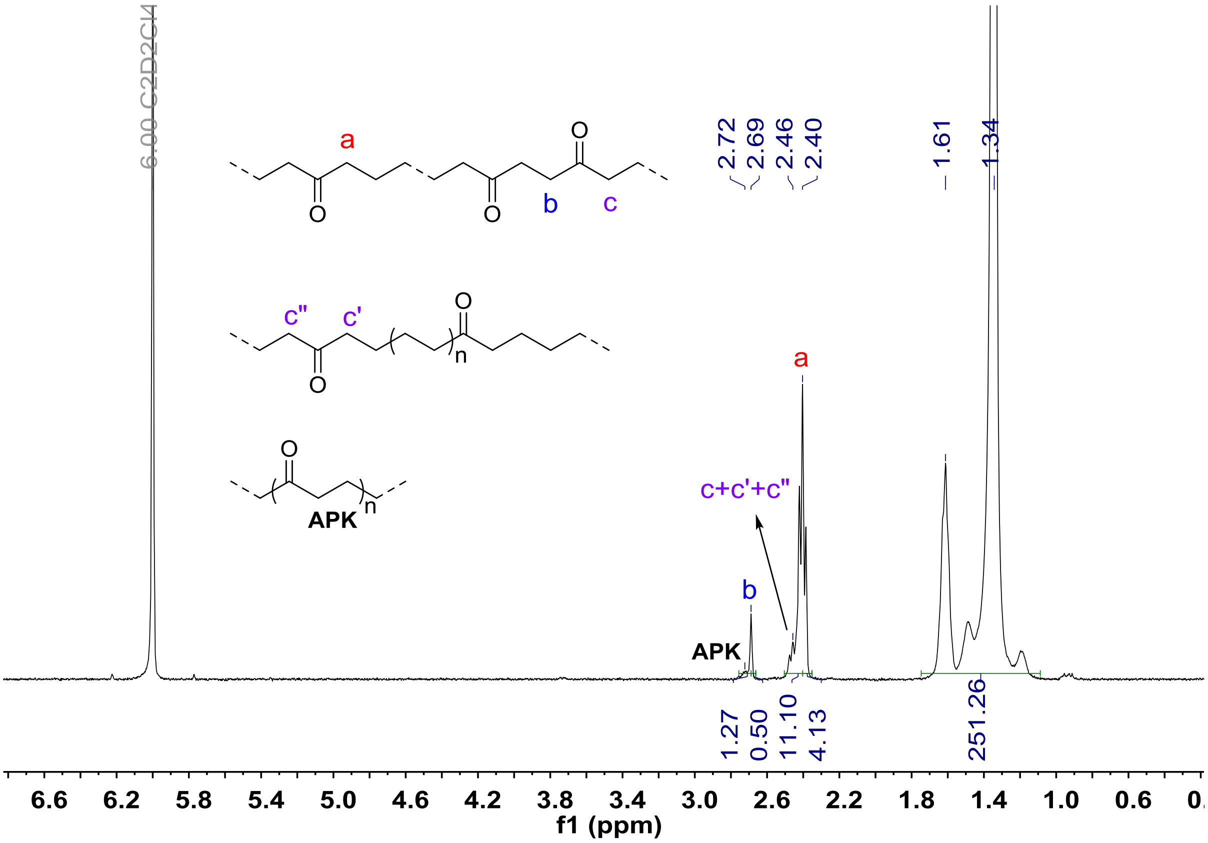

Figure S10. ^{1}H NMR spectrum (400 MHz, $C_2D_2Cl_4$, 110 ℃) of the polymer generated by complex **Pd3** from table 1, entry 7.

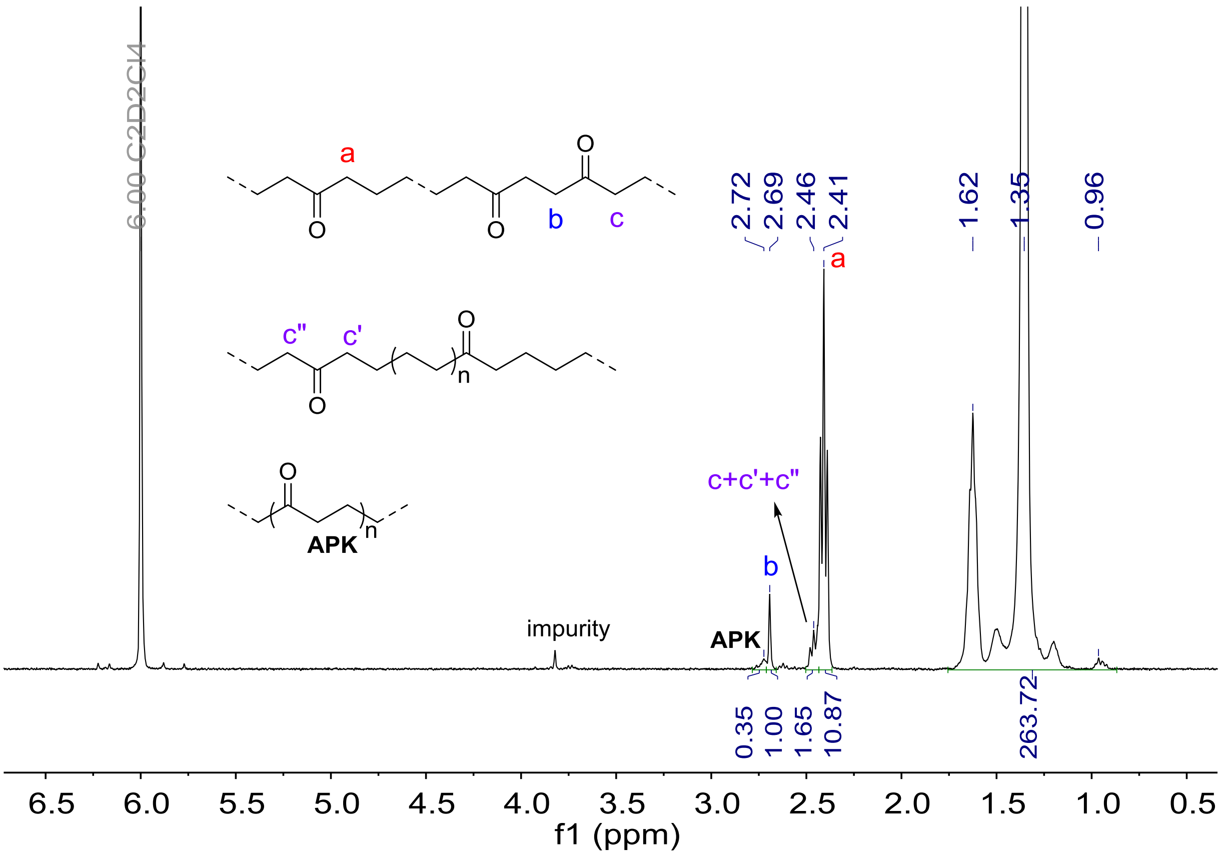

Figure S11. ^{1}H NMR spectrum (400 MHz, $C_2D_2Cl_4$, 110 ℃) of the polymer generated by complex **Pd4** from table 1, entry 8.

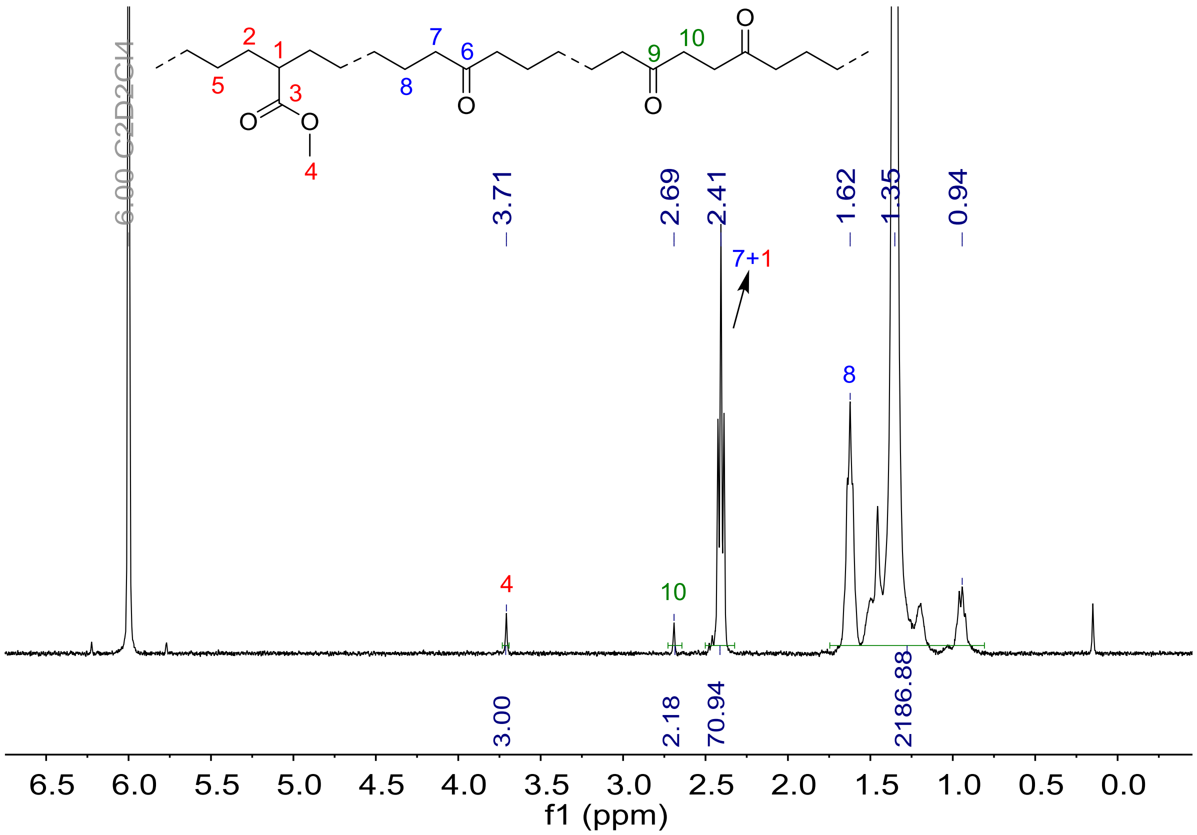

Figure S12. ^{1}H NMR spectrum (400 MHz, $C_2D_2Cl_4$, 110 ℃) of the polymer generated by complex **Pd3** from table 2, entry 1.

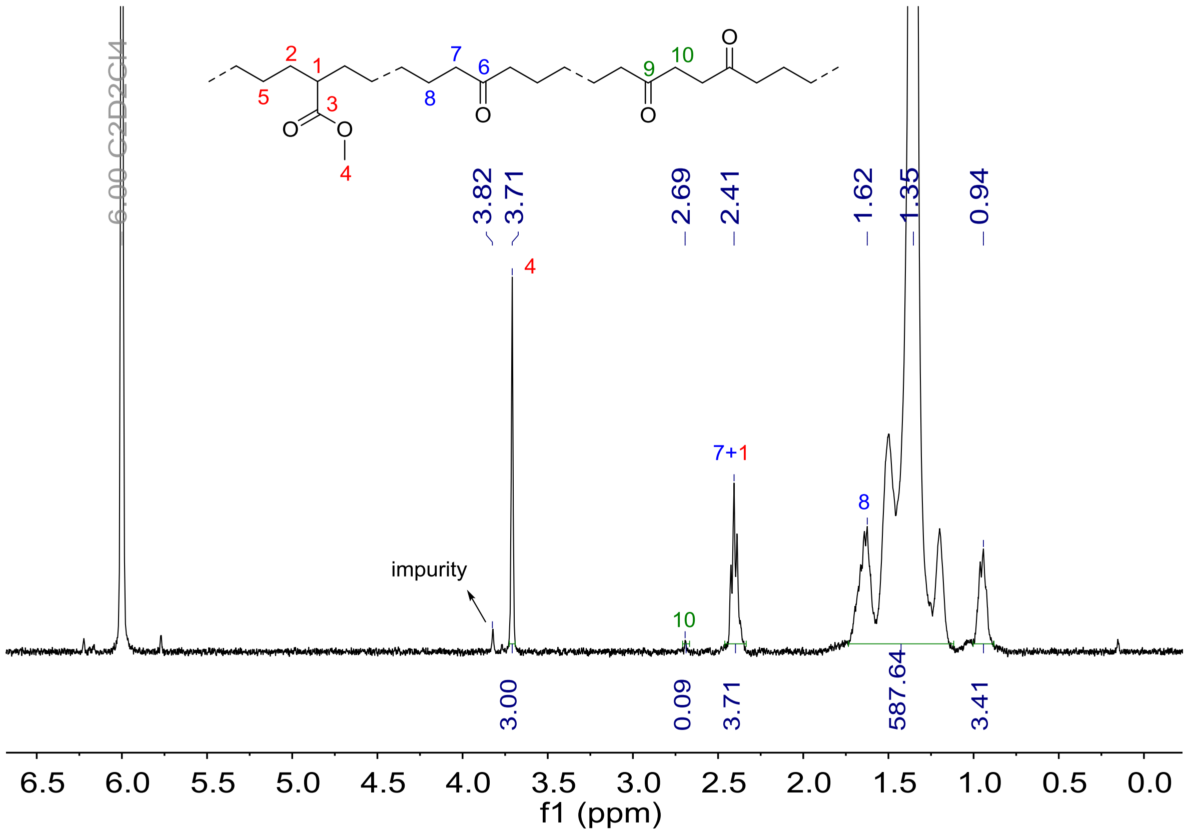

Figure S13. ^{1}H NMR spectrum (400 MHz, $C_2D_2Cl_4$, 110 ℃) of the polymer generated by complex **Pd4** from table 2, entry 2.

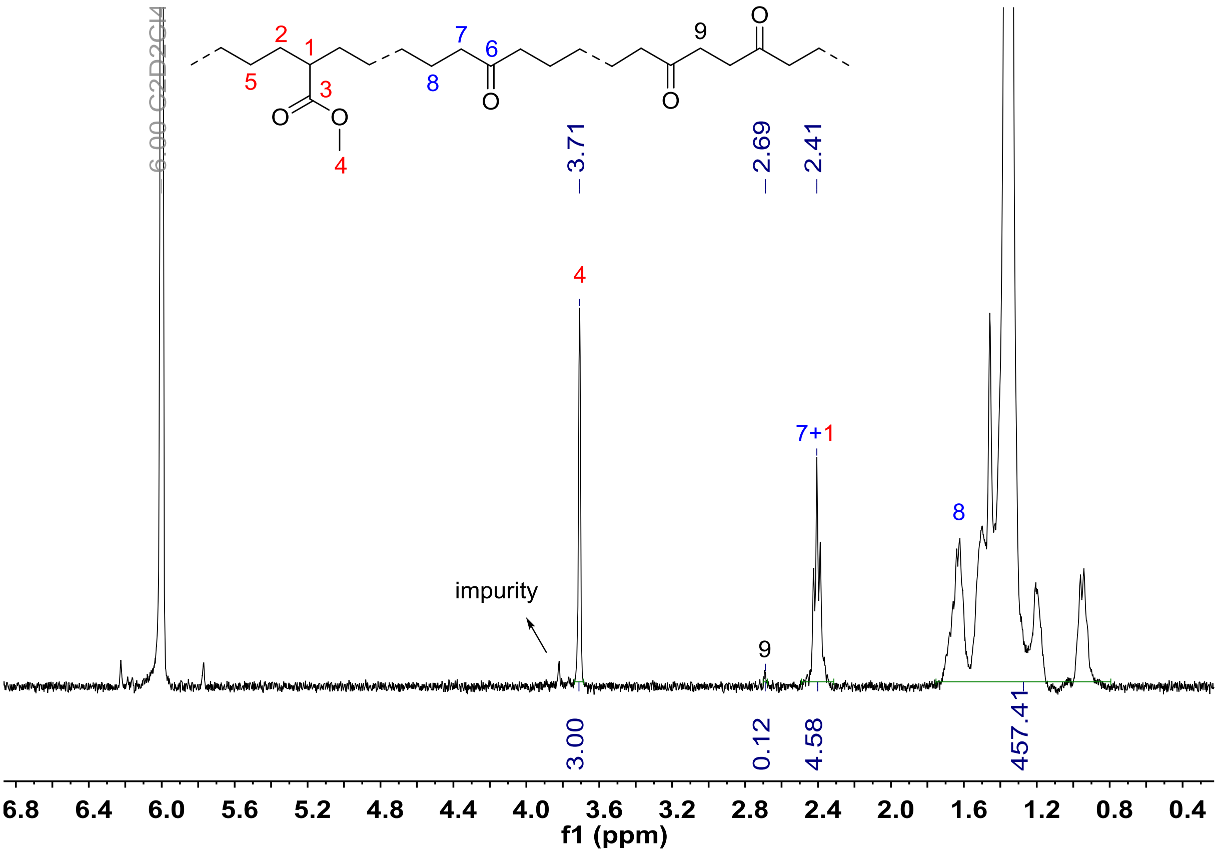

Figure S14. ^{1}H NMR spectrum (400 MHz, $C_2D_2Cl_4$, 110 ℃) of the polymer generated by complex **Pd4** from table 2, entry 3.

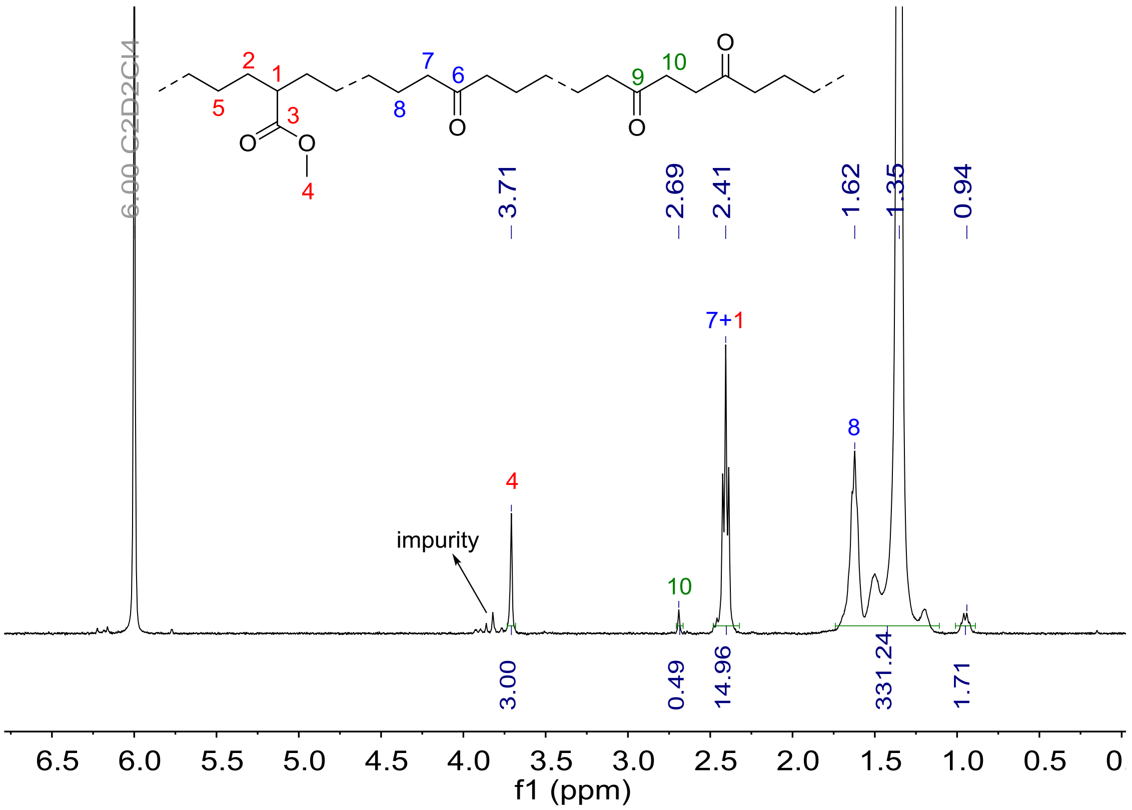

Figure S15. ^{1}H NMR spectrum (400 MHz, $C_2D_2Cl_4$, 110 ℃) of the polymer generated by complex **Pd4** from table 2, entry 4.

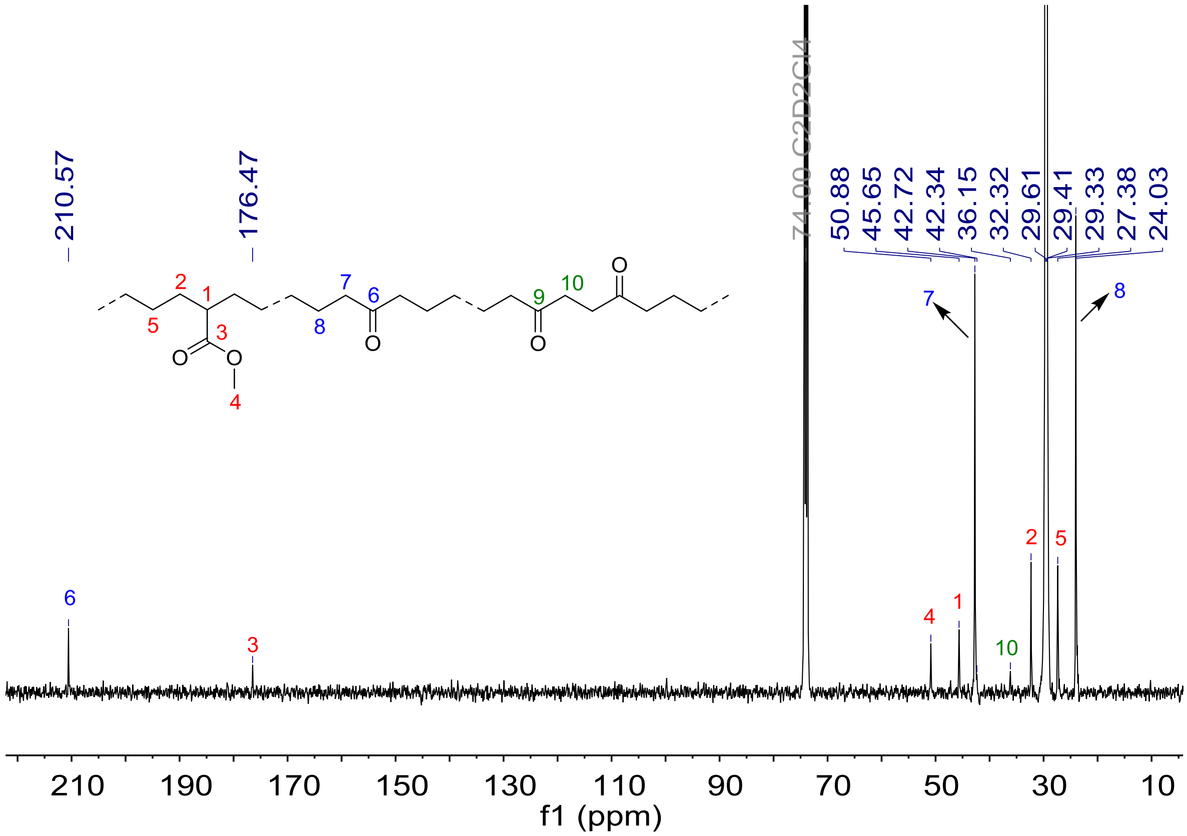

Figure S16. ^{13}C NMR spectrum (100 MHz, $C_2D_2Cl_4$, 110 ℃) of the polymer generated by complex **Pd4** from table 2, entry 4.

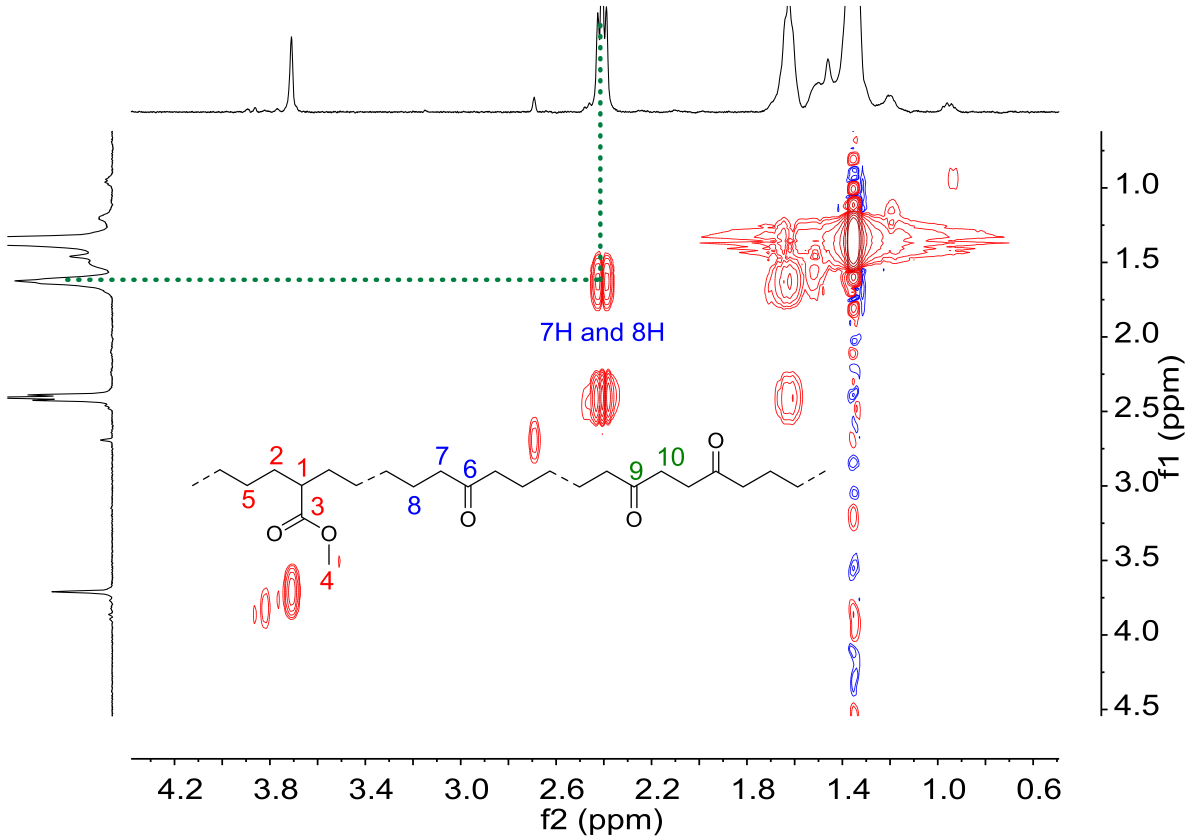

Figure S17. ^{1}H–^{1}H COSY NMR spectrum (400 MHz, $C_2D_2Cl_4$, 110 ℃) of the polymer generated by complex **Pd4** from table 2, entry 4.

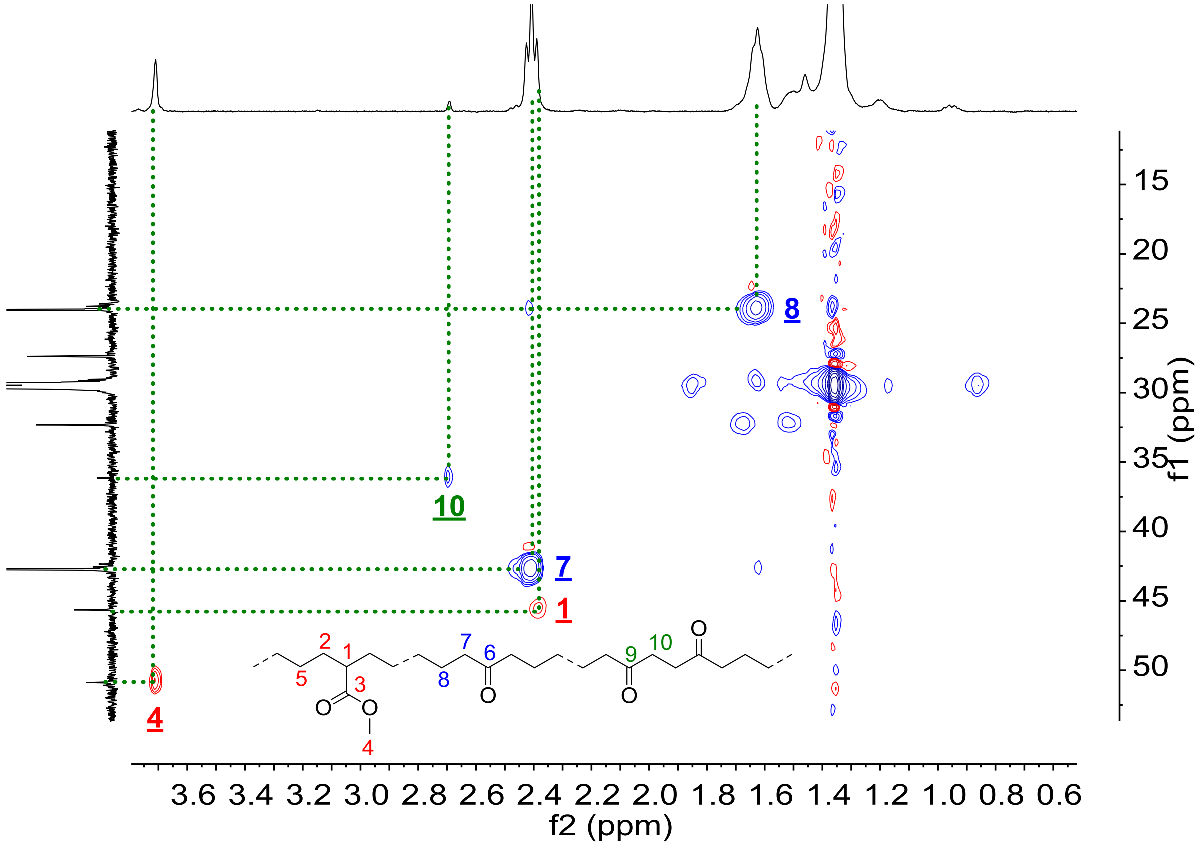

Figure S18. ^{1}H–^{13}C HSQC NMR spectrum (400 MHz, $C_2D_2Cl_4$, 110 ℃) of the polymer generated by complex **Pd4** from table 2, entry 4.

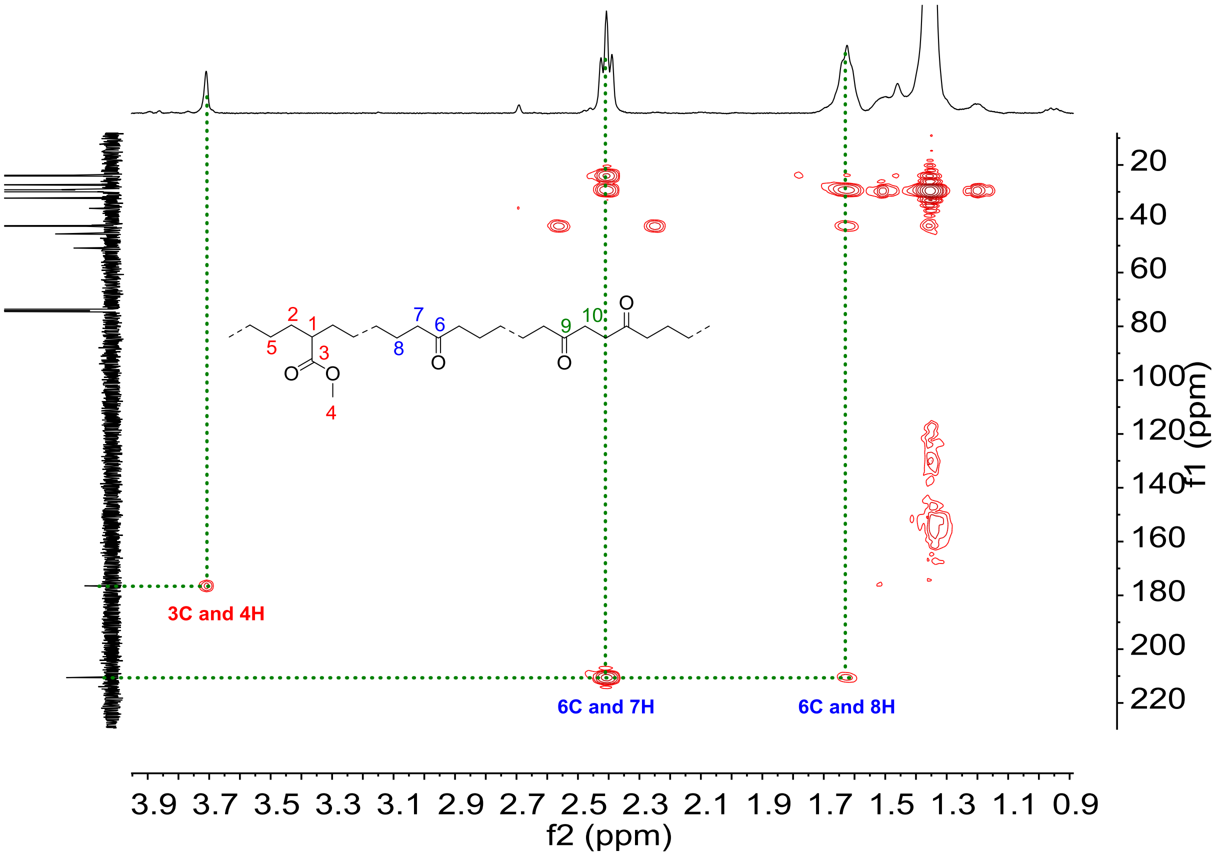

Figure S19. ^{1}H–^{13}C HMBC NMR spectrum (400 MHz, $C_2D_2Cl_4$, 110 ℃) of the polymer generated by complex **Pd4** from table 2, entry 4.

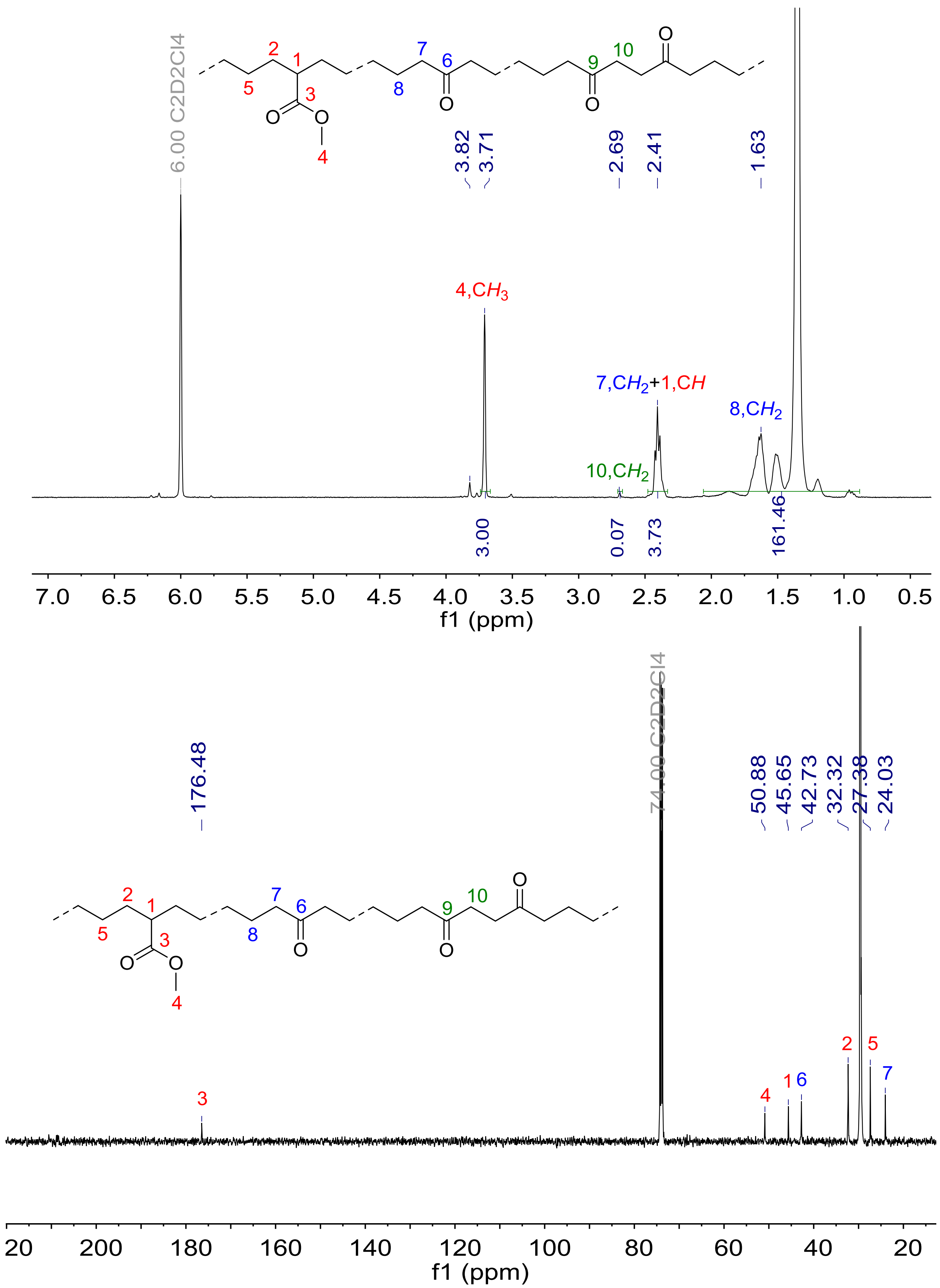

Figure S20. ^{1}H NMR spectrum (400 MHz, $C_2D_2Cl_4$, 110 ℃) and ^{13}C NMR spectrum (100MHz, $C_2D_2Cl_4$, 110 ℃) of the polymer generated by complex **Pd4** from table 2, entry 5.

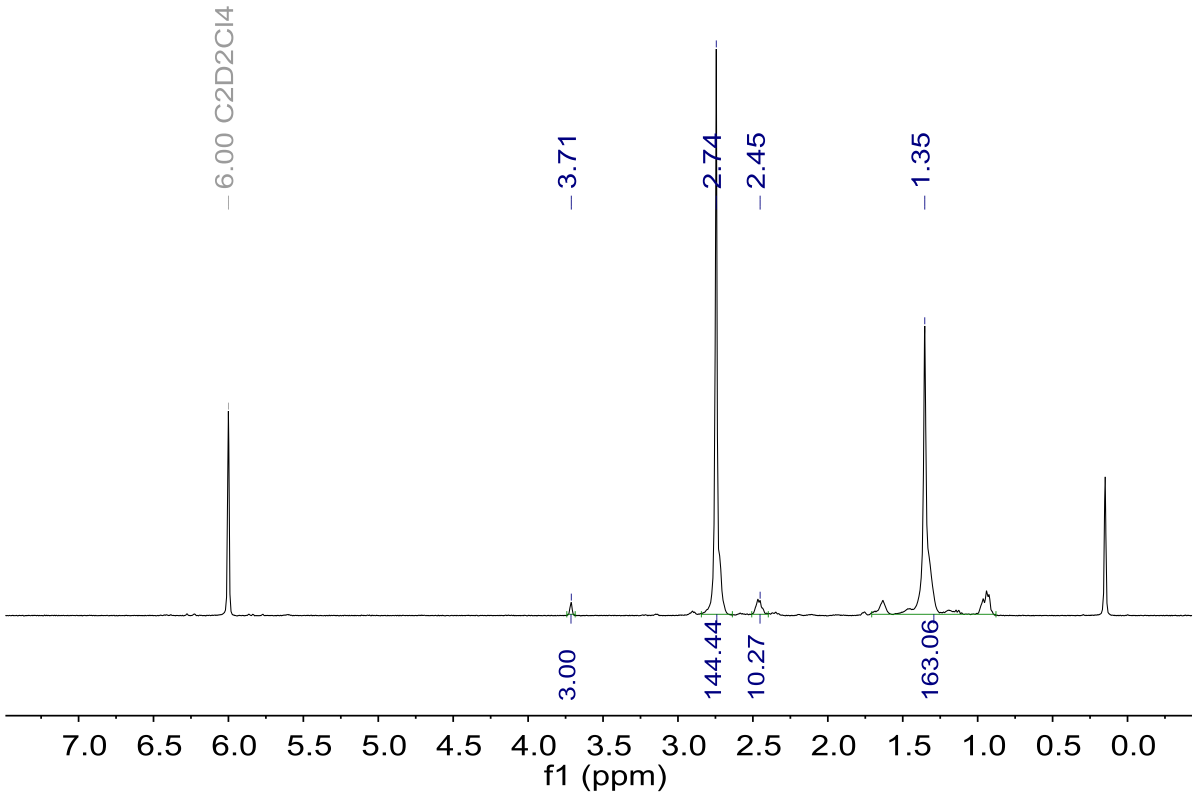

Figure S21. ^{1}H NMR spectrum (400 MHz, $C_2D_2Cl_4$, 110 ℃) of the polymer generated by complex **Pd5** from table 2, entry 6.

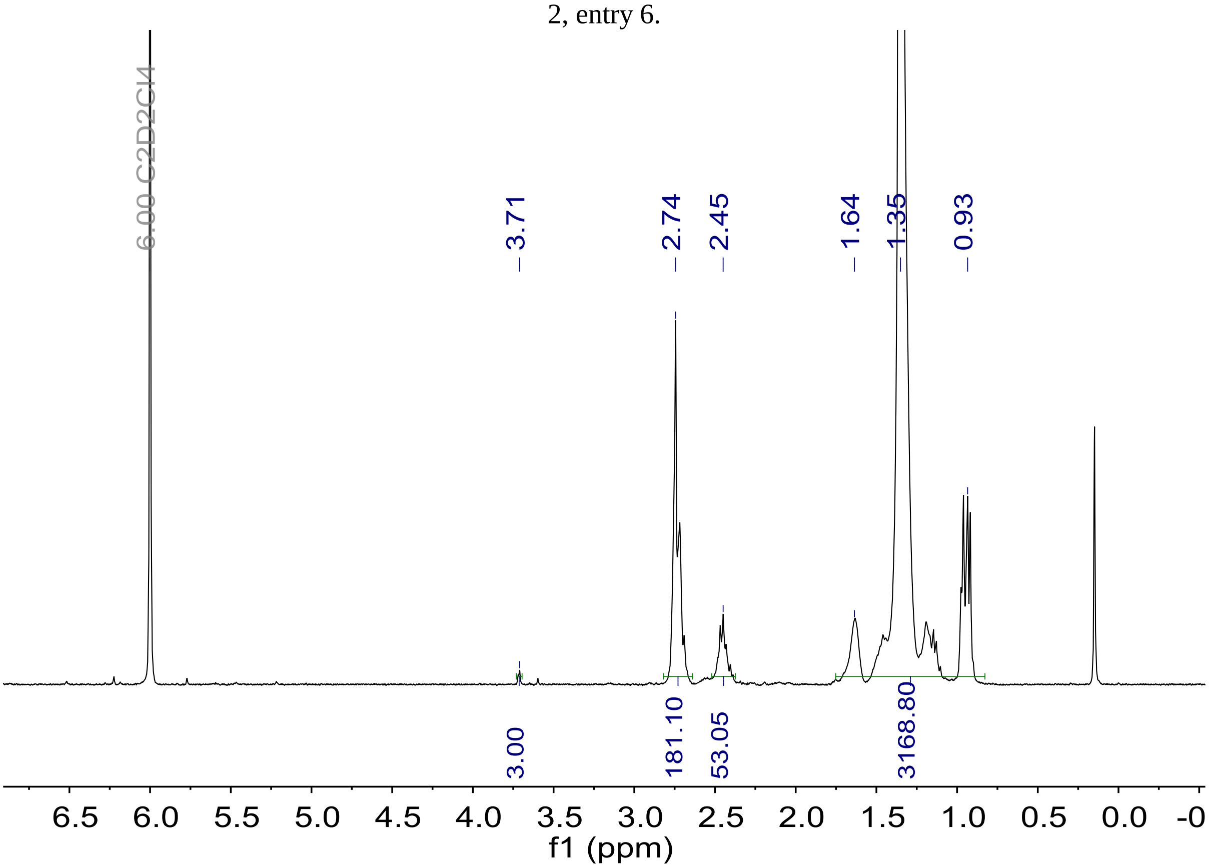

Figure S22. ^{1}H NMR spectrum (400 MHz, $C_2D_2Cl_4$, 110 ℃) of the polymer generated by complex **Pd6** from table 2, entry 7.

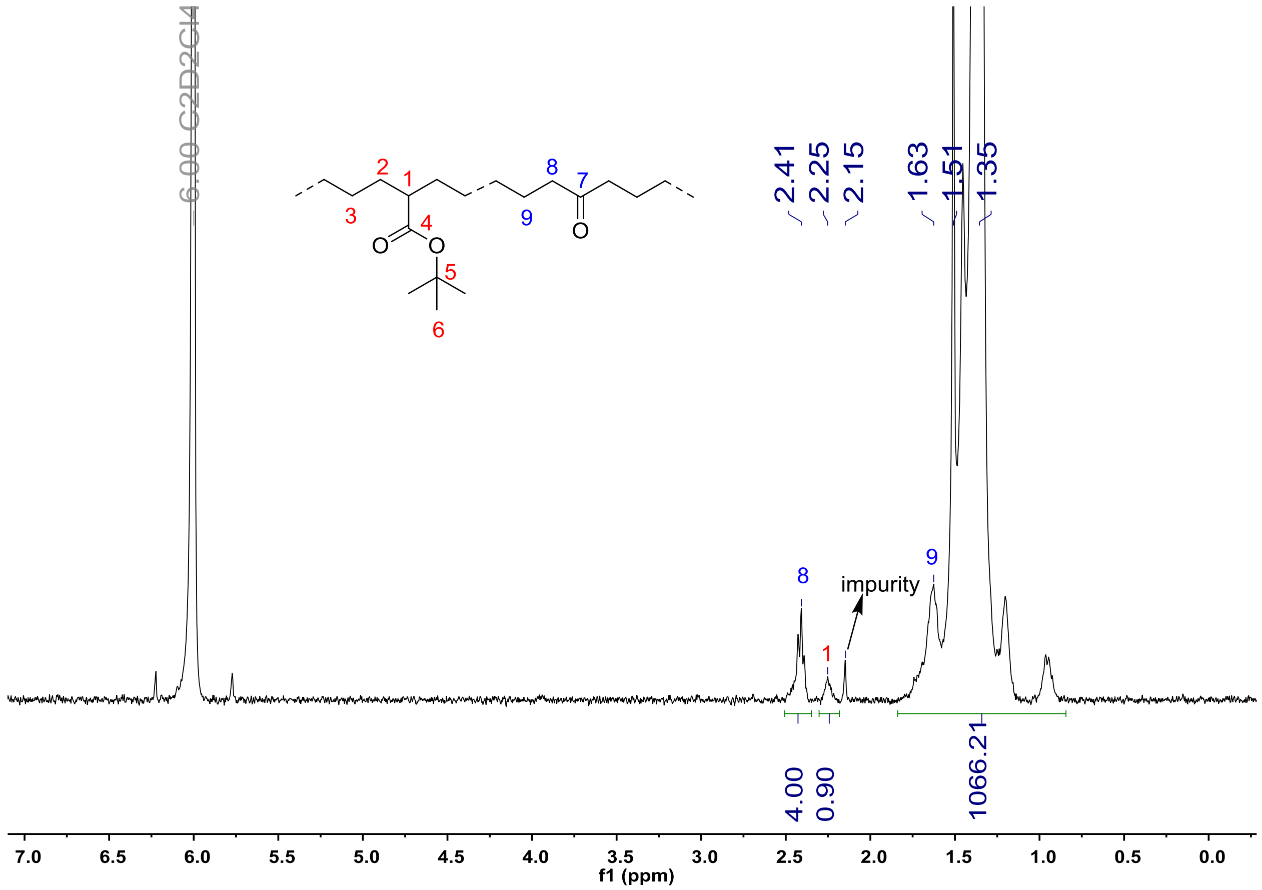

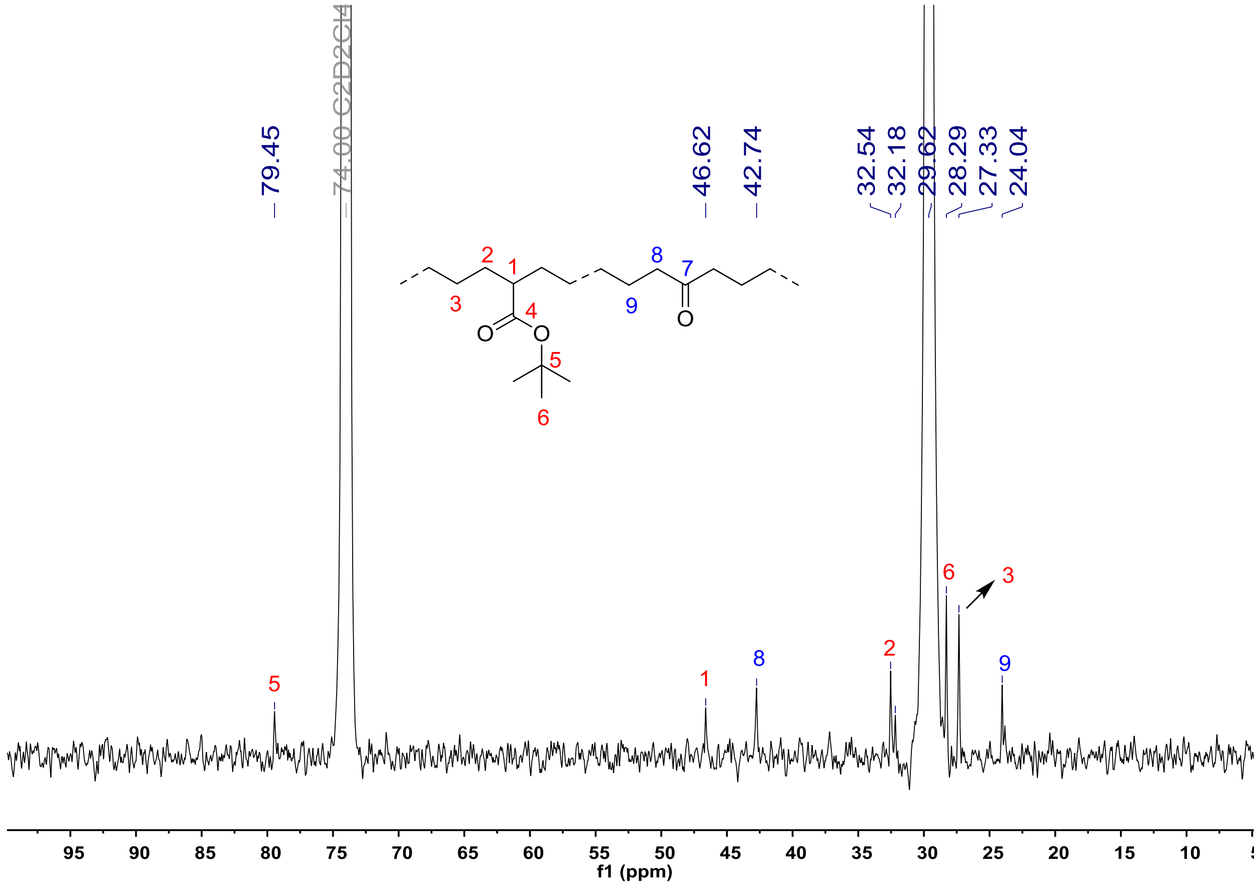

Figure S23. ^{1}H NMR spectrum (400 MHz, $C_2D_2Cl_4$, 110 ℃) and ^{13}C NMR spectrum (100MHz, $C_2D_2Cl_4$, 110 ℃) of the polymer generated by complex **Pd4** from table 2, entry 9.

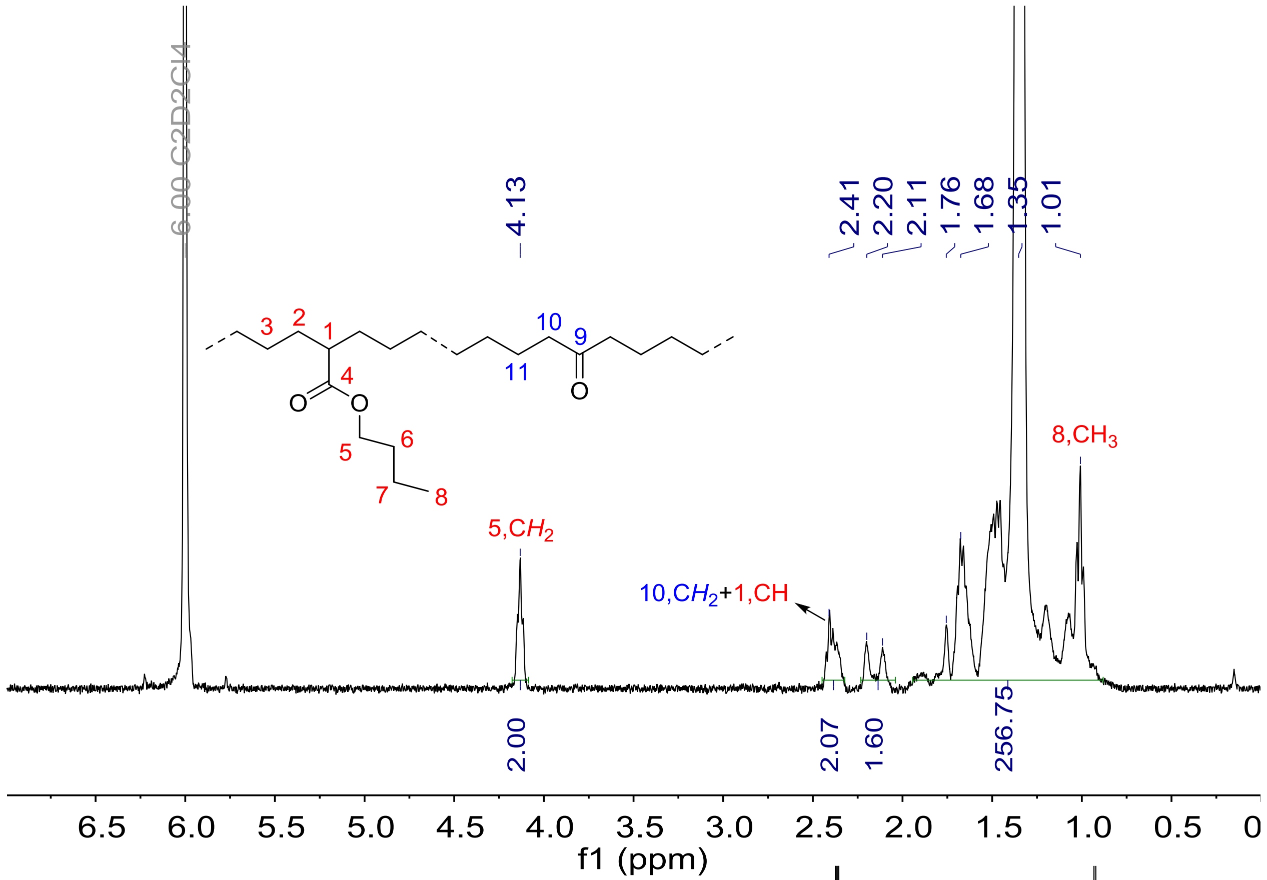

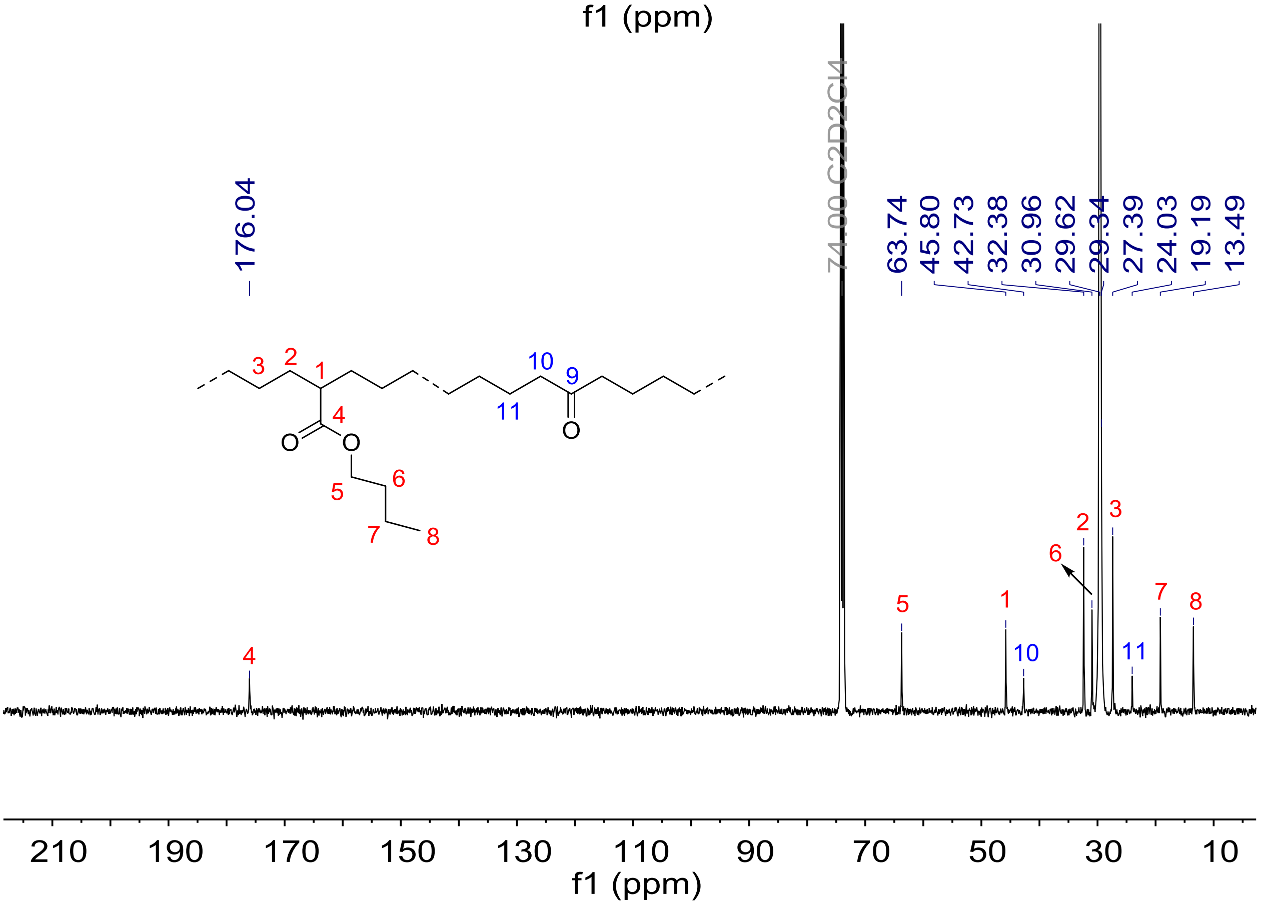

Figure S24. ^{1}H NMR spectrum (400 MHz, $C_2D_2Cl_4$, 110 ℃) and ^{13}C NMR spectrum (100MHz, $C_2D_2Cl_4$, 110 ℃) of the polymer generated by complex **Pd4** from table 2, entry 10.

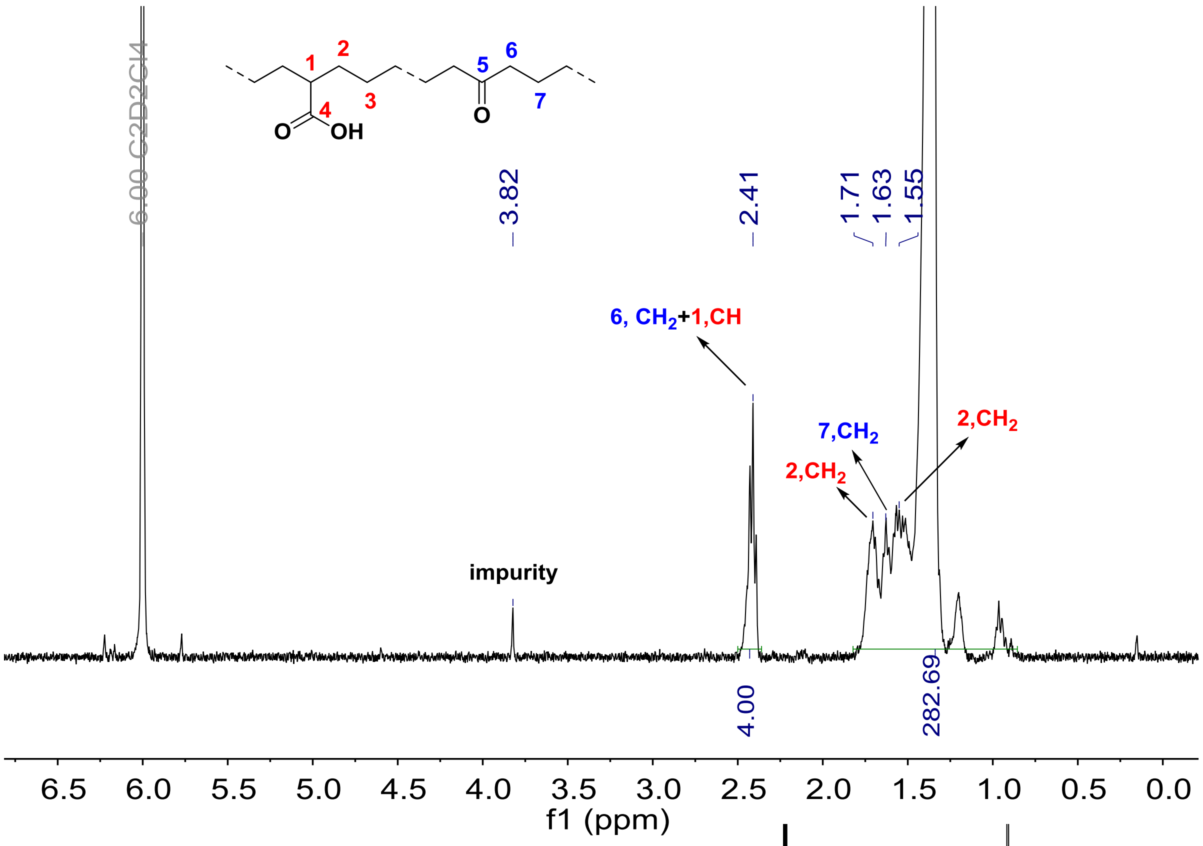

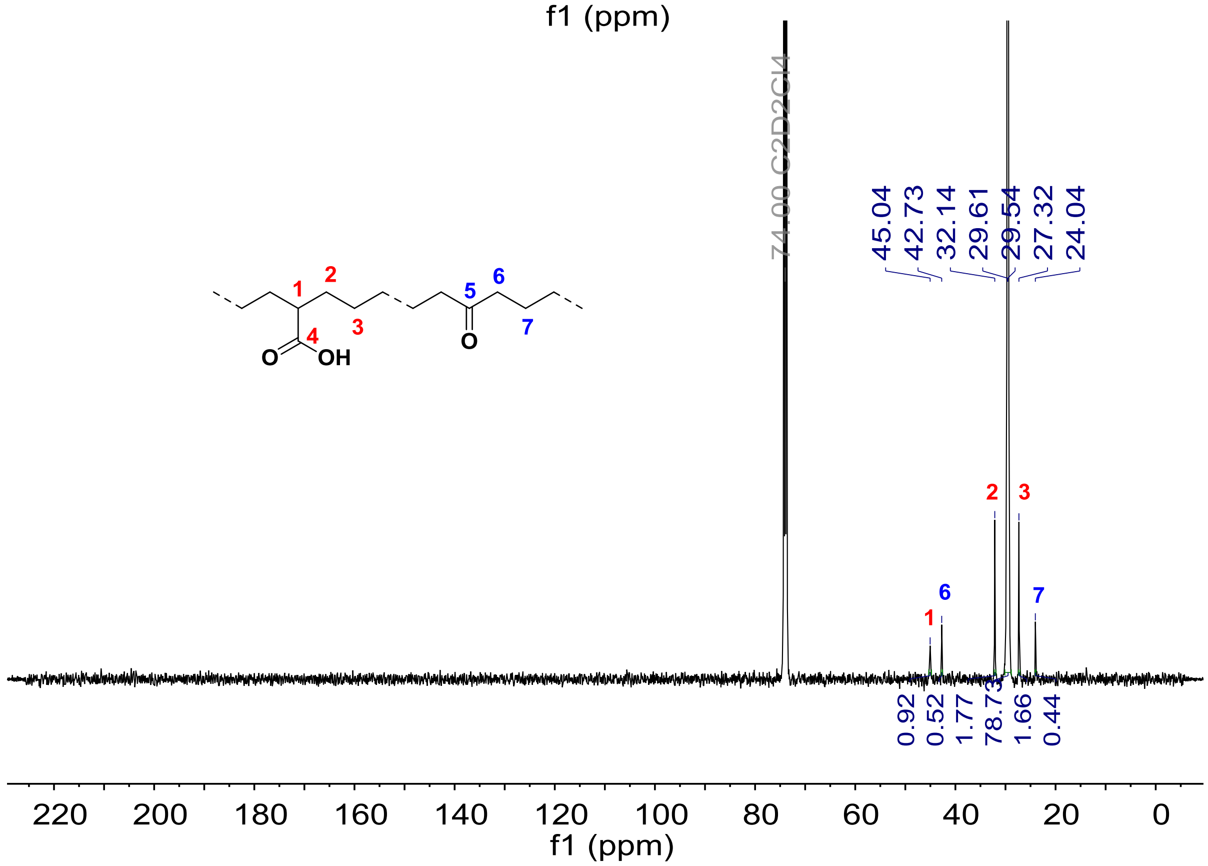

Figure S25. ^{1}H NMR spectrum (400 MHz, $C_2D_2Cl_4$, 110 ℃) and ^{13}C NMR spectrum (100MHz, $C_2D_2Cl_4$, 110 ℃) of the polymer generated by complex **Pd4** from table 2, entry 11.

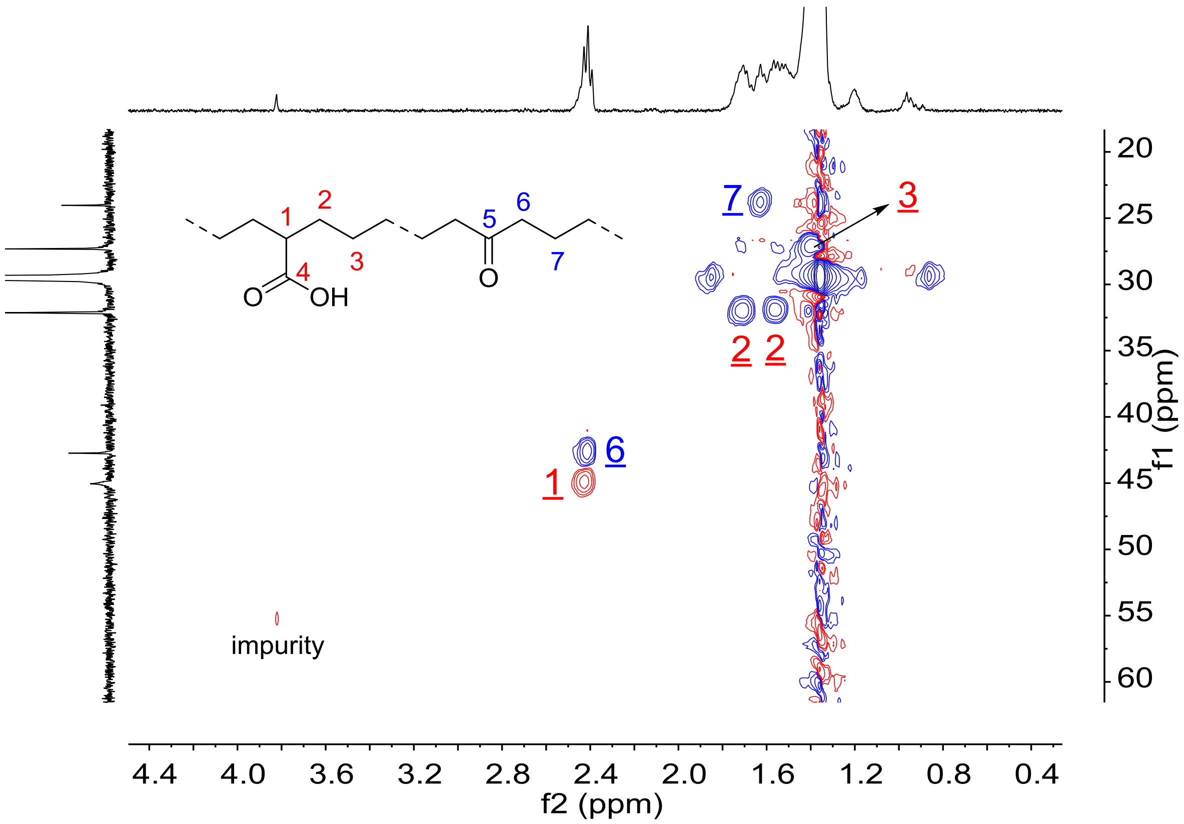

Figure S26. ^1H–^{13}C HSQC NMR spectrum (400 MHz, $C_2D_2Cl_4$, 110 ℃) of the polymer generated by complex **Pd4** from table 2, entry 11.

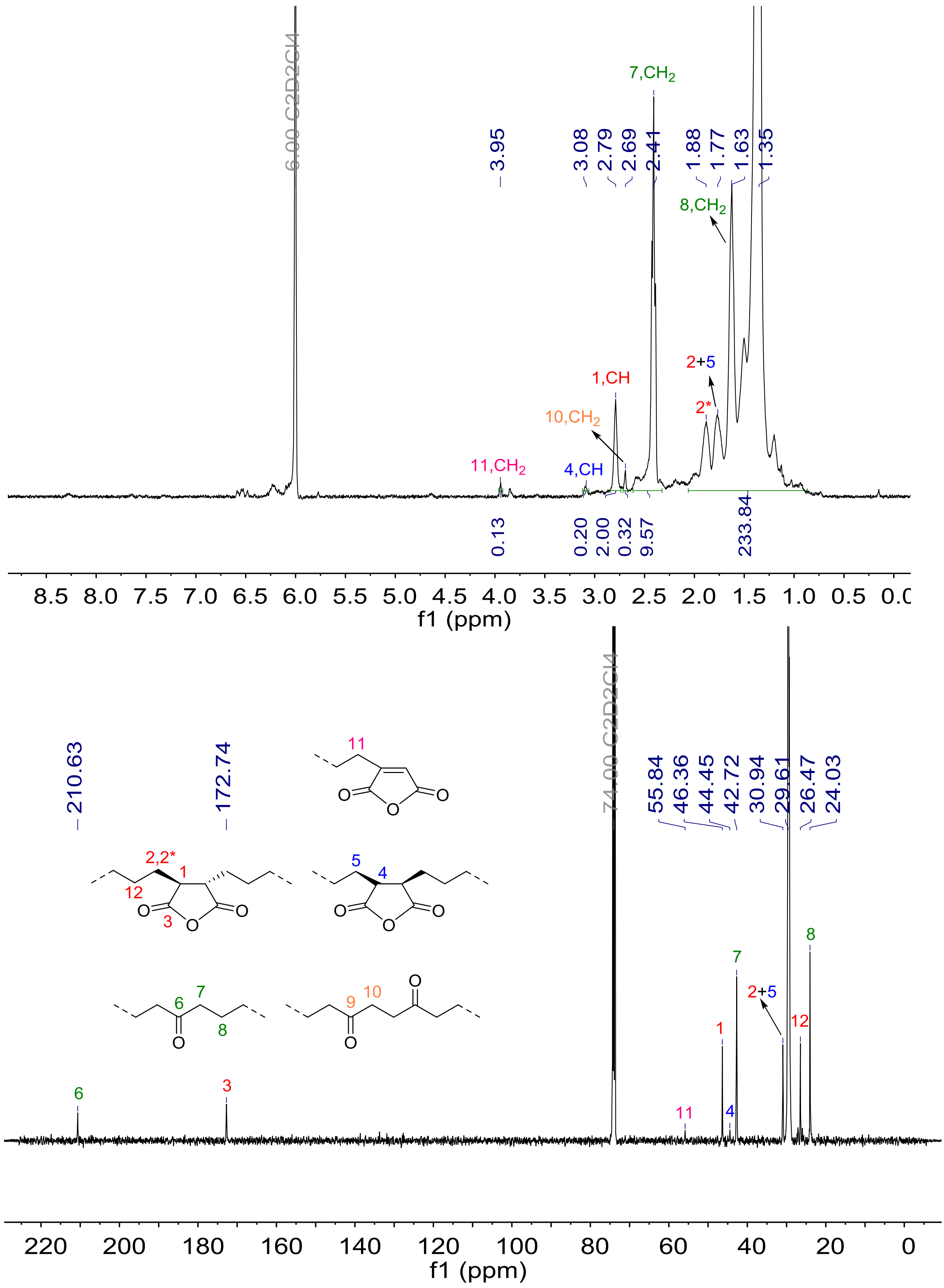

Figure S27. ^{1}H NMR spectrum (400 MHz, $C_2D_2Cl_4$, 110 ℃) and ^{13}C NMR spectrum (100MHz, $C_2D_2Cl_4$, 110 ℃) of the polymer generated by complex **Pd4** from table 2, entry 12.

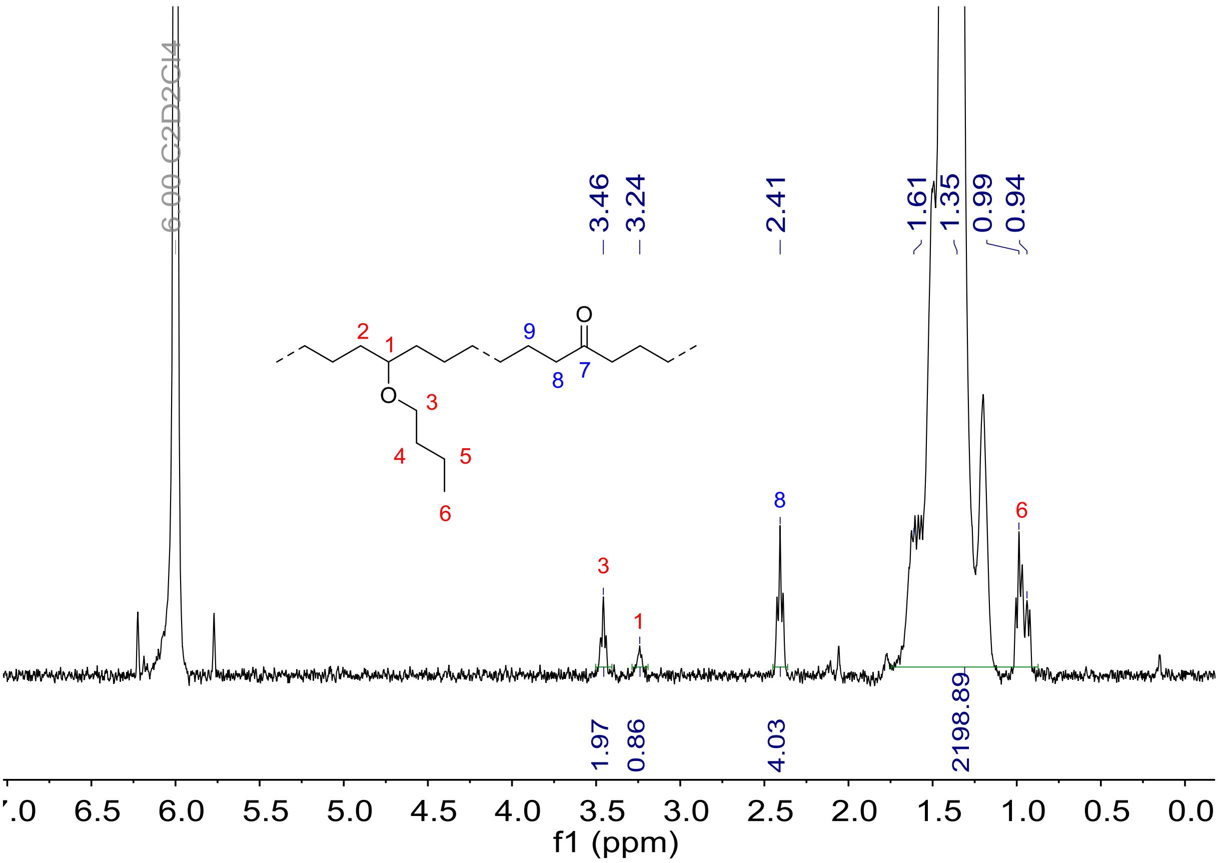

Figure S28. ^{1}H NMR spectrum (400 MHz, $C_2D_2Cl_4$, 110 ℃) of the polymer generated by complex **Pd4** from table 2, entry 13.

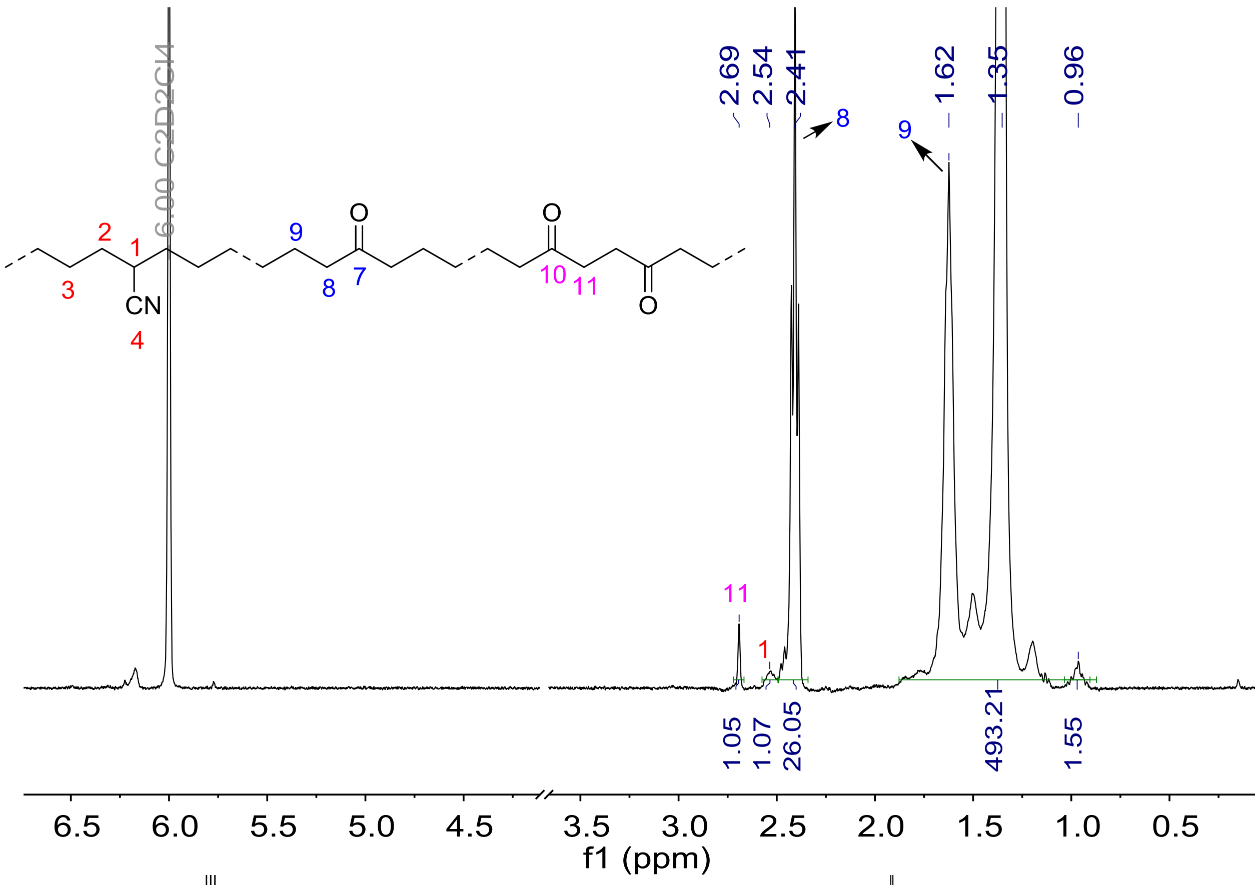

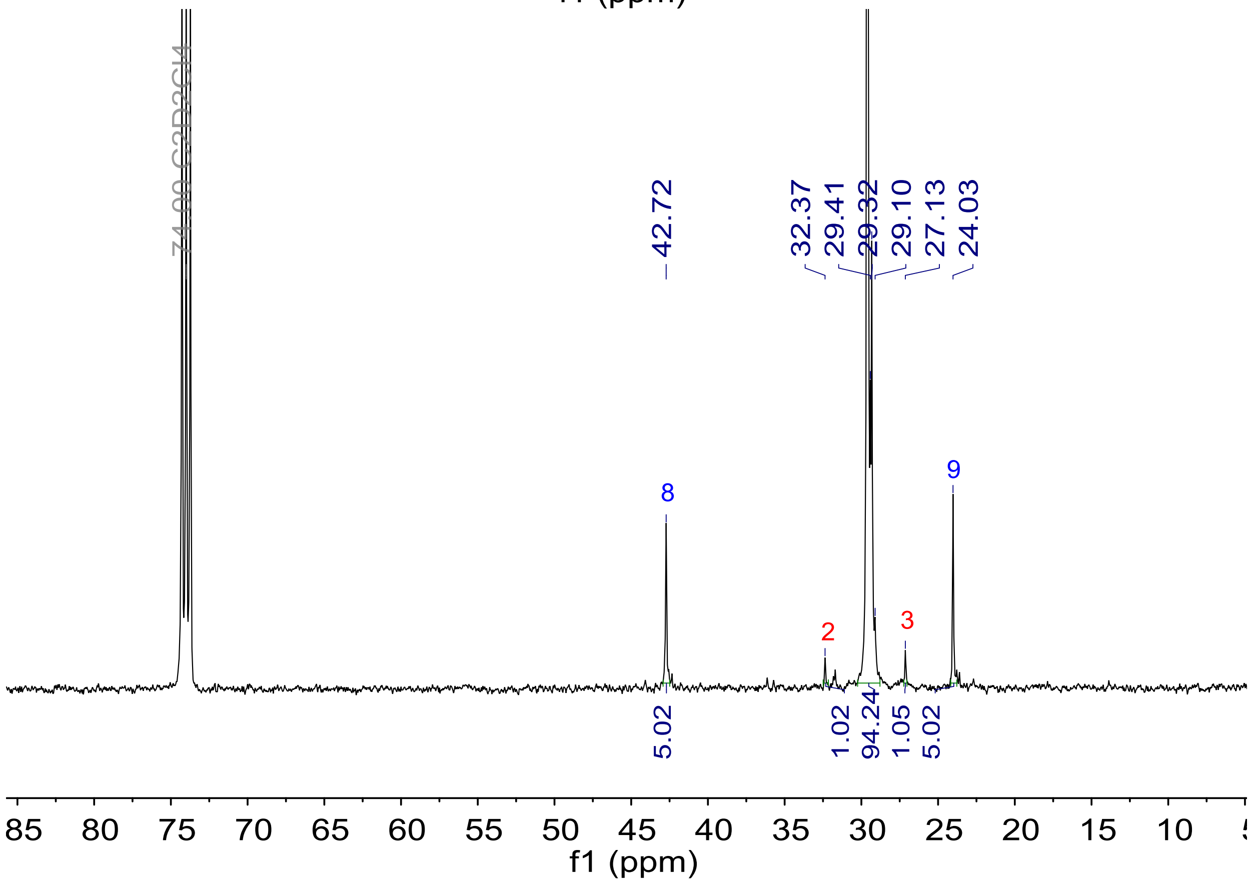

Figure S29. ^{1}H NMR spectrum (400 MHz, $C_2D_2Cl_4$, 110 ℃) and ^{13}C NMR spectrum (100MHz, $C_2D_2Cl_4$, 110 ℃) of the polymer generated by complex **Pd4** from table 2, entry 14.

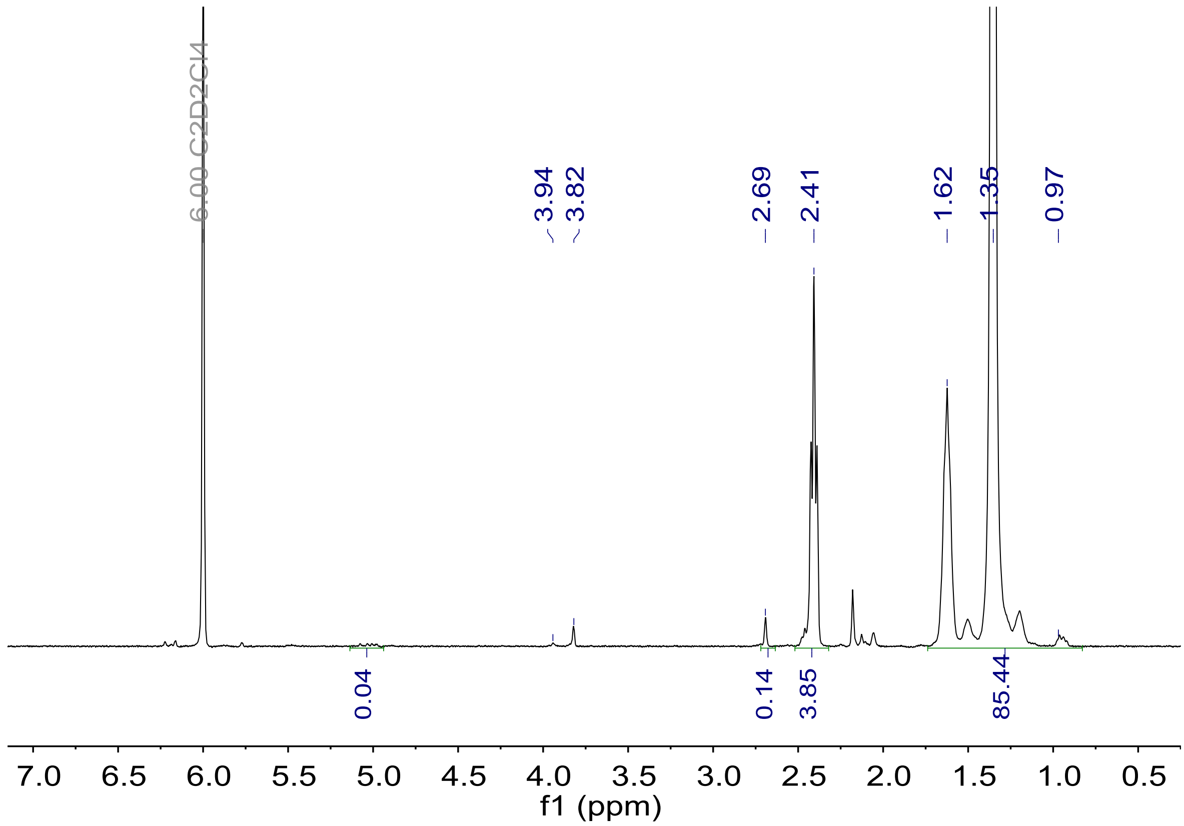

Figure S30. ^{1}H NMR spectrum (400 MHz, $C_2D_2Cl_4$, 110 ℃) of the polymer generated by complex **Pd4** from table 2, entry 15.

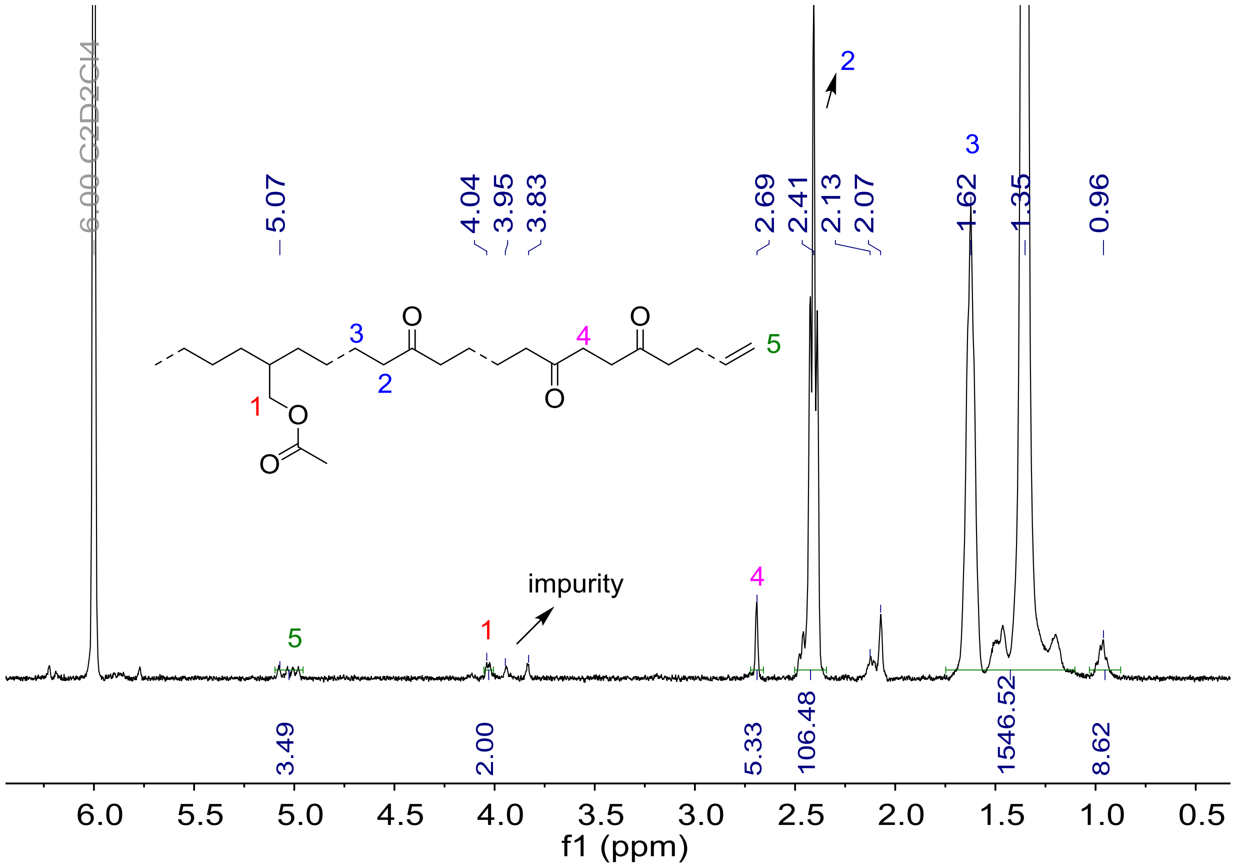

Figure S31. ^{1}H NMR spectrum (400 MHz, $C_2D_2Cl_4$, 110 ℃) of the polymer generated by complex **Pd4** from table 2, entry 16.

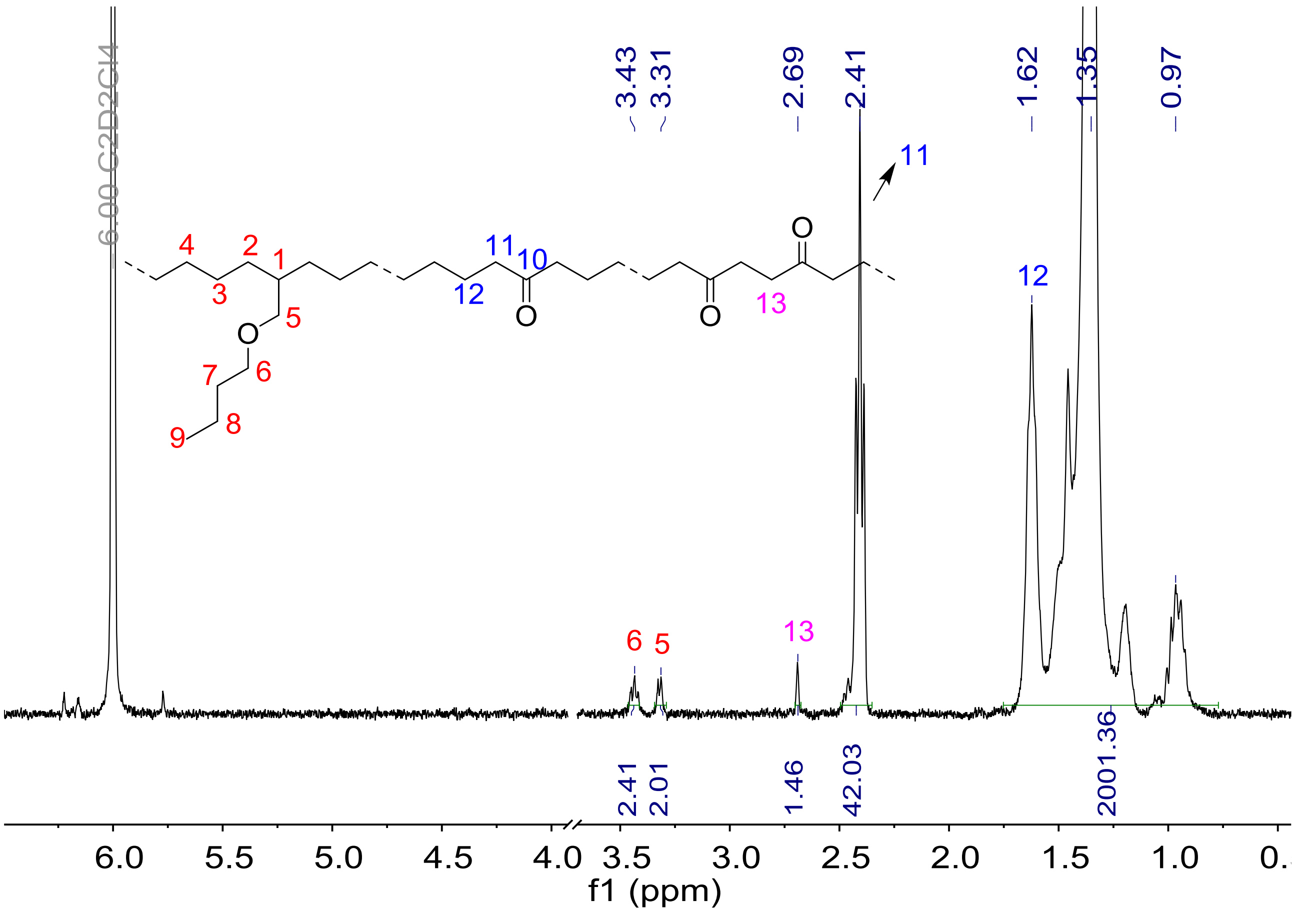

Figure S32. ^{1}H NMR spectrum (400 MHz, $C_2D_2Cl_4$, 110 ℃) of the polymer generated by complex **Pd4** from table 2, entry 17.

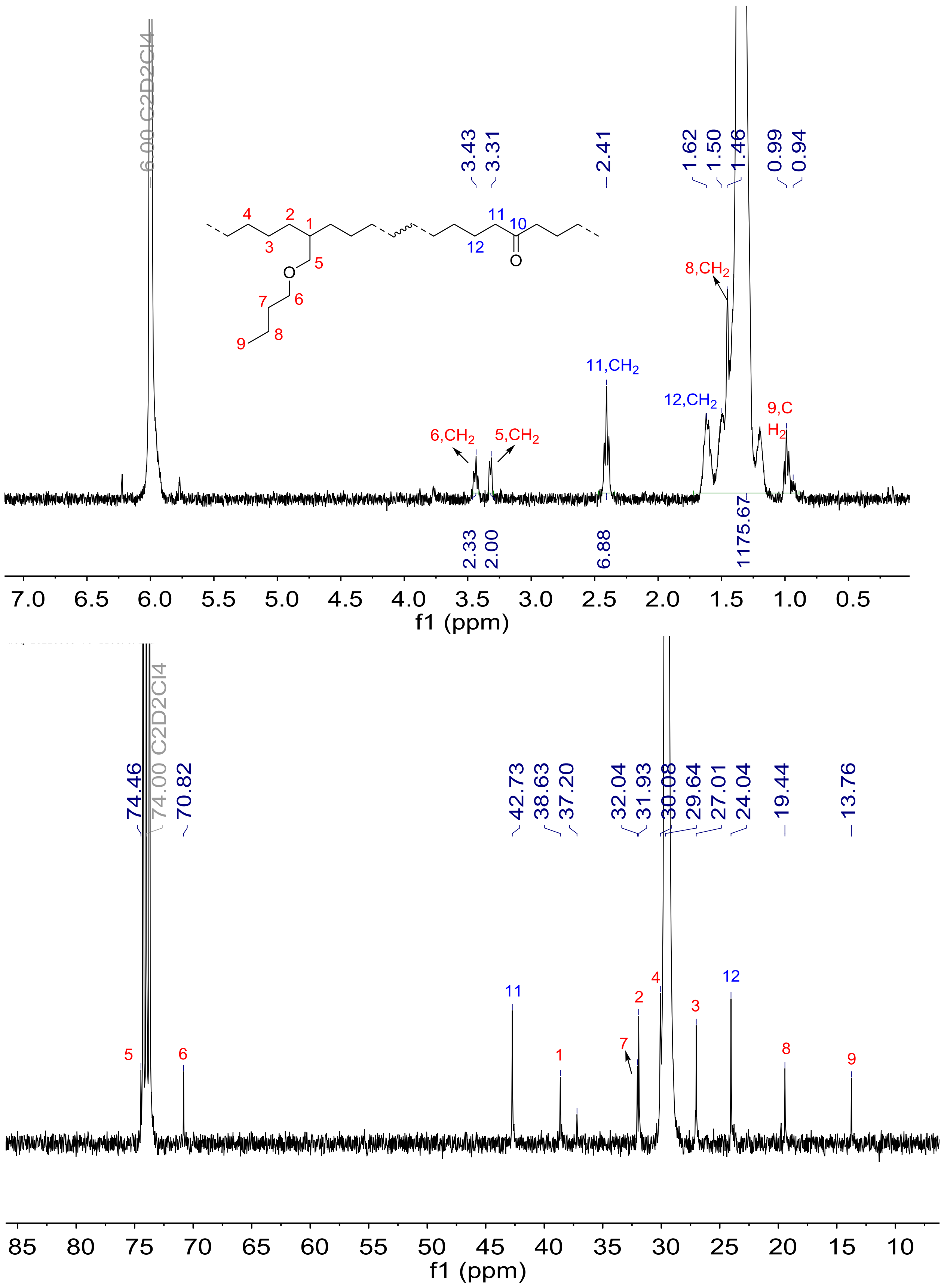

Figure S33. ^{1}H NMR spectrum (400 MHz, $C_2D_2Cl_4$, 110 ℃) and ^{13}C NMR spectrum (100MHz, $C_2D_2Cl_4$, 110 ℃) of the polymer generated by complex **Pd4** from table 2, entry 18.

4. GPC Curves of Polymers

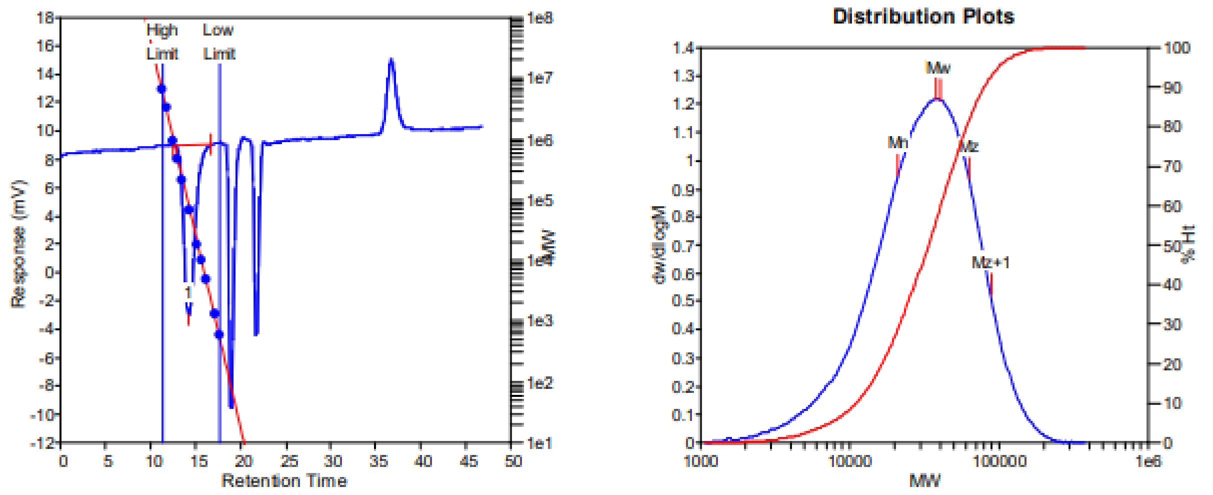

MW Averages

Peak No	Mp	Mn	Mw	Mz	Mz+1	Mv	PD
1	37377	21260	40389	62844	88113	37690	1.89976

Processed Peaks

Peak No	Name	Start RT (mins)	Max RT (mins)	End RT (mins)	Pk Height (mV)	% Height	Area (mV.secs)	% Area
1		12.53	14.17	16.67	-11.9291	0	954.499	100

Figure S34. GPC trace of the polymer from table 1, entry 1.

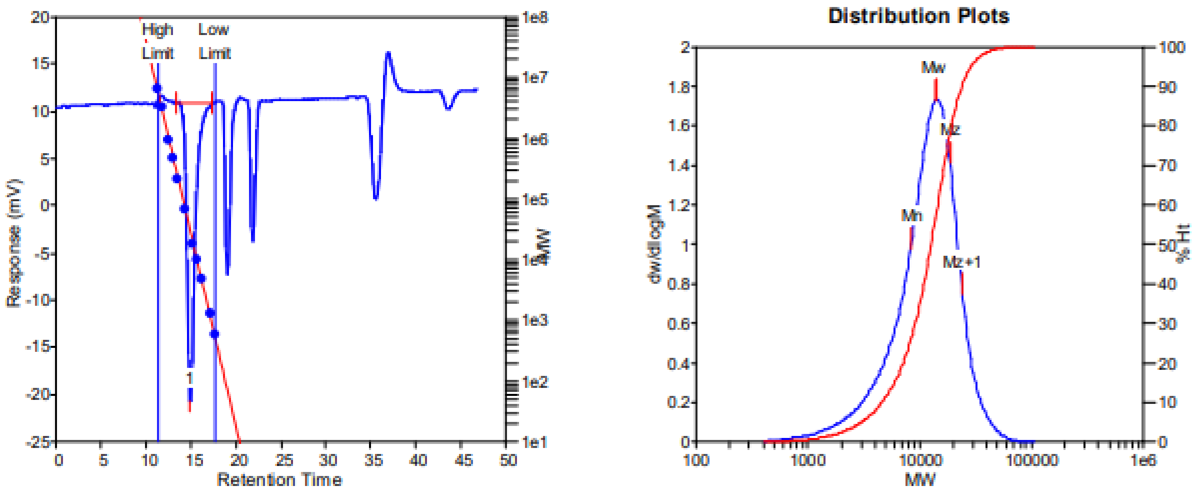

MW Averages

Peak No	Mp	Mn	Mw	Mz	Mz+1	Mv	PD
1	14170	8314	13714	18666	24265	13072	1.64951

Processed Peaks

Peak No	Name	Start RT (mins)	Max RT (mins)	End RT (mins)	Pk Height (mV)	% Height	Area (mV.secs)	% Area
1		13.42	14.85	17.37	-31.6929	100	1778.42	100

Figure S35. GPC trace of the polymer from table 1, entry 2.

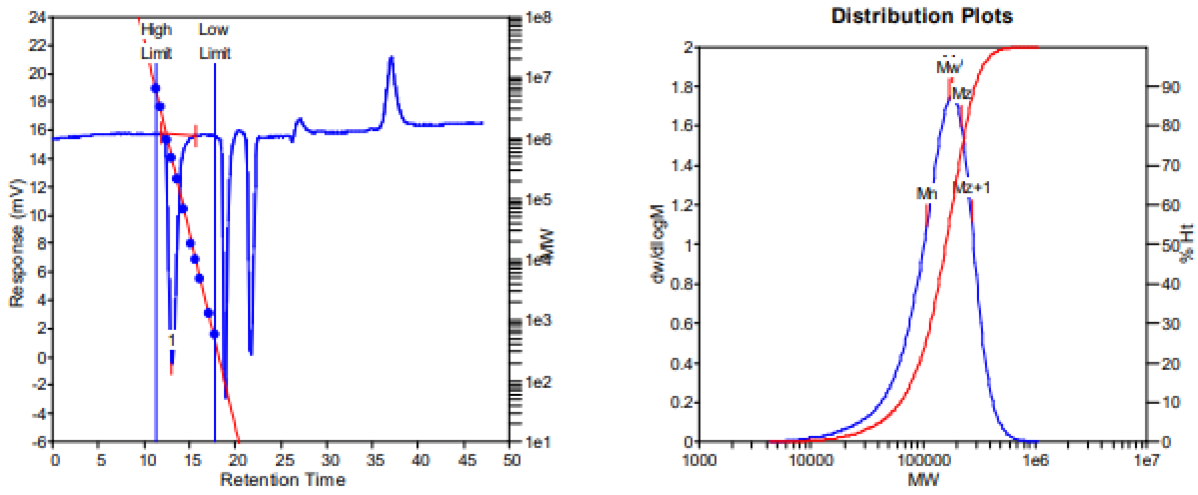

MW Averages

Peak No	Mp	Mn	Mw	Mz	Mz+1	Mv	PD
1	182360	108123	169919	221179	272678	162921	1.57153

Processed Peaks

Peak No	Name	Start RT (mins)	Max RT (mins)	End RT (mins)	Pk Height (mV)	% Height	Area (mV.secs)	% Area
1		11.80	13.05	15.68	-16.1848	100	898.313	100

Figure S36. GPC trace of the polymer from table 1, entry 3.

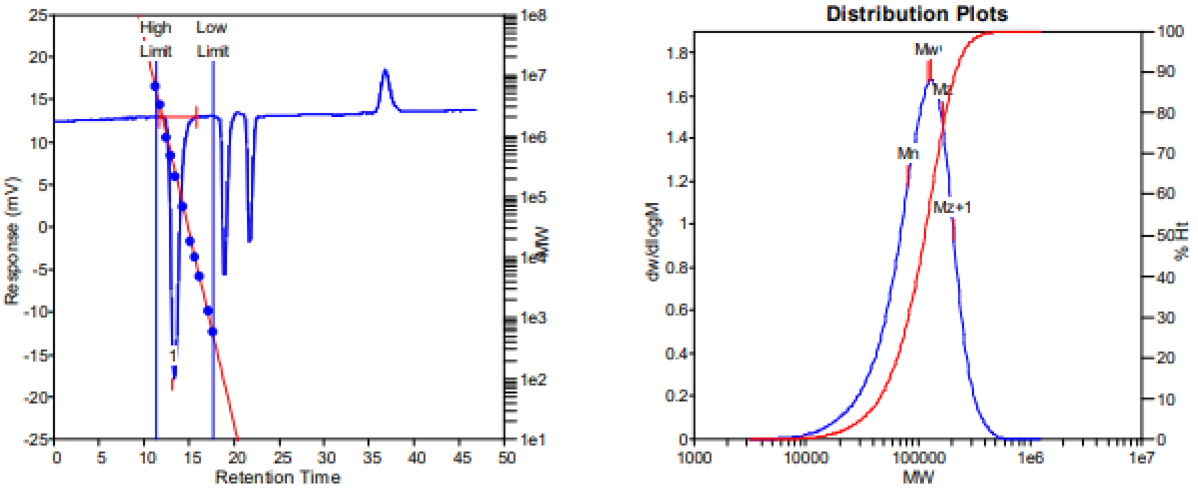

MW Averages

Peak No	Mp	Mn	Mw	Mz	Mz+1	Mv	PD
1	130948	81252	124145	164993	209930	118806	1.5279

Processed Peaks

Peak No	Name	Start RT (mins)	Max RT (mins)	End RT (mins)	Pk Height (mV)	% Height	Area (mV.secs)	% Area
1		11.70	13.28	15.92	-30.8364	100	1792.76	100

Figure S37. GPC trace of the polymer from table 1, entry 4.

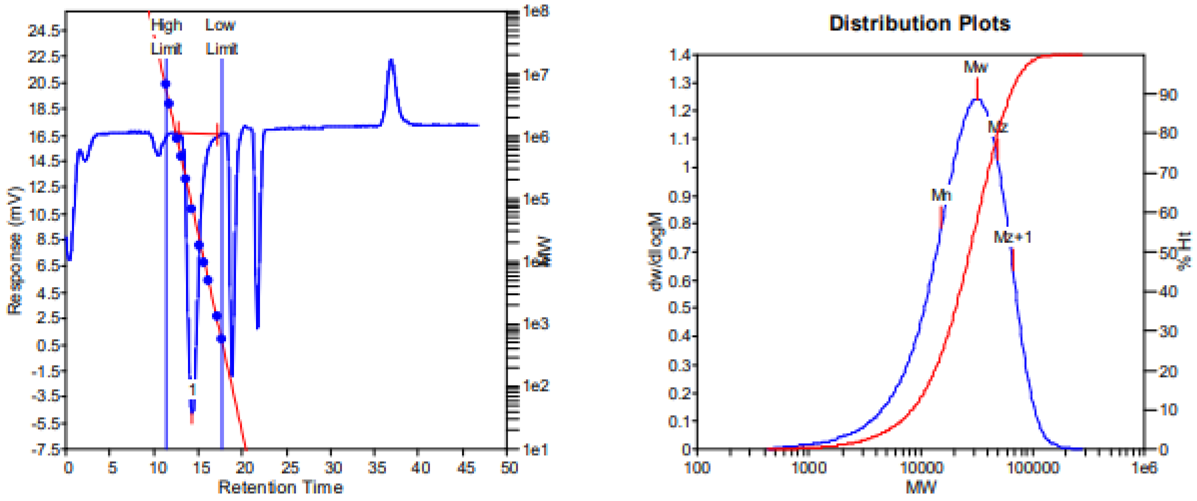

MW Averages

Peak No	Mp	Mn	Mw	Mz	Mz+1	Mv	PD
1	31673	15281	32381	49166	66955	30255	2.11904

Processed Peaks

Peak No	Name	Start RT (mins)	Max RT (mins)	End RT (mins)	Pk Height (mV)	% Height	Area (mV.secs)	% Area
1		12.75	14.28	17.32	-21.4062	100	1677.56	100

Figure S38. GPC trace of the polymer from table 1, entry 5.

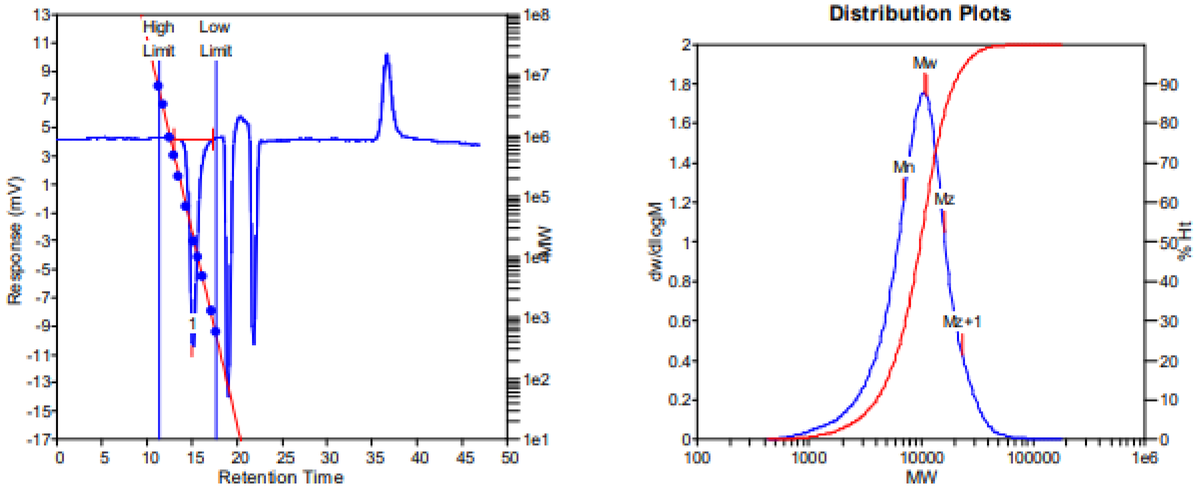

MW Averages

Peak No	Mp	Mn	Mw	Mz	Mz+1	Mv	PD
1	10668	7072	11165	16006	23419	10610	1.57876

Processed Peaks

Peak No	Name	Start RT (mins)	Max RT (mins)	End RT (mins)	Pk Height (mV)	% Height	Area (mV.secs)	% Area
1		13.05	15.05	17.32	-14.6353	100	812.022	100

Figure S39. GPC trace of the polymer from table 1, entry 6.

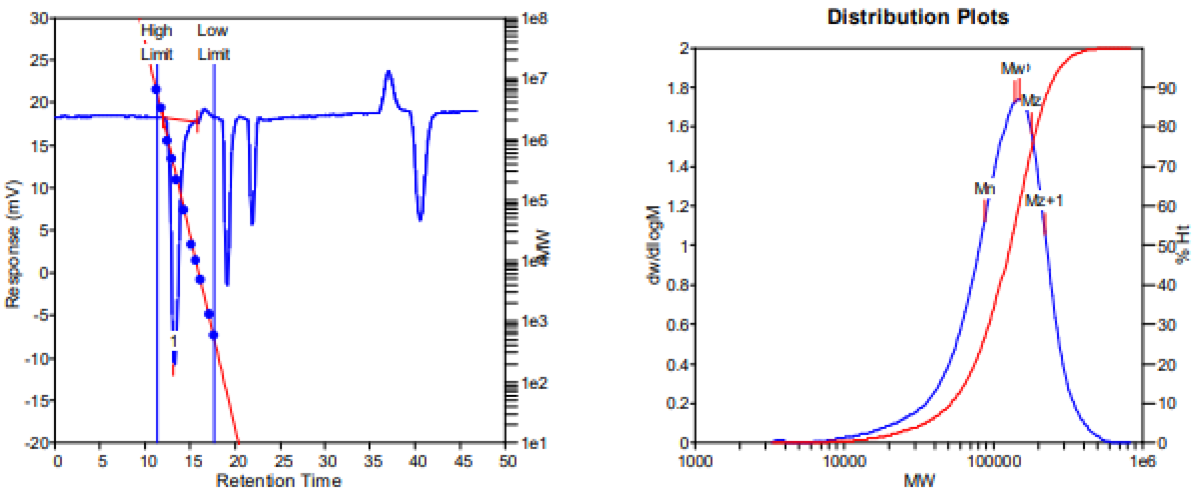

MW Averages

Peak No	Mp	Mn	Mw	Mz	Mz+1	Mv	PD
1	147390	87555	137526	179650	221659	131827	1.57074

Processed Peaks

Peak No	Name	Start RT (mins)	Max RT (mins)	End RT (mins)	Pk Height (mV)	% Height	Area (mV.secs)	% Area
1		11.98	13.20	15.88	-28.9014	100	1614.49	100

Figure S40. GPC trace of the polymer from table 1, entry 7.

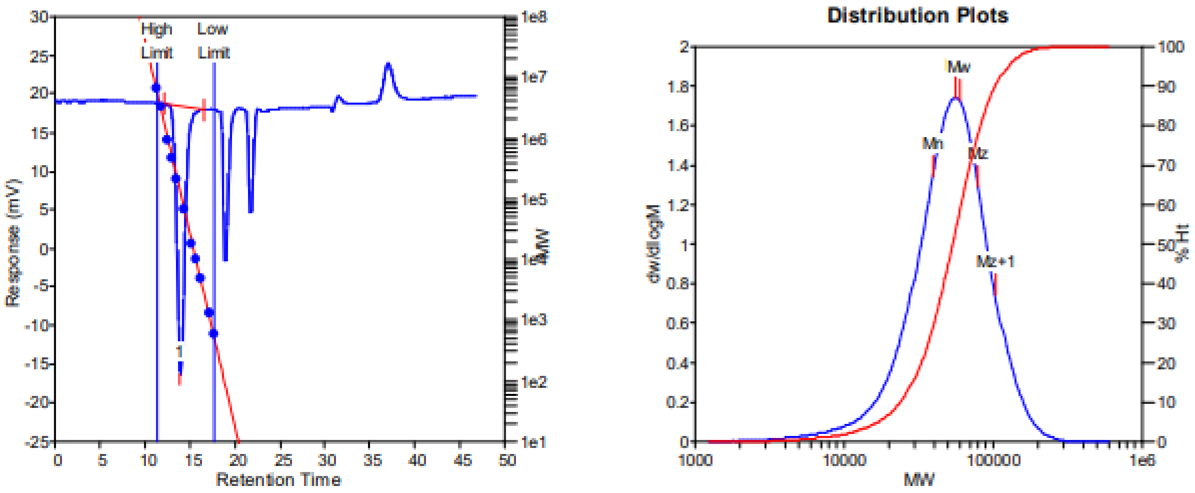

MW Averages

Peak No	Mp	Mn	Mw	Mz	Mz+1	Mv	PD
1	55880	39034	59444	79678	103859	56914	1.52288

Processed Peaks

Peak No	Name	Start RT (mins)	Max RT (mins)	End RT (mins)	Pk Height (mV)	% Height	Area (mV.secs)	% Area
1		12.22	13.88	16.57	-34.6129	100	1933.44	100

Figure S41. GPC trace of the polymer from table 1, entry 8.

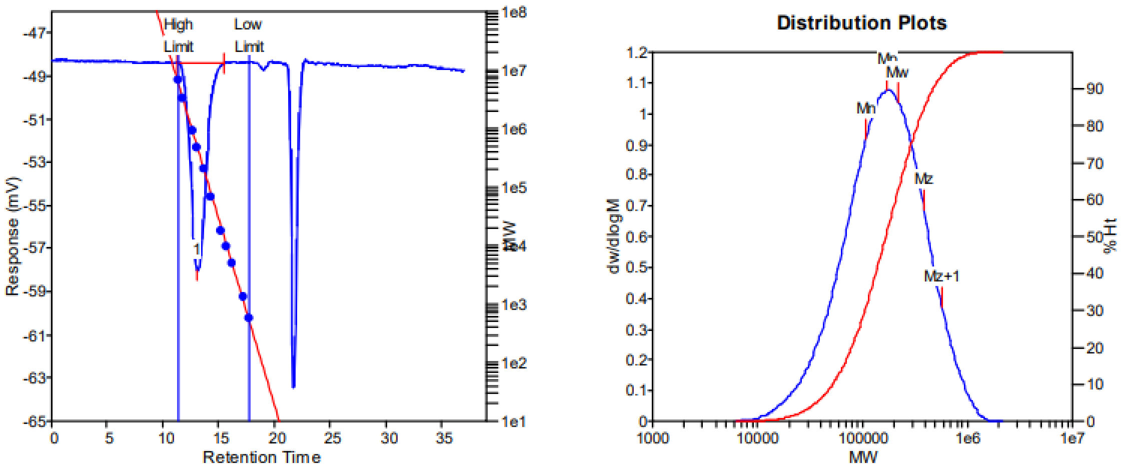

MW Averages

Peak No	Mp	Mn	Mw	Mz	Mz+1	Mv	PD
1	173933	107378	221824	387157	575050	203421	2.06582

Processed Peaks

Peak No	Name	Start RT (mins)	Max RT (mins)	End RT (mins)	Pk Height (mV)	% Height	Area (mV.secs)	% Area
1		11.30	13.08	15.43	-9.59515	100	868.189	100

Figure S42. GPC trace of the polymer from table 1, entry 9.

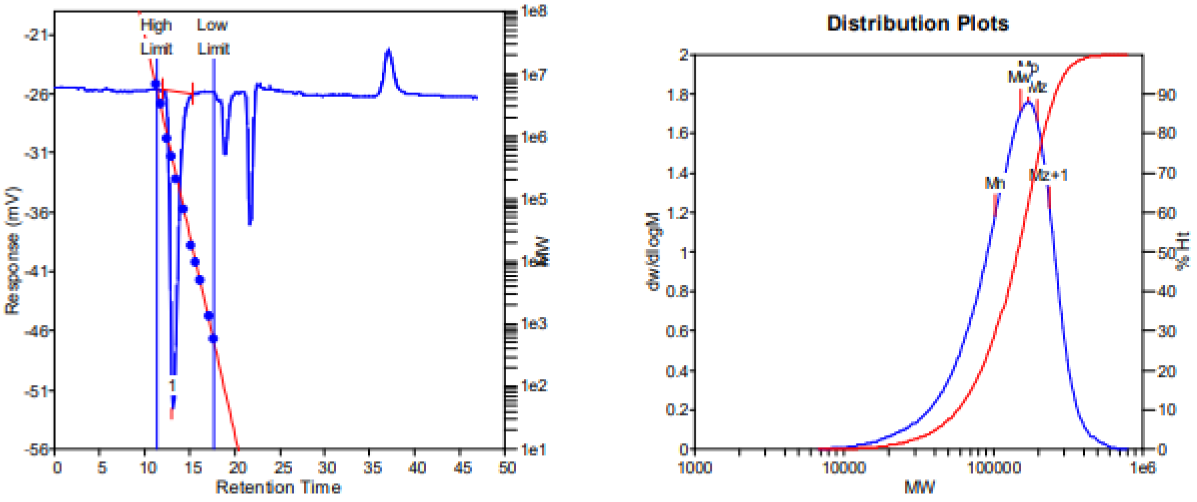

MW Averages

Peak No	Mp	Mn	Mw	Mz	Mz+1	Mv	PD
1	169867	103074	152286	195870	238018	146327	1.47744

Processed Peaks

Peak No	Name	Start RT (mins)	Max RT (mins)	End RT (mins)	Pk Height (mV)	% Height	Area (mV.secs)	% Area
1		12.02	13.10	15.37	-26.8437	0	1484.4	100

Figure S43. GPC trace of the polymer from table 2, entry 1.

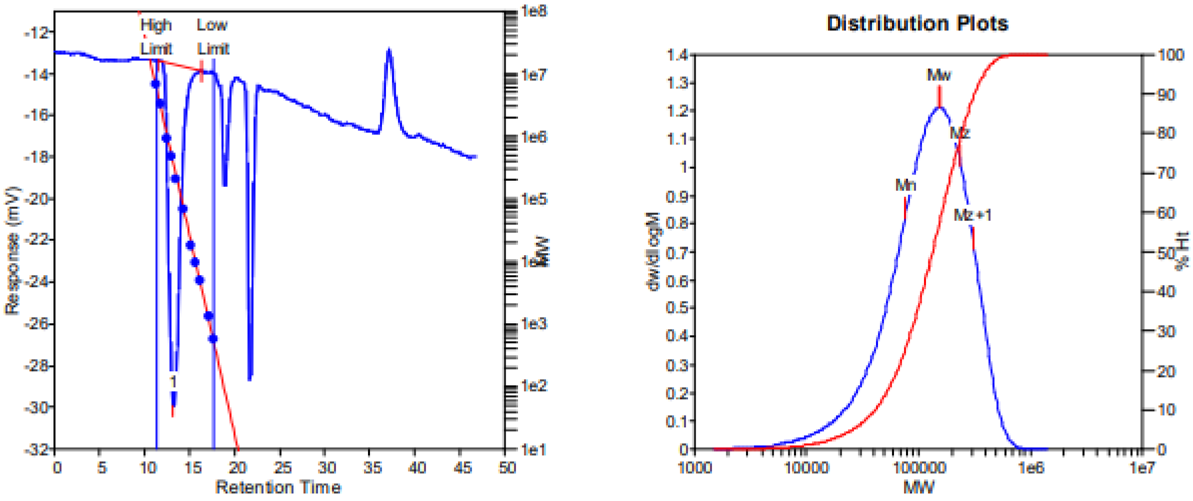

MW Averages

Peak No	Mp	Mn	Mw	Mz	Mz+1	Mv	PD
1	150918	76254	158325	238232	314904	147988	2.07628

Processed Peaks

Peak No	Name	Start RT (mins)	Max RT (mins)	End RT (mins)	Pk Height (mV)	% Height	Area (mV.secs)	% Area
1		11.60	13.18	16.43	-16.3726	0	1314.31	100

Figure S44. GPC trace of the polymer from table 2, entry 2.

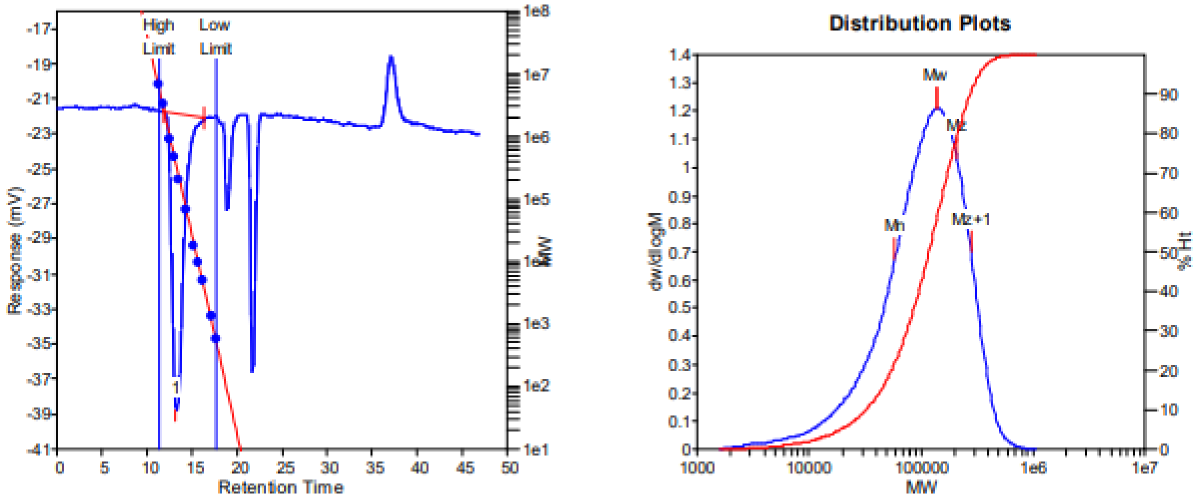

MW Averages

Peak No	Mp	Mn	Mw	Mz	Mz+1	Mv	PD
1	140579	59024	137930	210017	279574	128507	2.33685

Processed Peaks

Peak No	Name	Start RT (mins)	Max RT (mins)	End RT (mins)	Pk Height (mV)	% Height	Area (mV.secs)	% Area
1		11.80	13.23	16.38	-16.8482	0	1355.3	100

Figure S45. GPC trace of the polymer from table 2, entry 3.

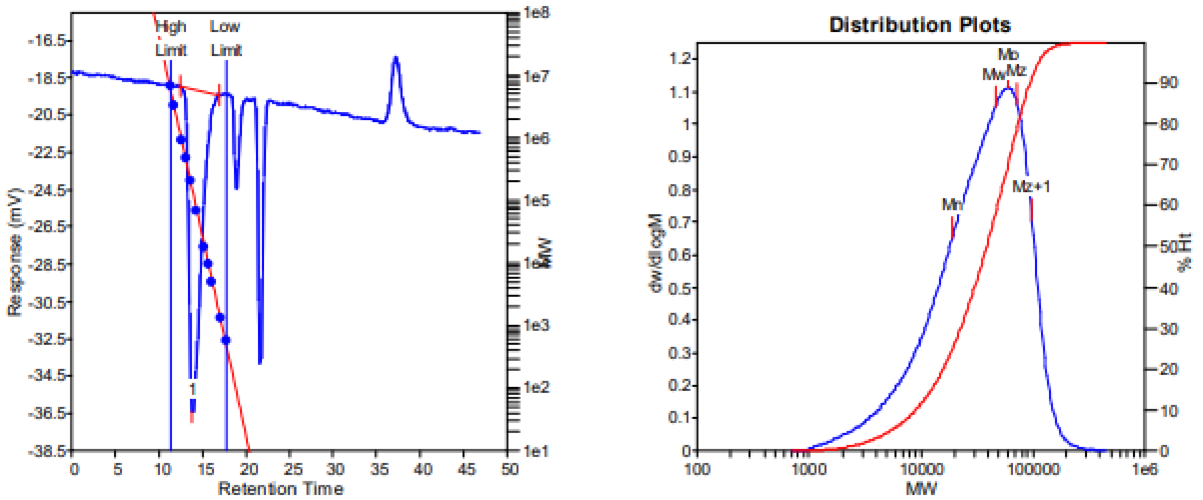

MW Averages

Peak No	Mp	Mn	Mw	Mz	Mz+1	Mv	PD
1	59989	19027	45928	72666	98274	42419	2.41383

Processed Peaks

Peak No	Name	Start RT (mins)	Max RT (mins)	End RT (mins)	Pk Height (mV)	% Height	Area (mV.secs)	% Area
1		12.42	13.83	16.97	-17.3429	0	1517.01	100

Figure S46. GPC trace of the polymer from table 2, entry 4.

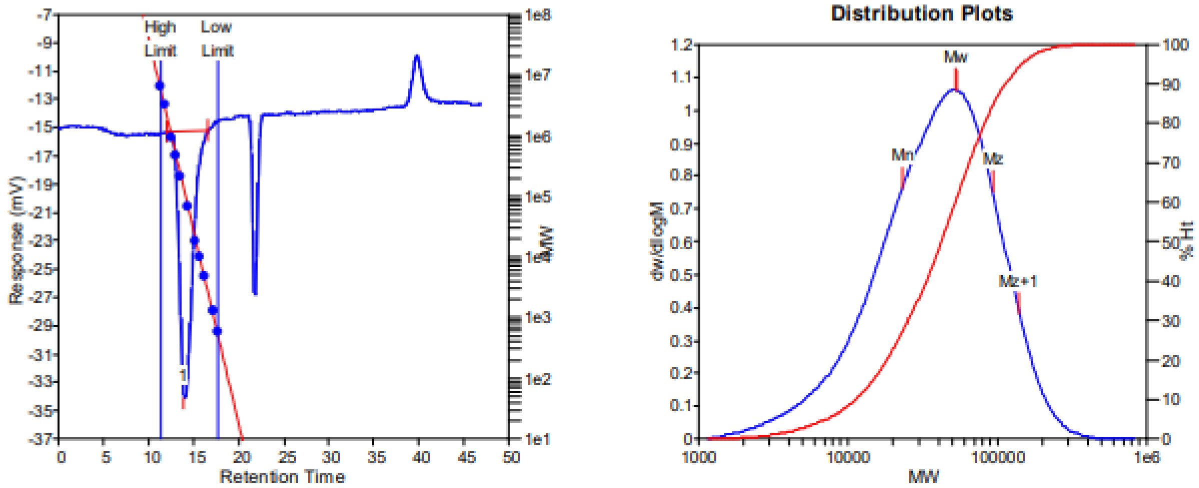

MW Averages

Peak No	Mp	Mn	Mw	Mz	Mz+1	Mv	PD
1	52051	23178	53784	93417	139778	49235	2.32048

Processed Peaks

Peak No	Name	Start RT (mins)	Max RT (mins)	End RT (mins)	Pk Height (mV)	% Height	Area (mV.secs)	% Area
1		11.98	13.93	16.62	-18.9129	0	1735.25	100

Figure S47. GPC trace of the polymer from table 2, entry 5.

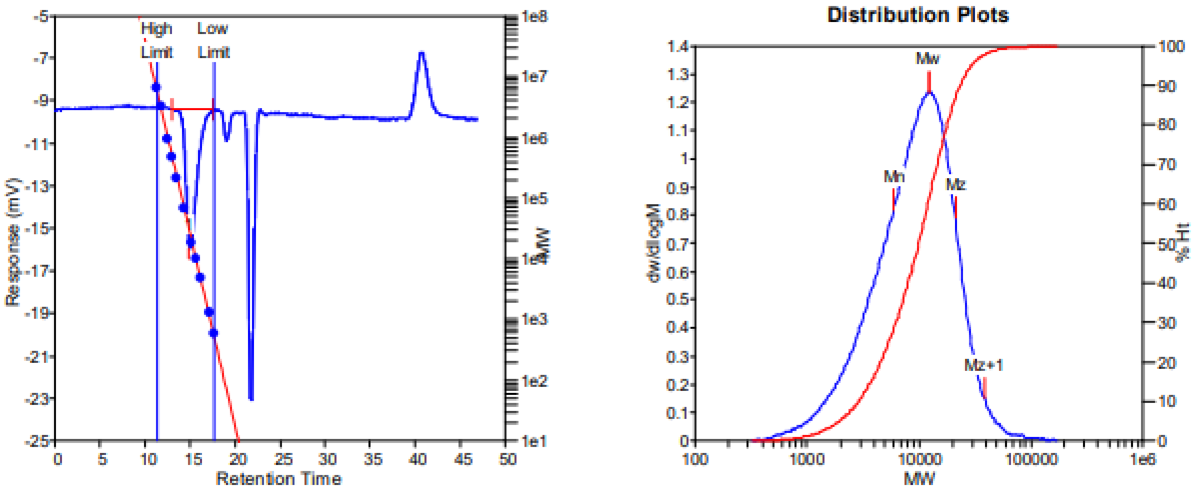

MW Averages

Peak No	Mp	Mn	Mw	Mz	Mz+1	Mv	PD
1	12295	5976	12072	21054	38271	11153	2.02008

Processed Peaks

Peak No	Name	Start RT (mins)	Max RT (mins)	End RT (mins)	Pk Height (mV)	% Height	Area (mV.secs)	% Area
1		13.10	14.95	17.50	-6.52758	100	513.823	100

Figure S48. GPC trace of the polymer from table 2, entry 7.

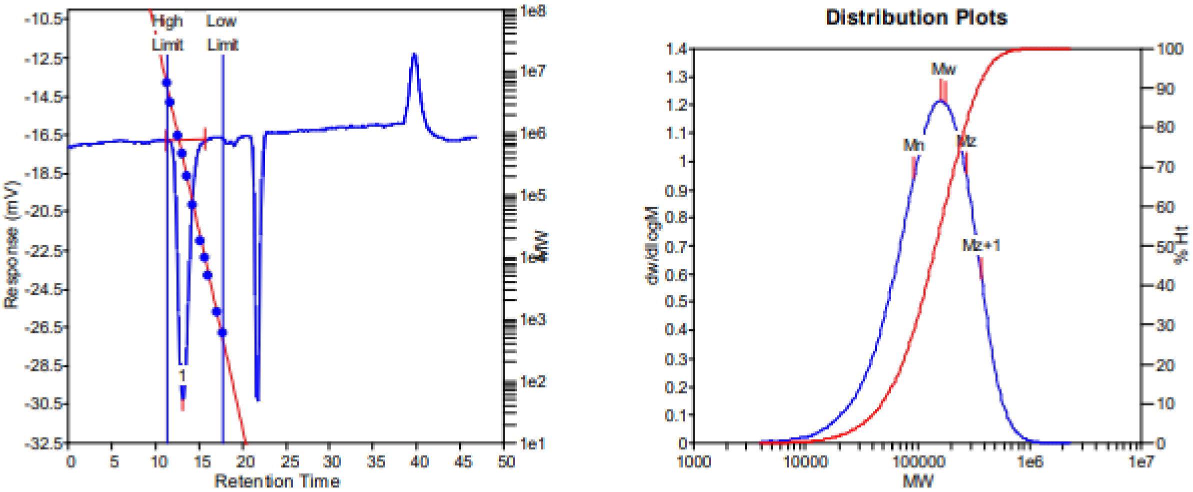

MW Averages

Peak No	Mp	Mn	Mw	Mz	Mz+1	Mv	PD
1	162017	92458	175511	270085	374297	164003	1.89828

Processed Peaks

Peak No	Name	Start RT (mins)	Max RT (mins)	End RT (mins)	Pk Height (mV)	% Height	Area (mV.secs)	% Area
1		11.25	13.13	15.75	-13.4546	100	1077.22	100

Figure S49. GPC trace of the polymer from table 2, entry 9.

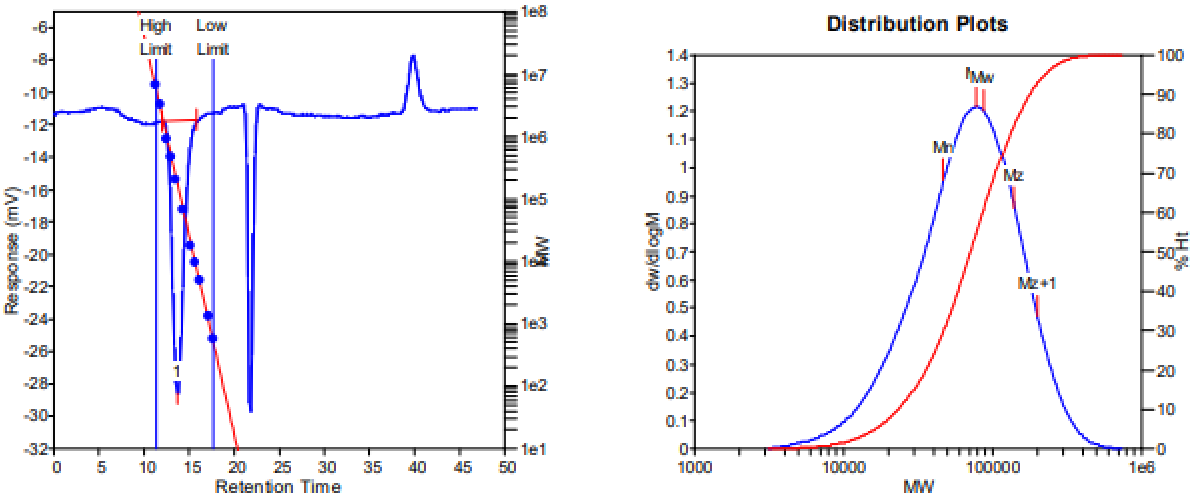

MW Averages

Peak No	Mp	Mn	Mw	Mz	Mz+1	Mv	PD
1	77818	46886	87927	140044	200152	81831	1.87534

Processed Peaks

Peak No	Name	Start RT (mins)	Max RT (mins)	End RT (mins)	Pk Height (mV)	% Height	Area (mV.secs)	% Area
1		12.07	13.63	15.92	-16.8506	0	1349.09	100

Figure S50. GPC trace of the polymer from table 2, entry 10.

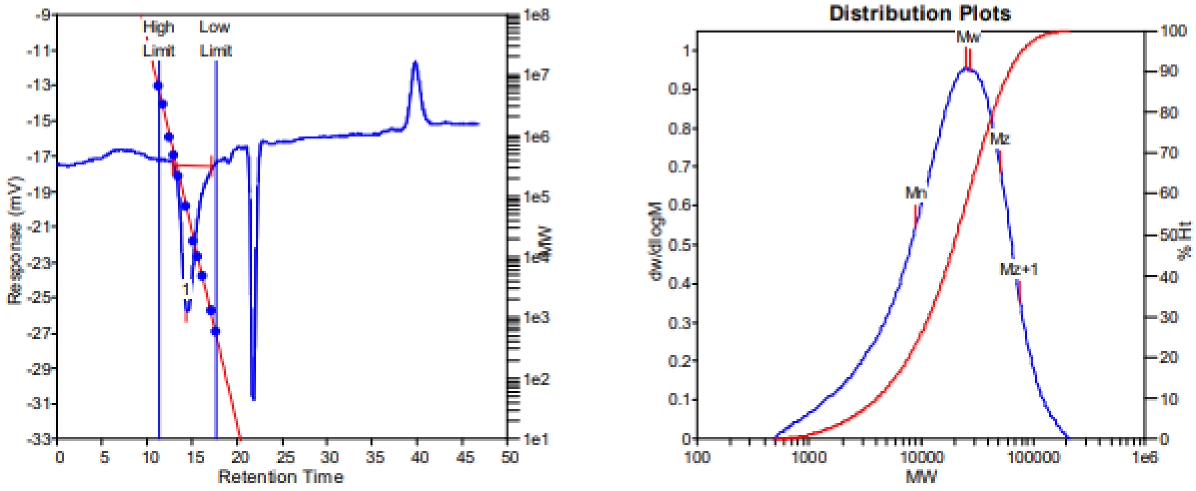

MW Averages

Peak No	Mp	Mn	Mw	Mz	Mz+1	Mv	PD
1	25001	9081	27814	51694	76651	25029	3.06288

Processed Peaks

Peak No	Name	Start RT (mins)	Max RT (mins)	End RT (mins)	Pk Height (mV)	% Height	Area (mV.secs)	% Area
1		12.95	14.45	17.22	-8.21542	100	838.847	100

Figure S51. GPC trace of the polymer from table 2, entry 11.

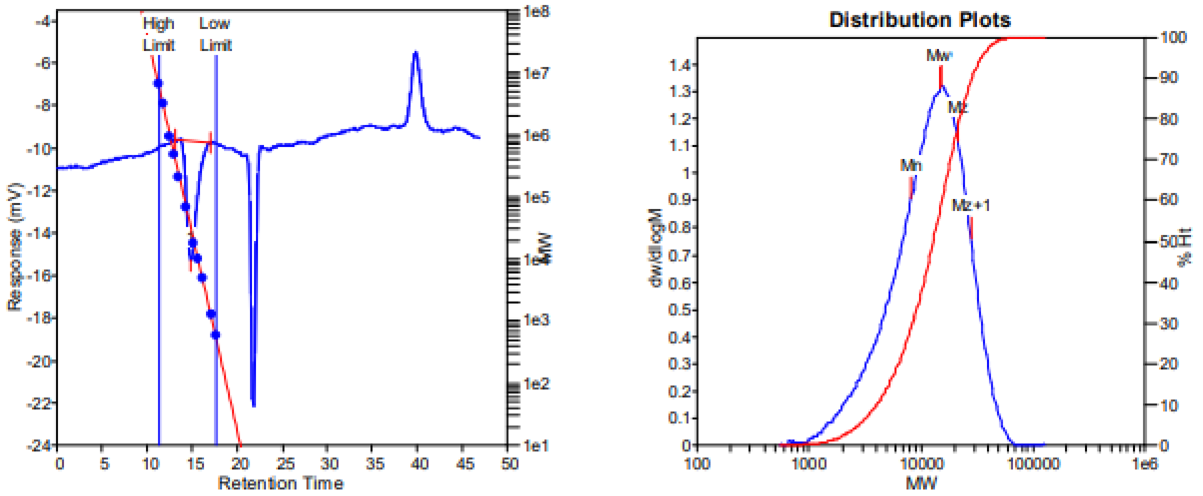

MW Averages

Peak No	Mp	Mn	Mw	Mz	Mz+1	Mv	PD
1	15213	8264	14760	21475	27938	13887	1.78606

Processed Peaks

Peak No	Name	Start RT (mins)	Max RT (mins)	End RT (mins)	Pk Height (mV)	% Height	Area (mV.secs)	% Area
1		13.28	14.80	17.13	-5.63518	100	414.718	100

Figure S52. GPC trace of the polymer from table 2, entry 12.

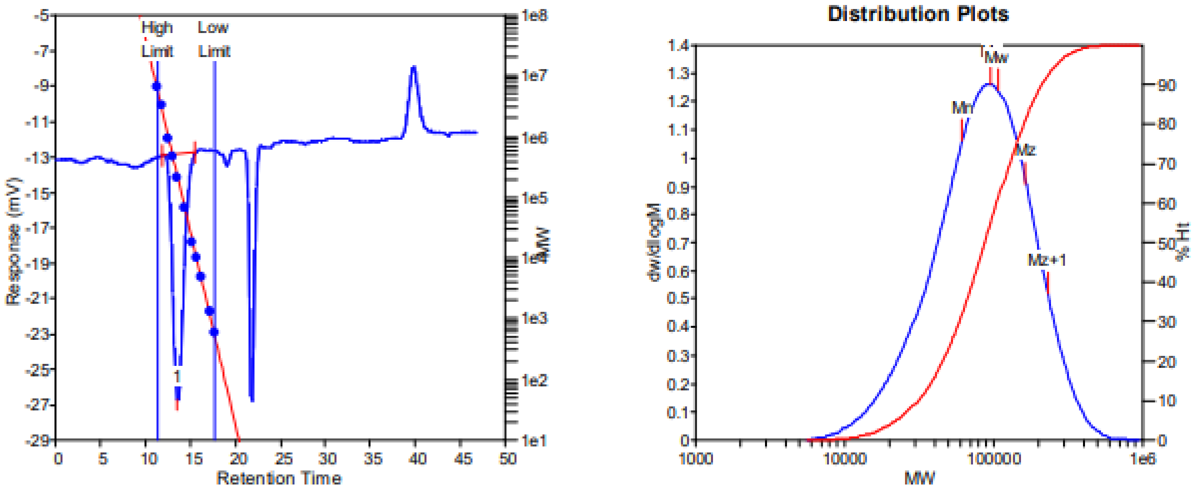

MW Averages

Peak No	Mp	Mn	Mw	Mz	Mz+1	Mv	PD
1	96282	61545	106798	164687	231791	99986	1.73528

Processed Peaks

Peak No	Name	Start RT (mins)	Max RT (mins)	End RT (mins)	Pk Height (mV)	% Height	Area (mV.secs)	% Area
1		11.88	13.50	15.50	-13.8669	100	1068.98	100

Figure S53. GPC trace of the polymer from table 2, entry 13.

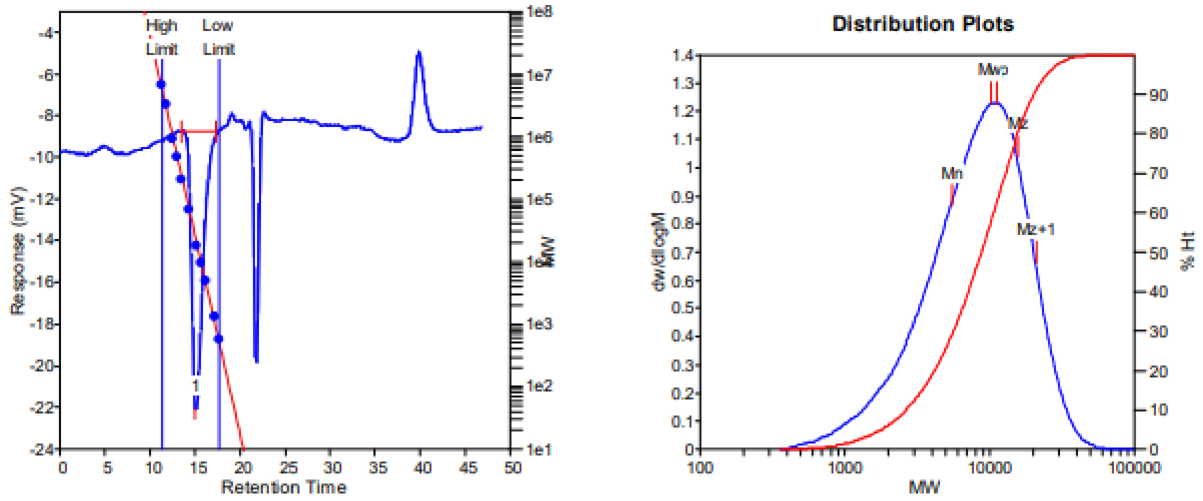

MW Averages

Peak No	Mp	Mn	Mw	Mz	Mz+1	Mv	PD
1	11185	5426	10324	15607	20851	9653	1.90269

Processed Peaks

Peak No	Name	Start RT (mins)	Max RT (mins)	End RT (mins)	Pk Height (mV)	% Height	Area (mV.secs)	% Area
1		13.47	15.02	17.45	-13.2931	0	1050.72	100

Figure S54. GPC trace of the polymer from table 2, entry 14.

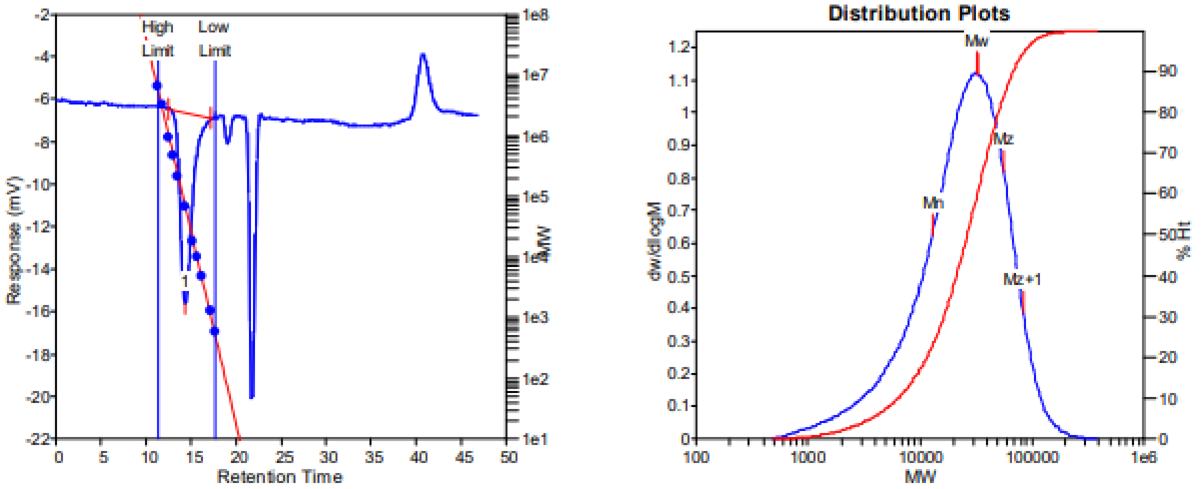

MW Averages

Peak No	Mp	Mn	Mw	Mz	Mz+1	Mv	PD
1	31673	12829	32894	55851	83290	30179	2.56403

Processed Peaks

Peak No	Name	Start RT (mins)	Max RT (mins)	End RT (mins)	Pk Height (mV)	% Height	Area (mV.secs)	% Area
1		12.53	14.30	17.22	-8.99194	0	781.864	100

Figure S55. GPC trace of the polymer from table 2, entry 15.

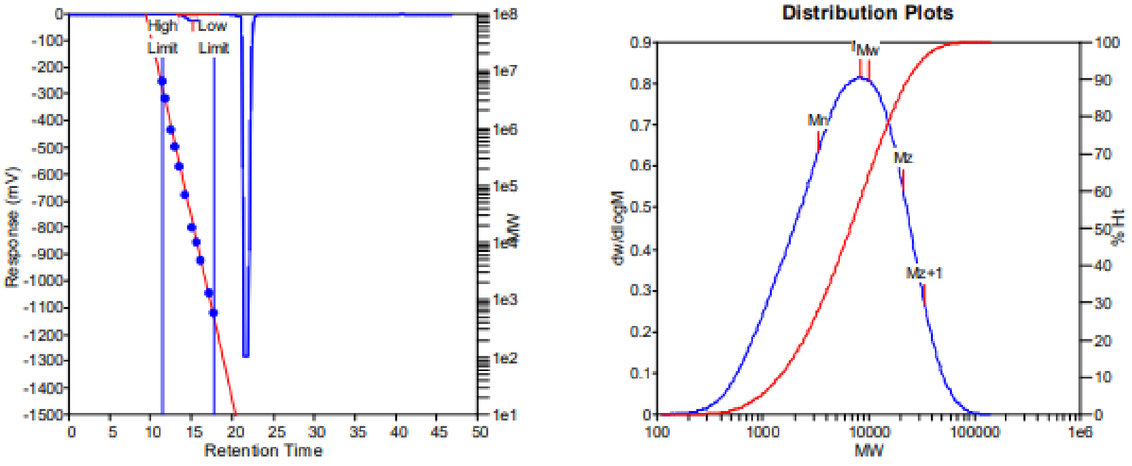

MW Averages

Peak No	Mp	Mn	Mw	Mz	Mz+1	Mv	PD
1	8421	3351	10138	21304	34015	8956	3.02537

Processed Peaks

Peak No	Name	Start RT (mins)	Max RT (mins)	End RT (mins)	Pk Height (mV)	% Height	Area (mV.secs)	% Area
1		13.23	15.22	18.27	-21.3237	100	2551.34	100

Figure S56. GPC trace of the polymer from table 2, entry 16.

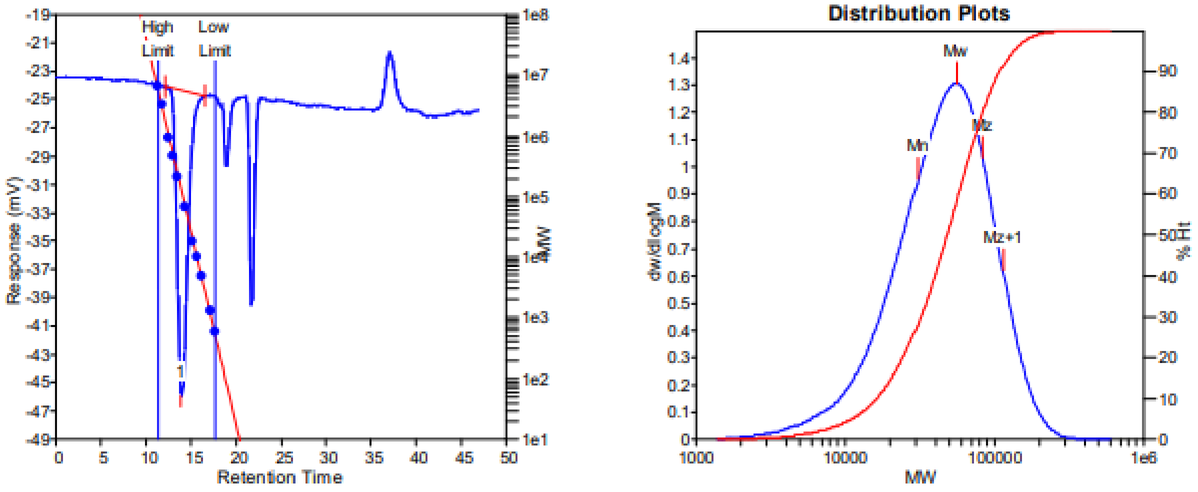

MW Averages

Peak No	Mp	Mn	Mw	Mz	Mz+1	Mv	PD
1	55880	30712	56205	83733	113705	52787	1.83007

Processed Peaks

Peak No	Name	Start RT (mins)	Max RT (mins)	End RT (mins)	Pk Height (mV)	% Height	Area (mV.secs)	% Area
1		12.22	13.88	16.48	-21.527	0	1603.89	100

Figure S57. GPC trace of the polymer from table 2, entry 17.

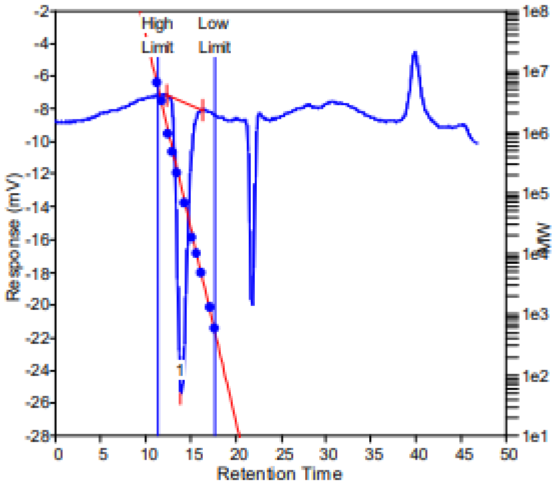

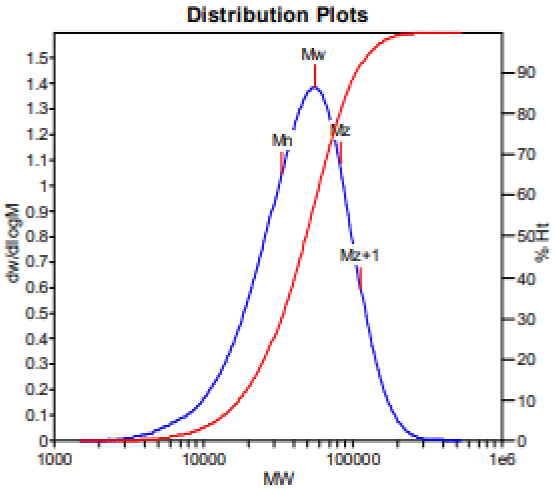

MW Averages

Peak No	Mp	Mn	Mw	Mz	Mz+1	Mv	PD
1	55880	33189	56113	82224	113522	52935	1.69071

Processed Peaks

Peak No	Name	Start RT (mins)	Max RT (mins)	End RT (mins)	Pk Height (mV)	% Height	Area (mV.secs)	% Area
1		12.30	13.88	16.43	-17.9249	100	1259.7	100

Figure S58. GPC trace of the polymer from table 2, entry 18.

5. DSC Data of Polymers

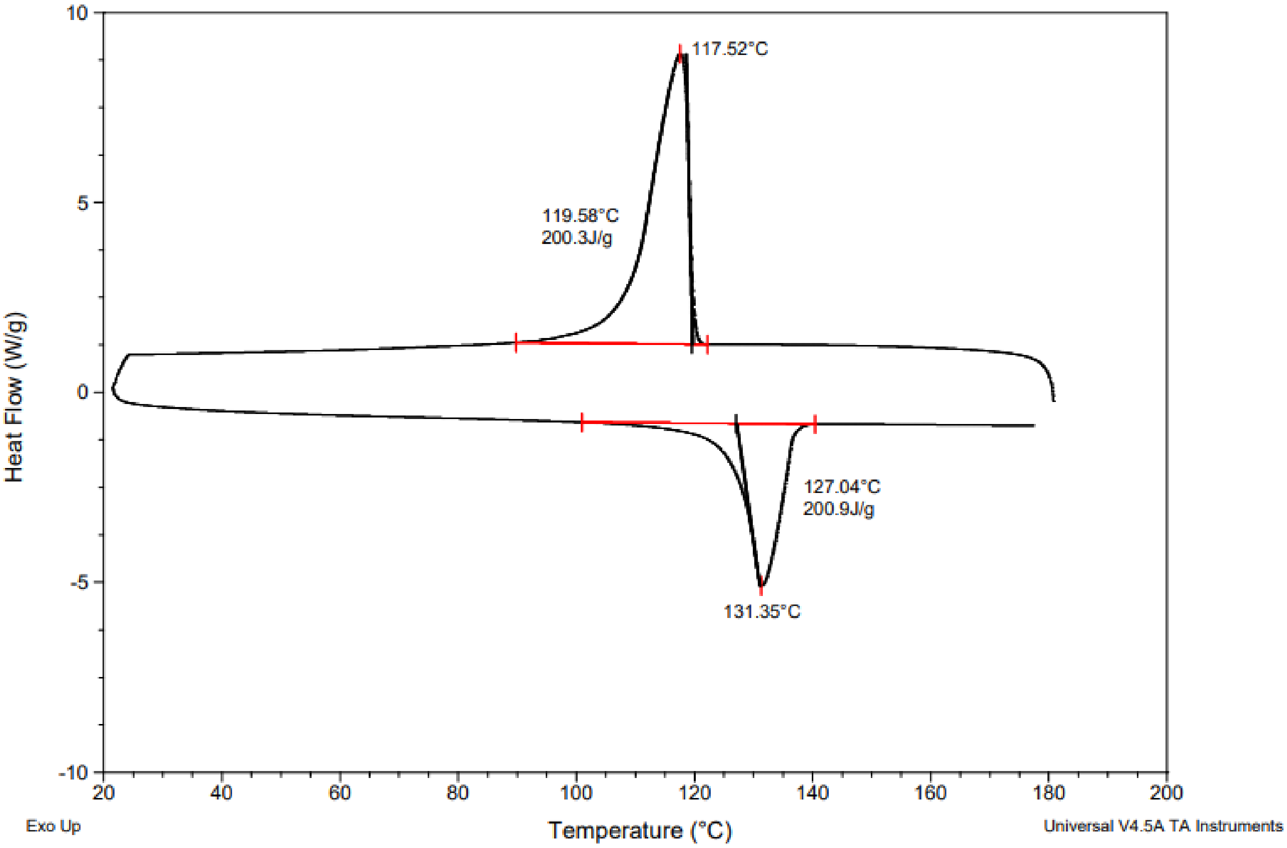

Figure S59. DSC data of the polymer from table 1, entry 1.

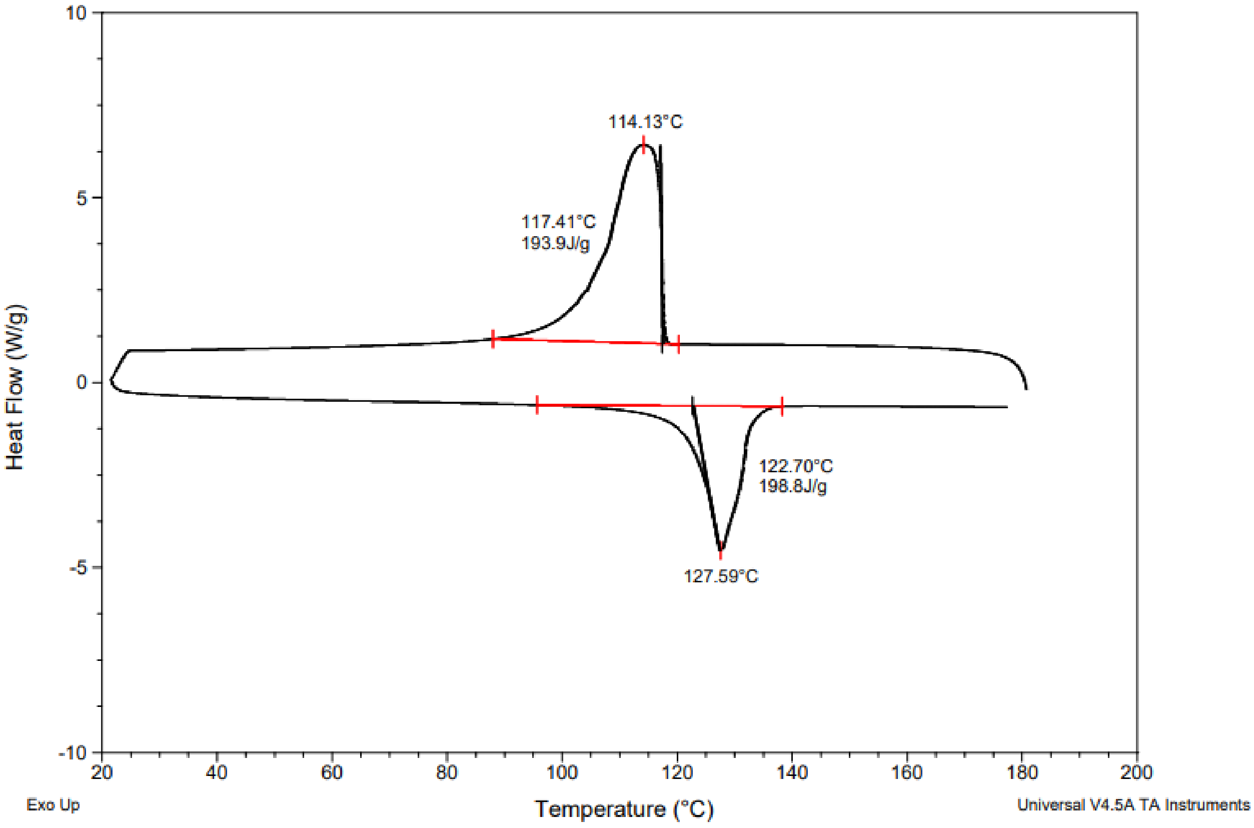

Figure S60. DSC data of the polymer from table 1, entry 2.

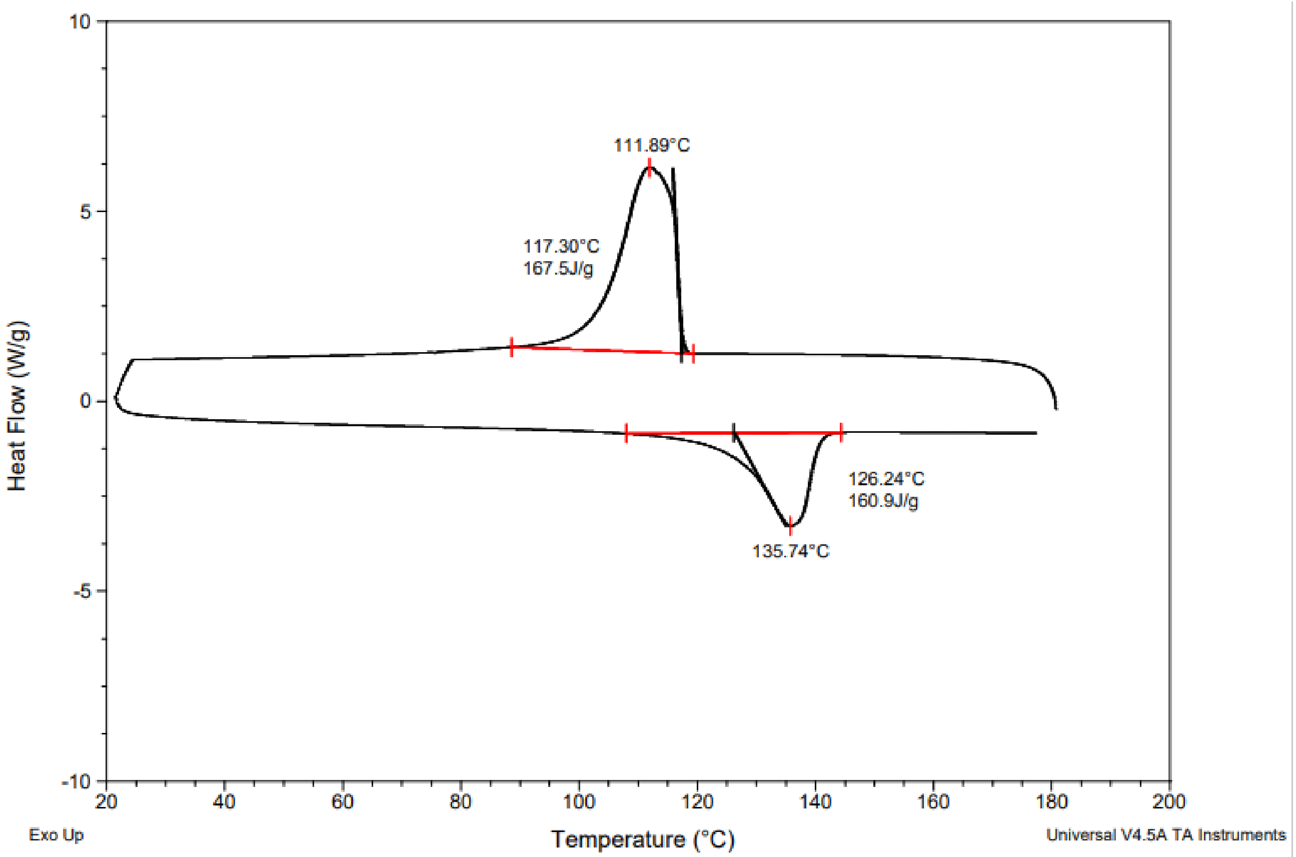

Figure S61. DSC data of the polymer from table 1, entry 3.

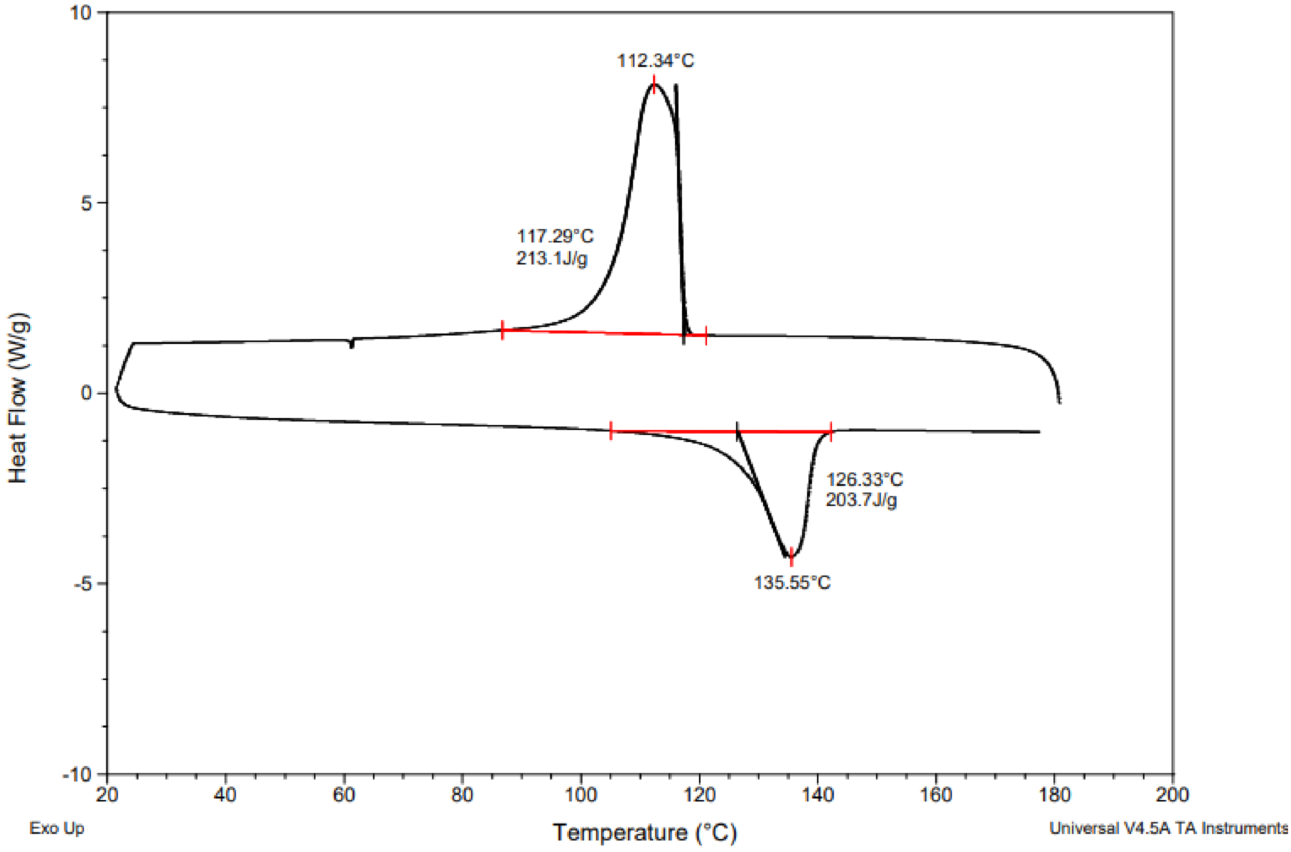

Figure S62. DSC data of the polymer from table 1, entry 4.

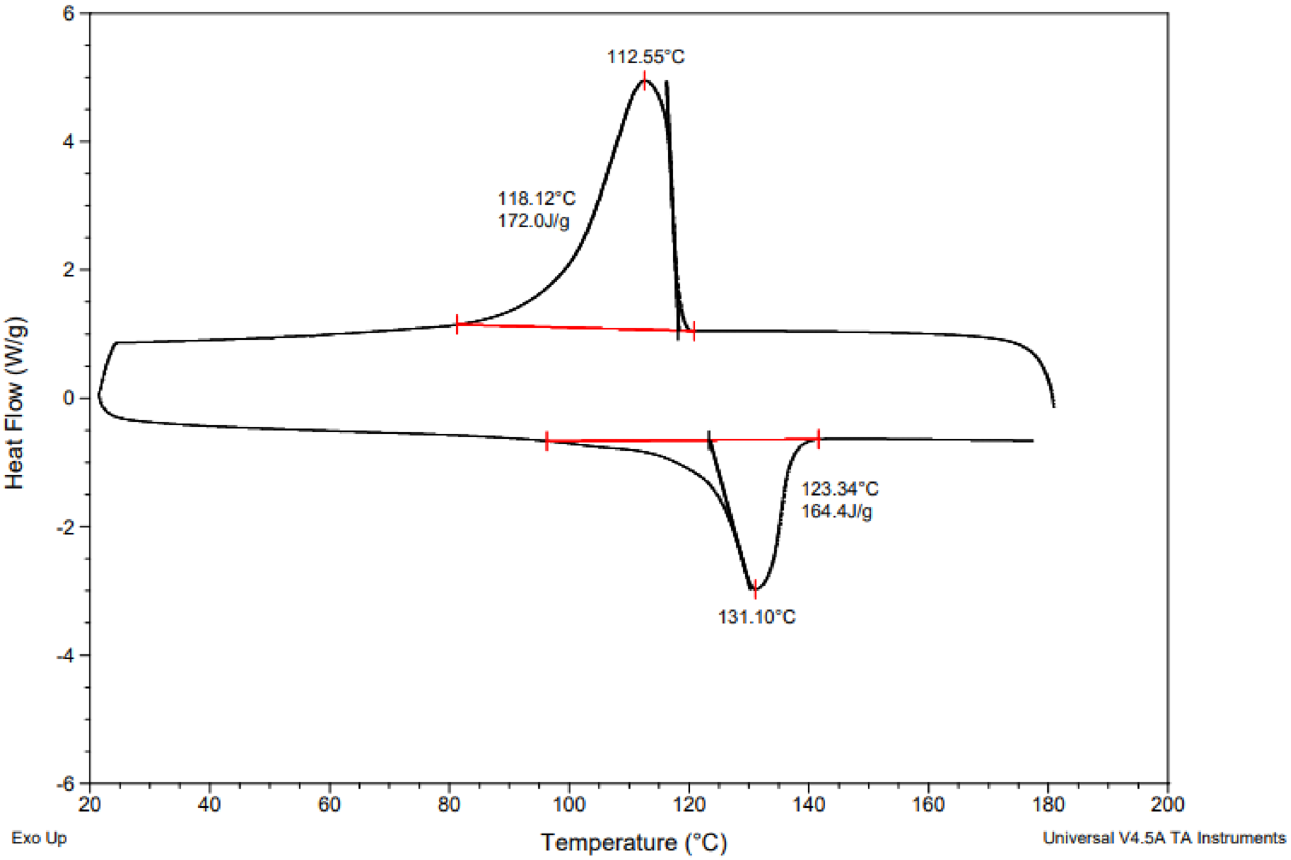

Figure S63. DSC data of the polymer from table 1, entry 5

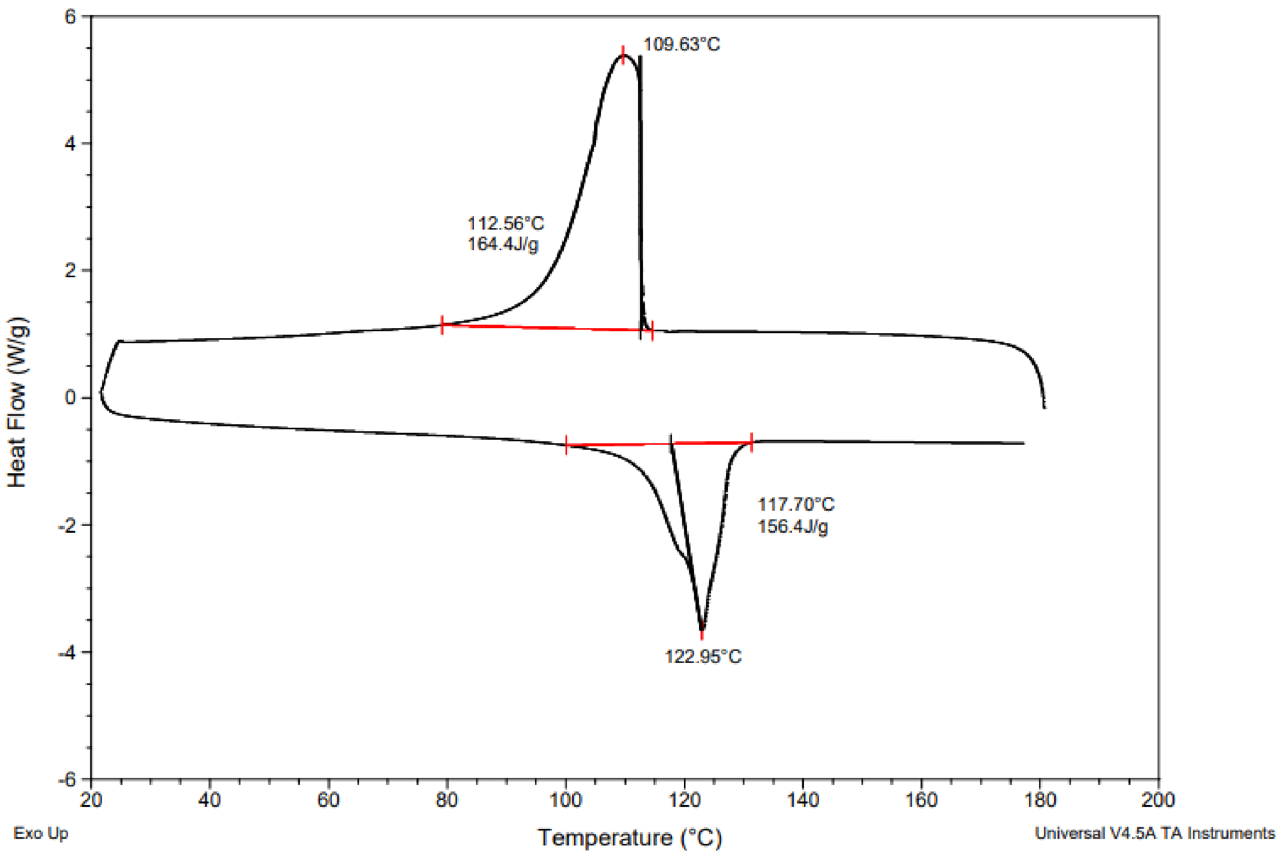

Figure S64. DSC data of the polymer from table 1, entry 6

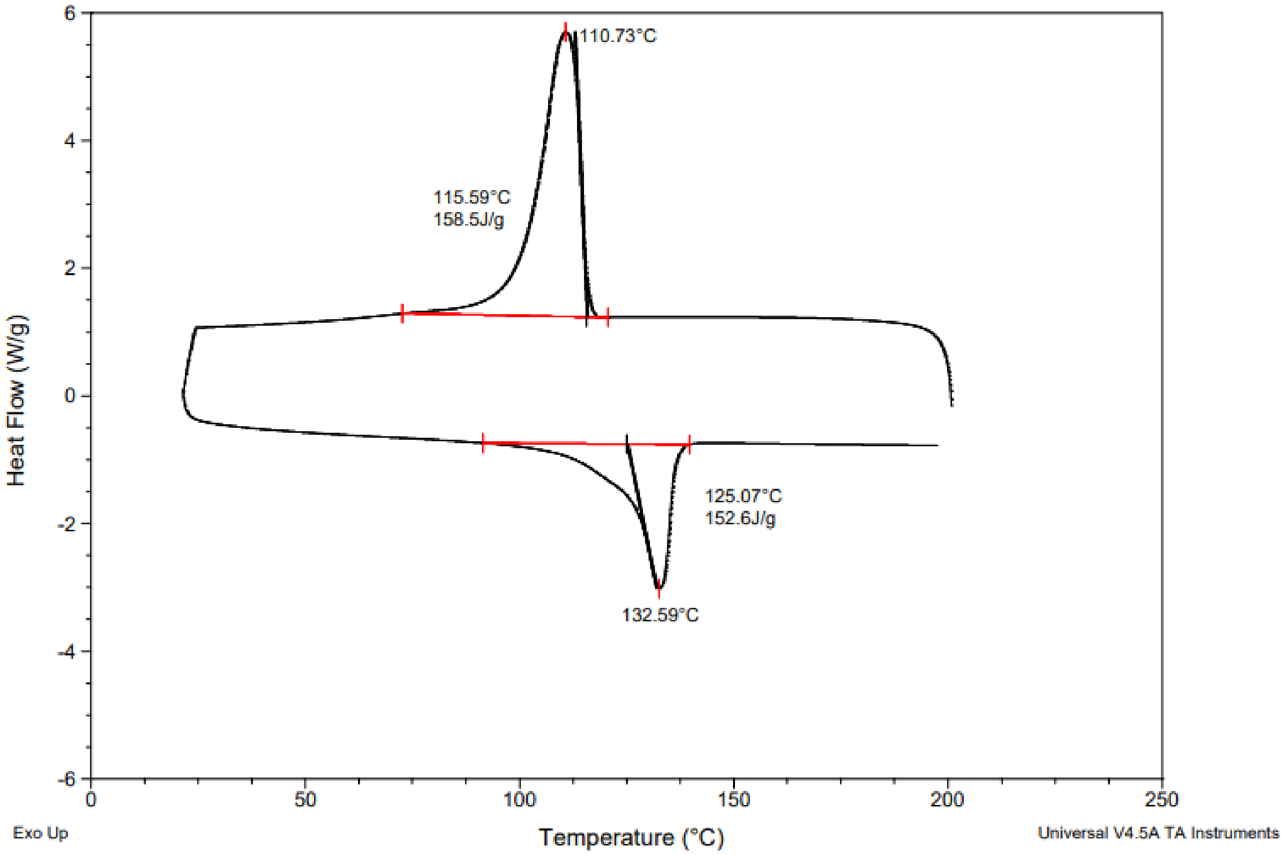

Figure S65. DSC data of the polymer from table 1, entry 7

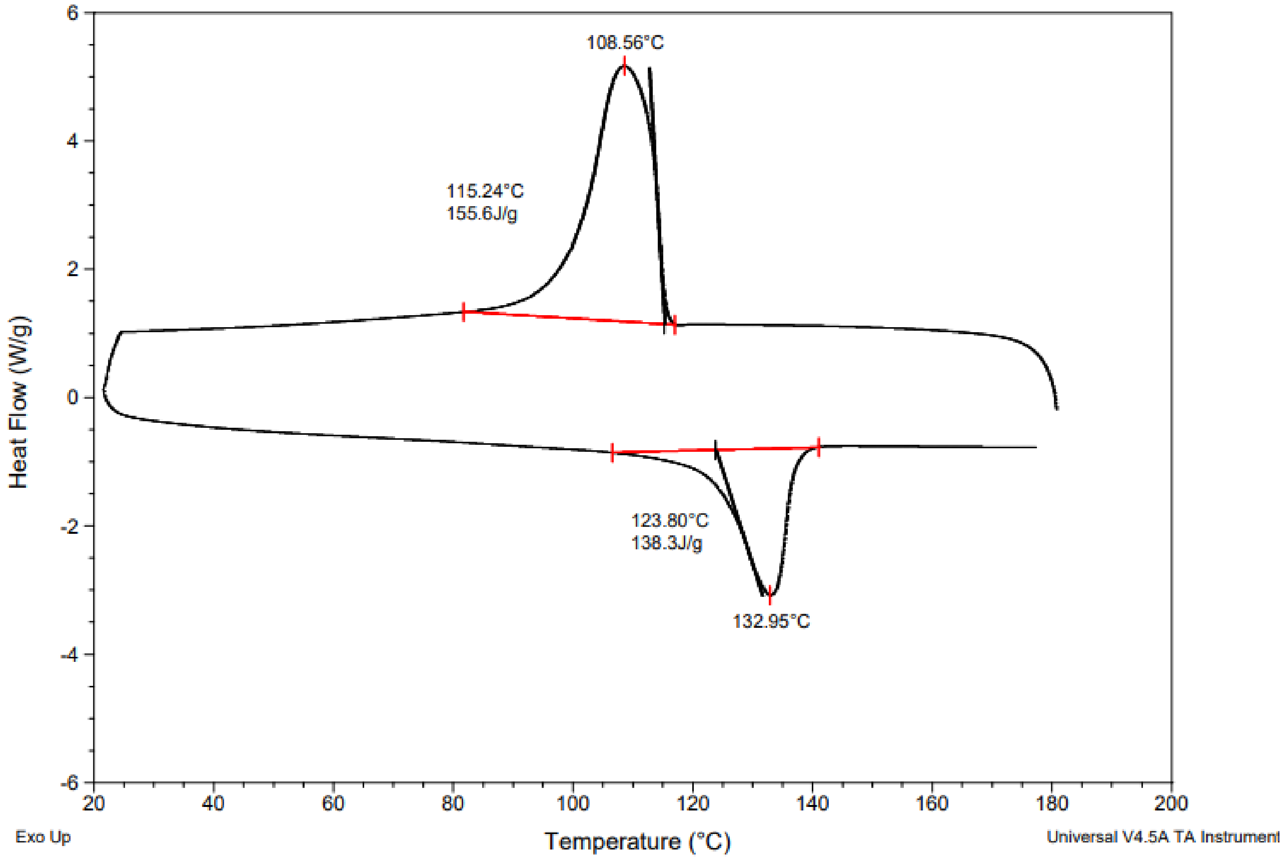

Figure S66. DSC data of the polymer from table 1, entry 8

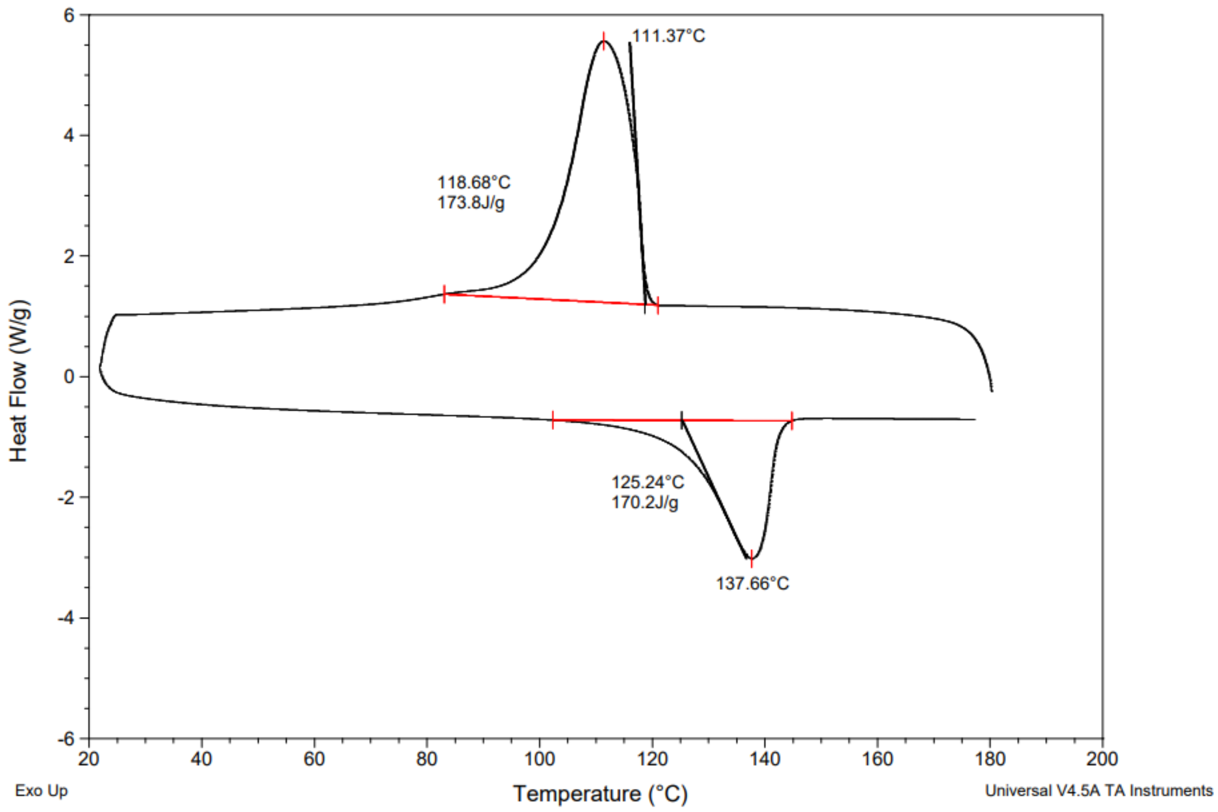

Figure S67. DSC data of the polymer from table 1, entry 9.

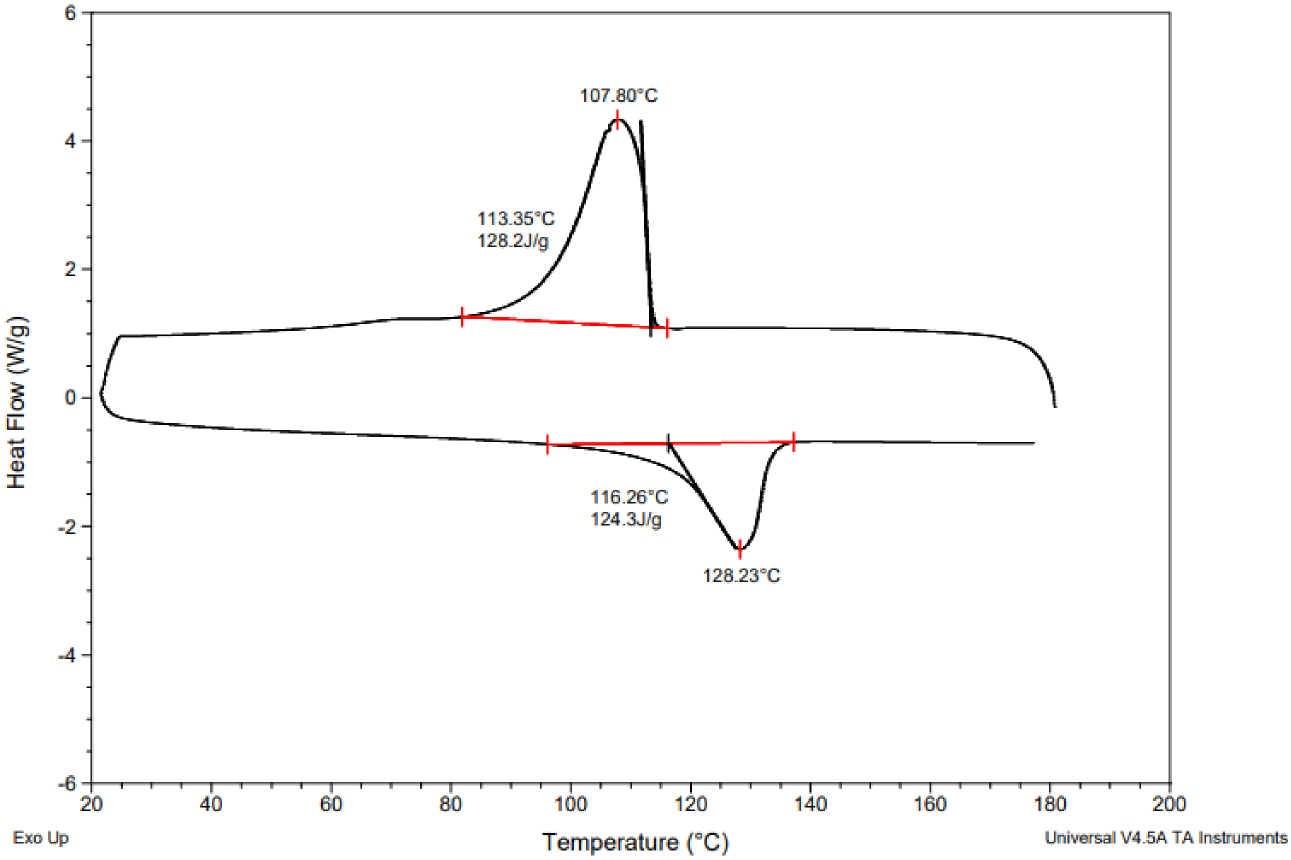

Figure S68. DSC data of the polymer from table 2, entry 1

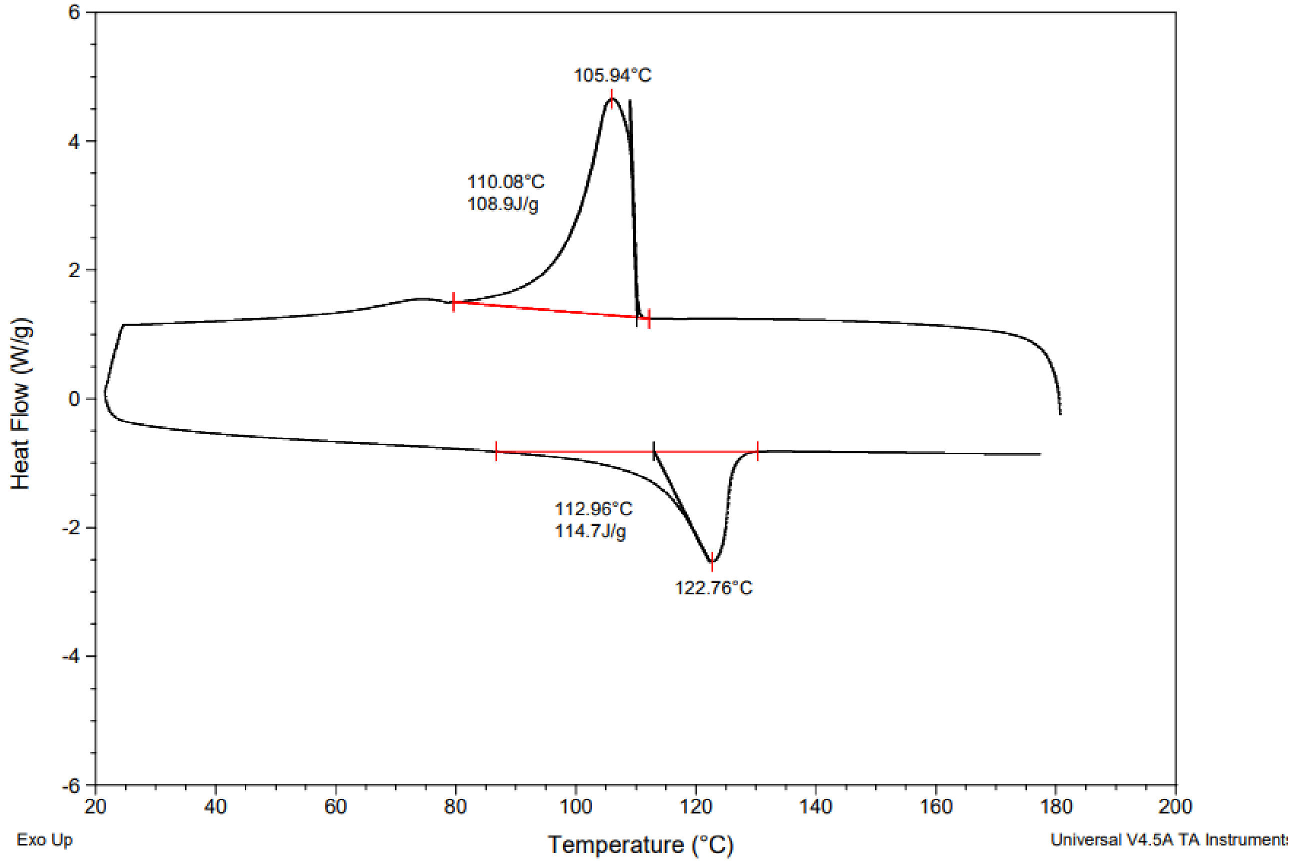

Figure S69. DSC data of the polymer from table 2, entry 2

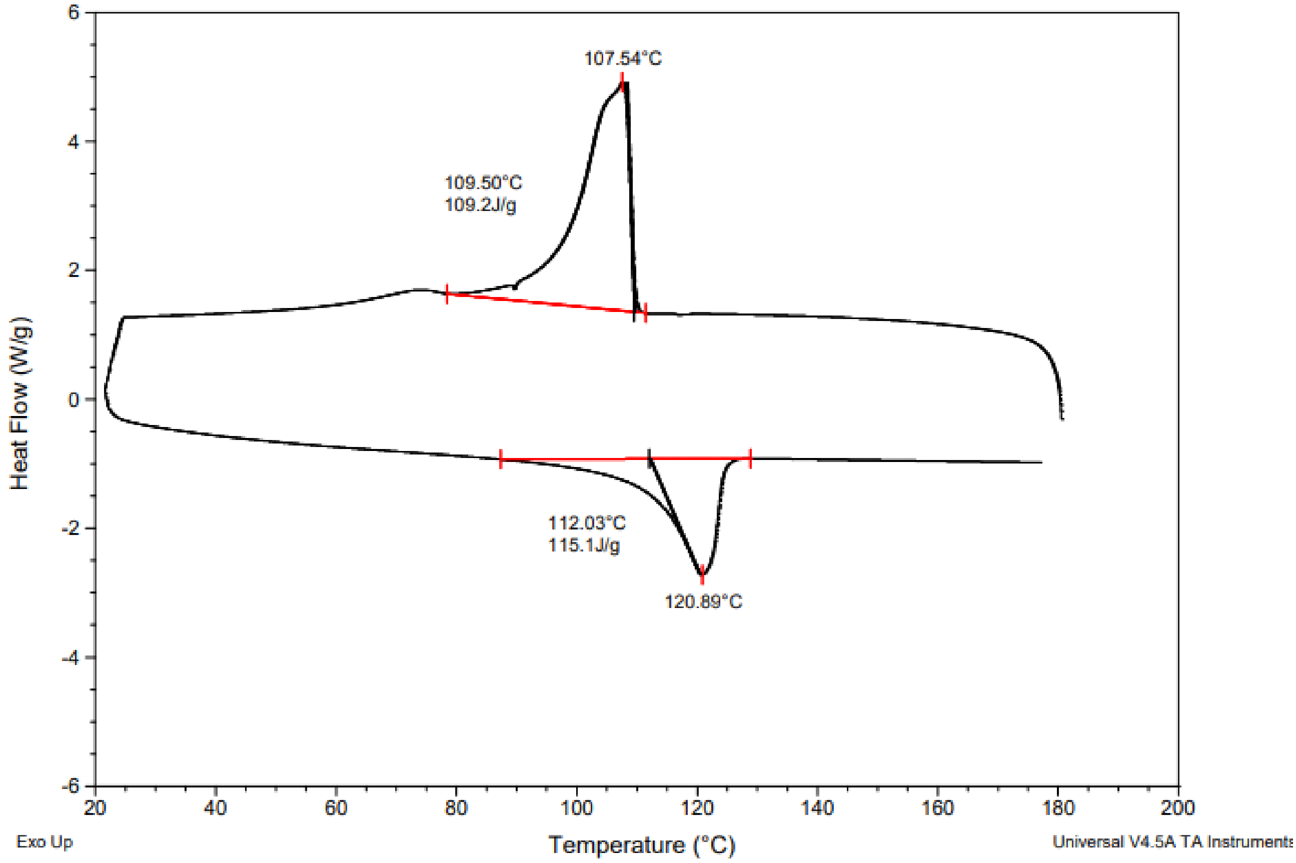

Figure S70. DSC data of the polymer from table 2, entry 3

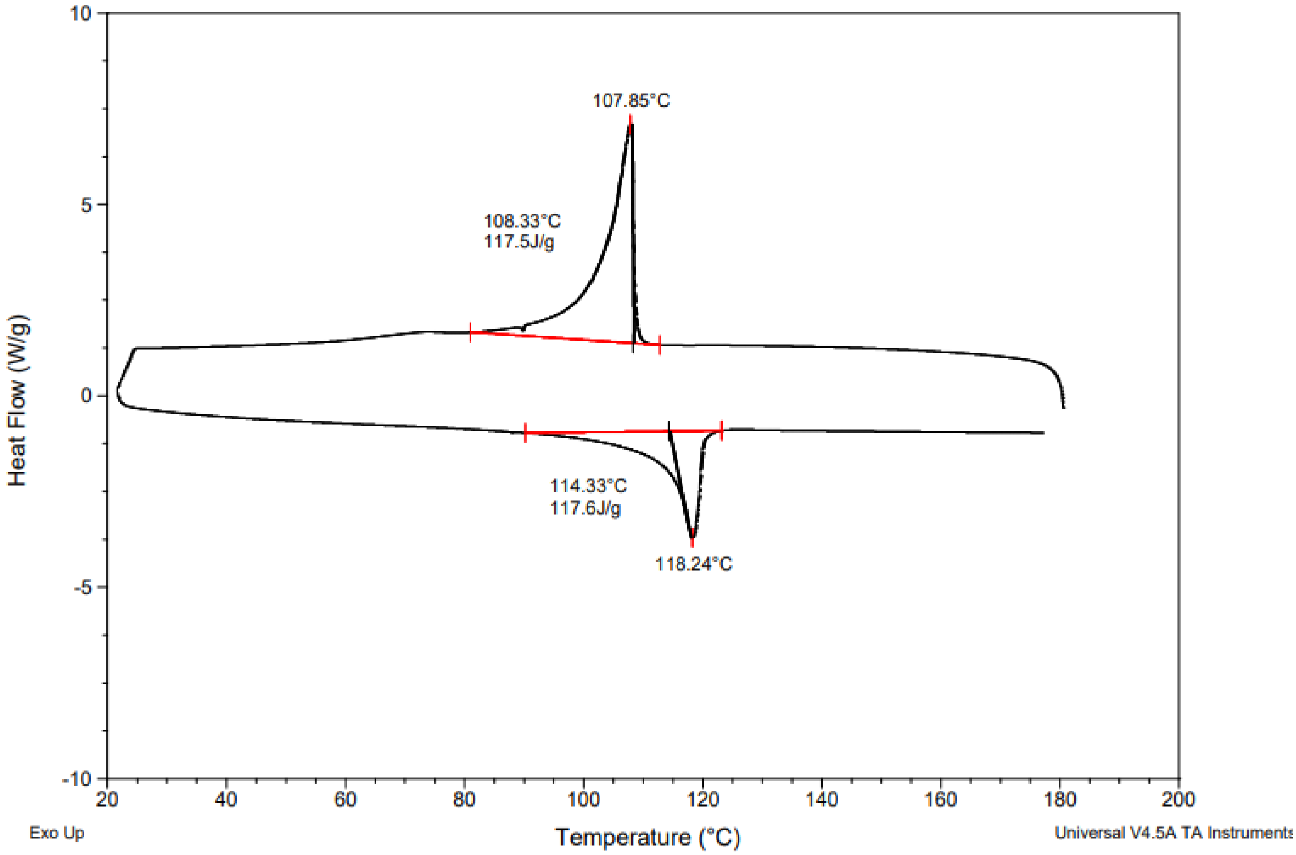

Figure S71. DSC data of the polymer from table 2, entry 4

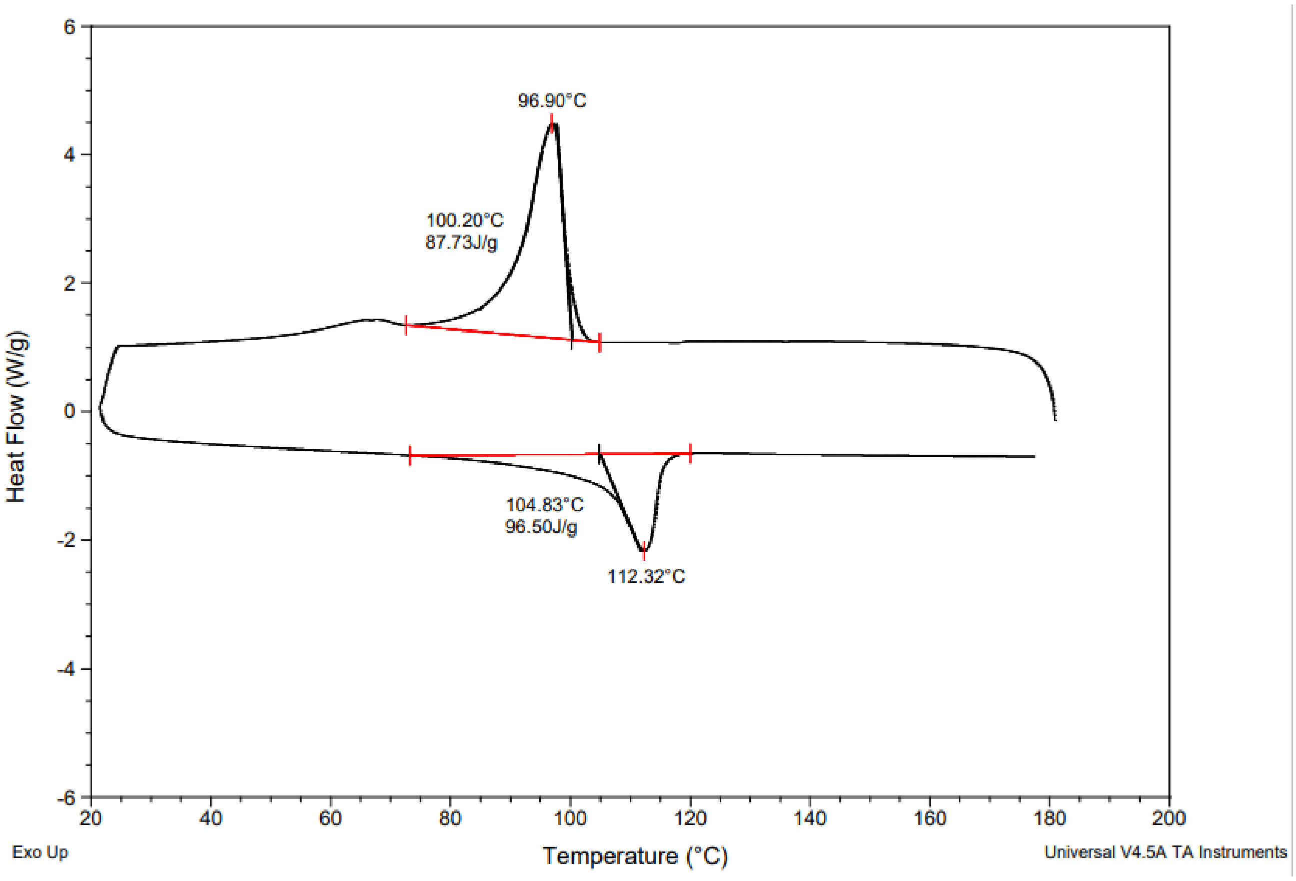

Figure S72. DSC data of the polymer from table 2, entry 5

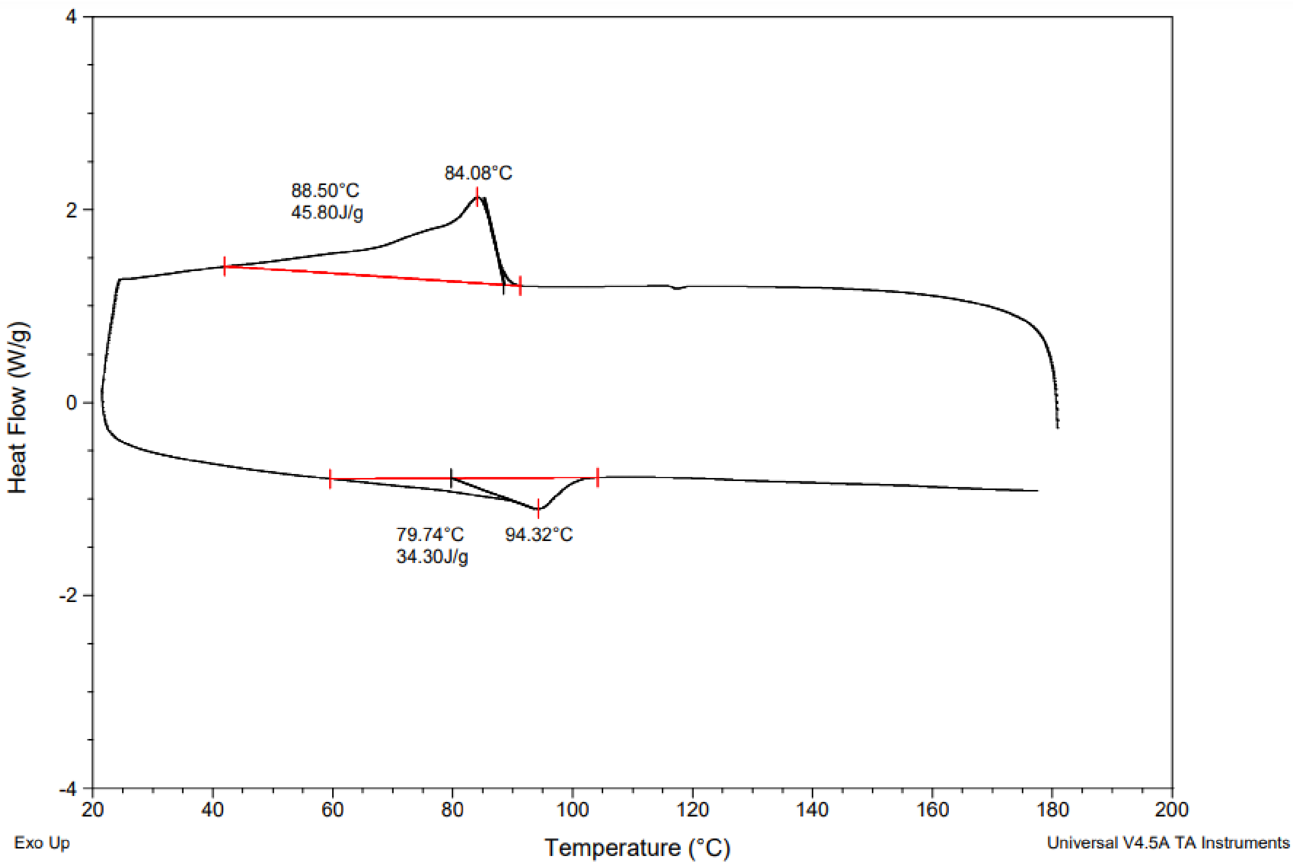

Figure S73. DSC data of the polymer from table 2, entry 7

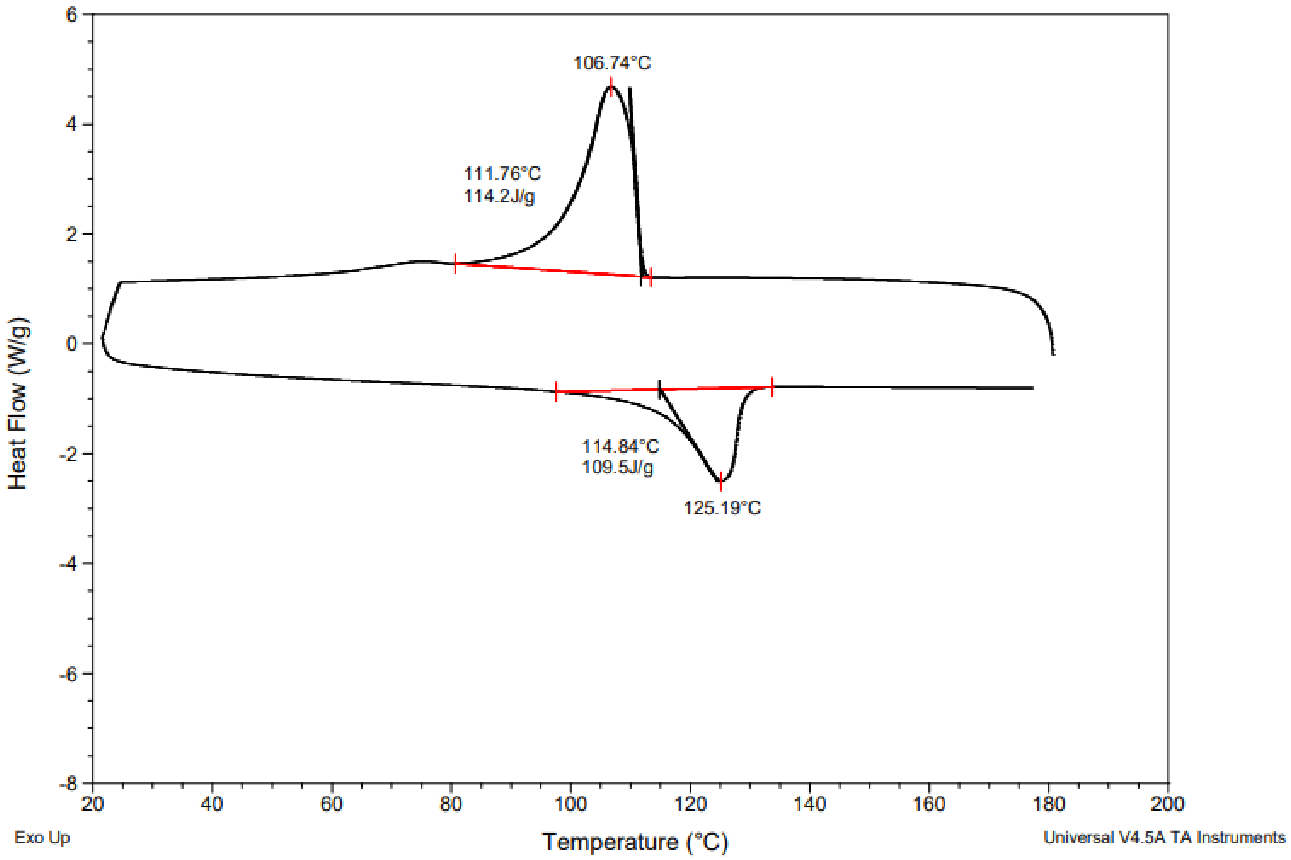

Figure S74. DSC data of the polymer from table 2, entry 9

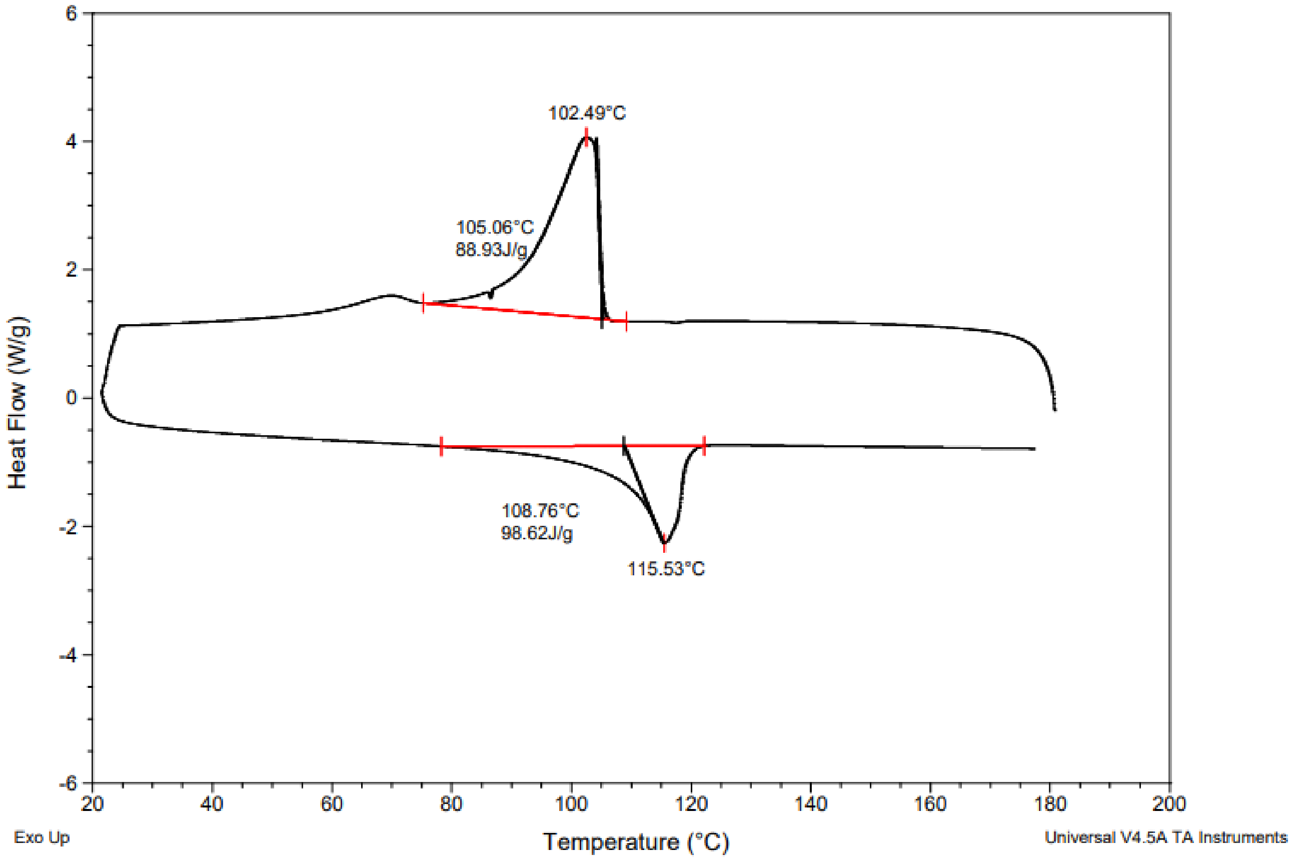

Figure S75. DSC data of the polymer from table 2, entry 10

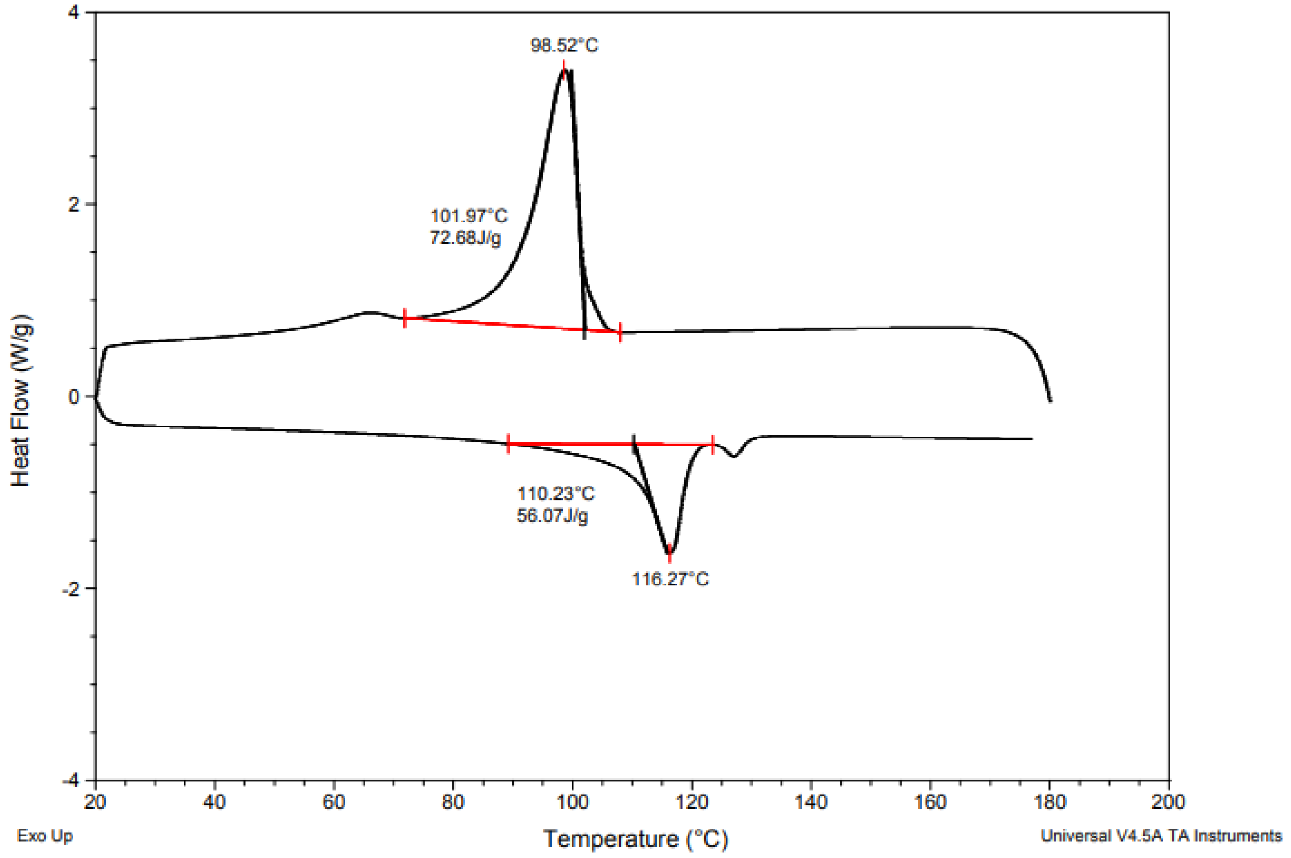

Figure S76. DSC data of the polymer from table 2, entry 11

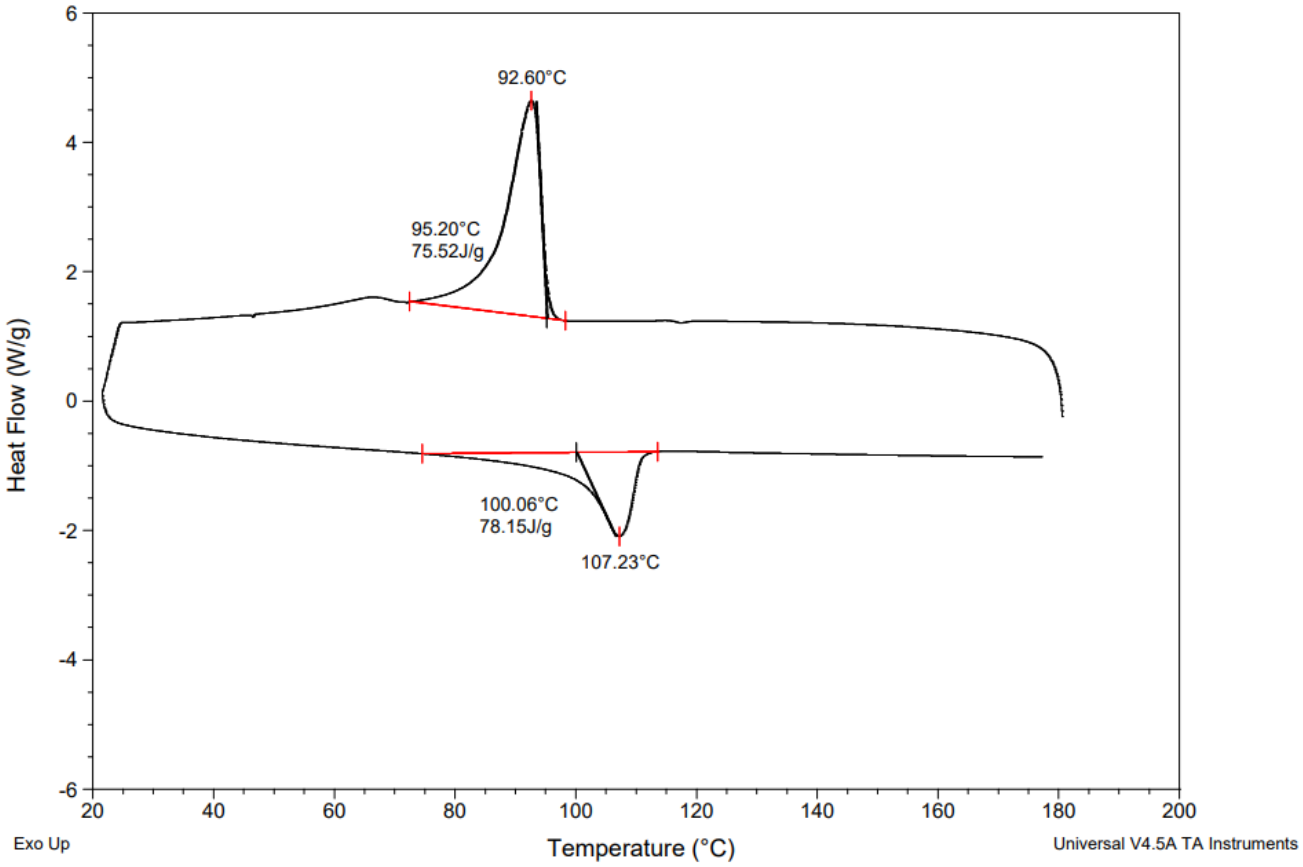

Figure S77. DSC data of the polymer from table 2, entry 12

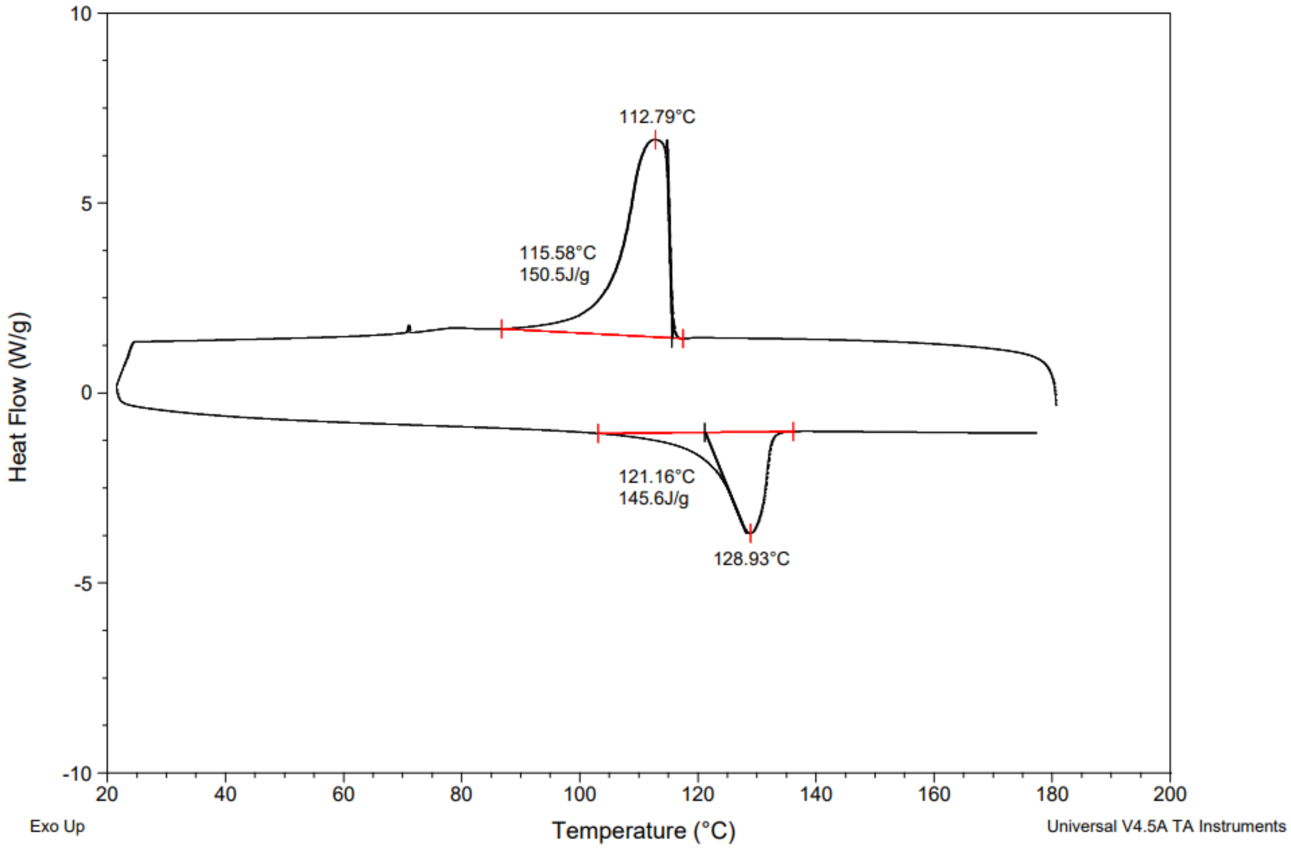

Figure S78. DSC data of the polymer from table 2, entry 13

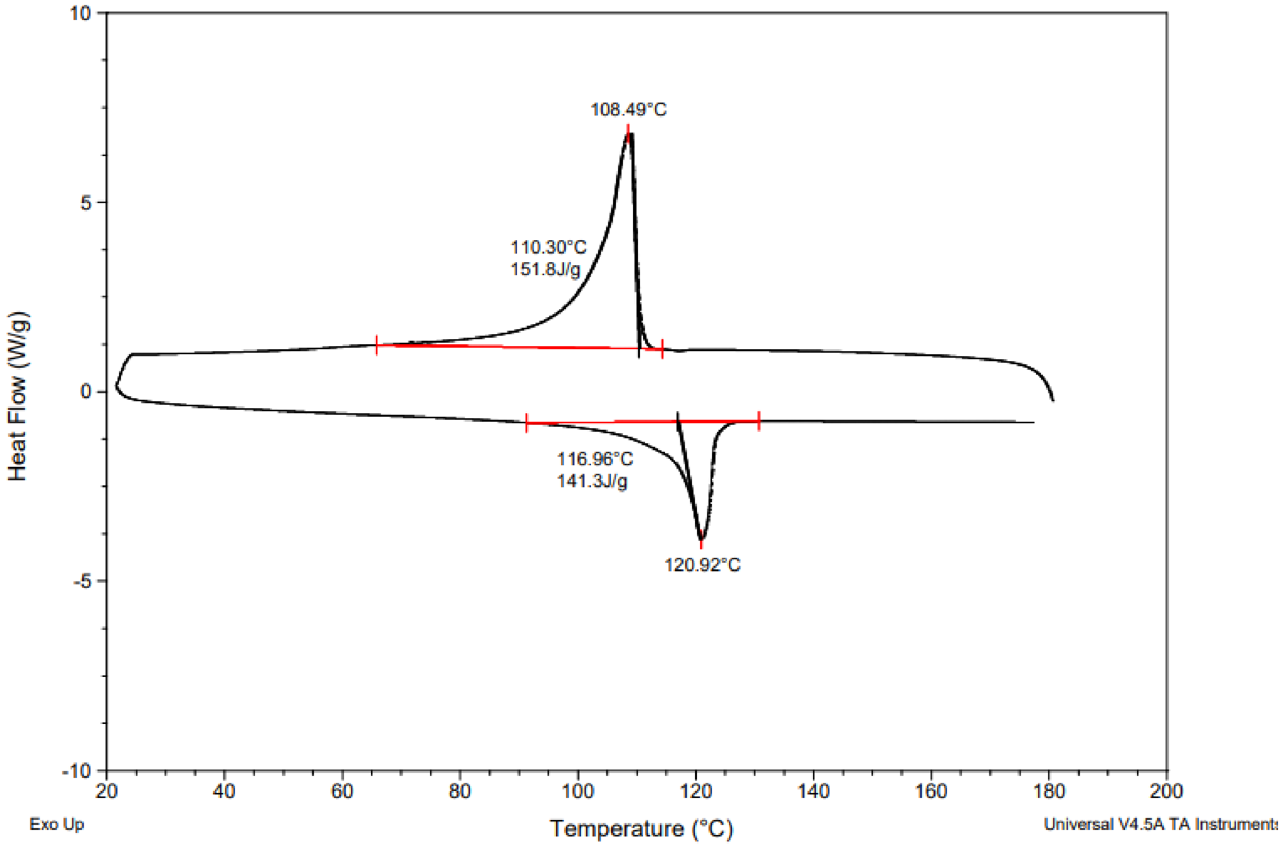

Figure S79. DSC data of the polymer from table 2, entry 14

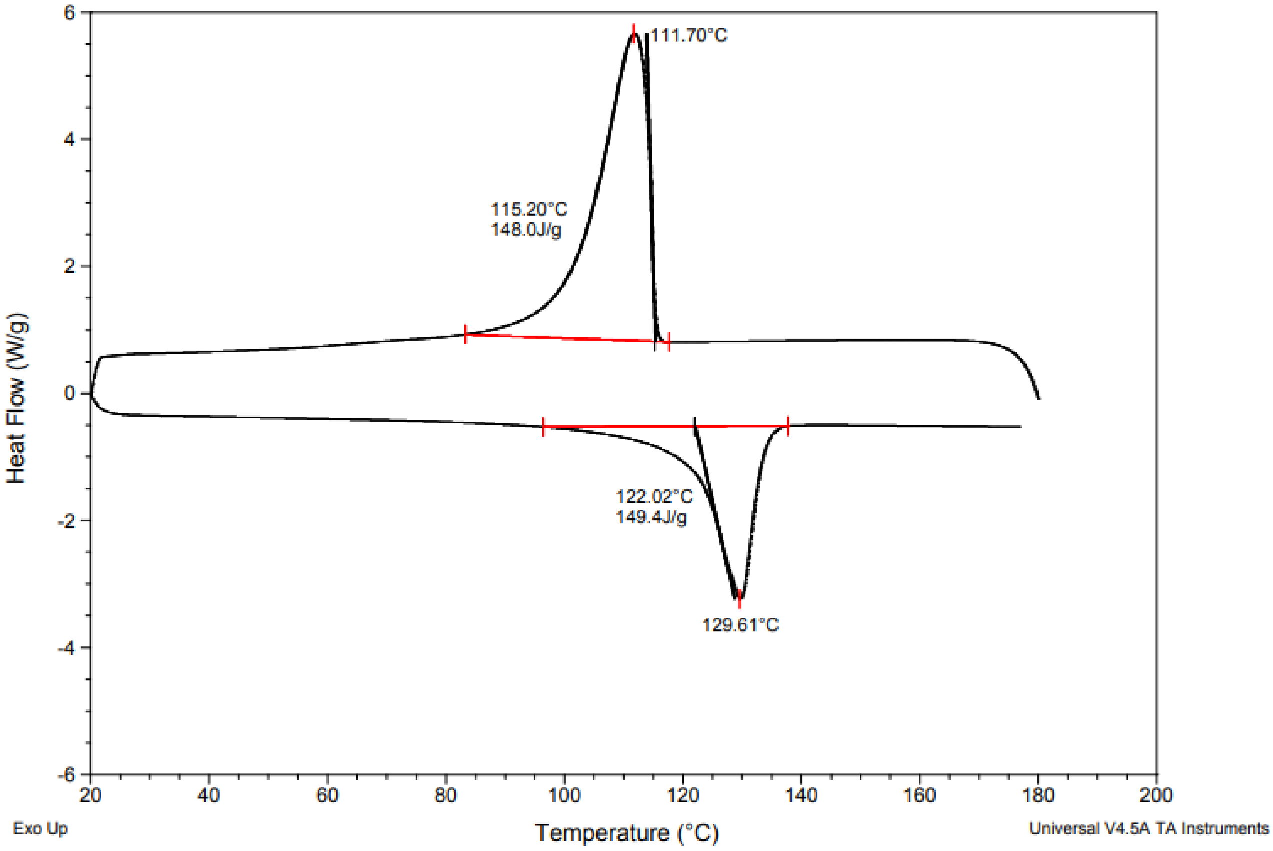

Figure S80. DSC data of the polymer from table 2, entry 15

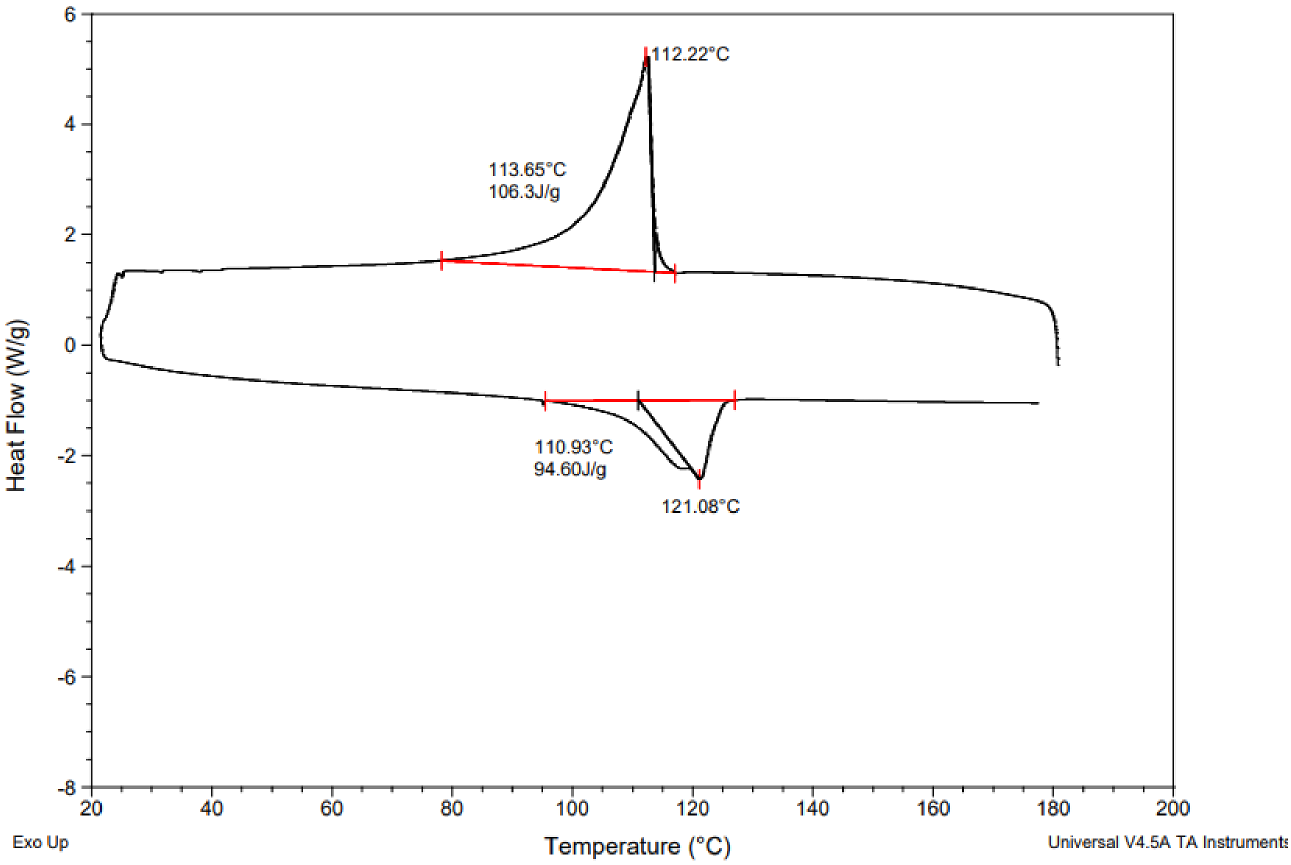

Figure S81. DSC data of the polymer from table 2, entry 16

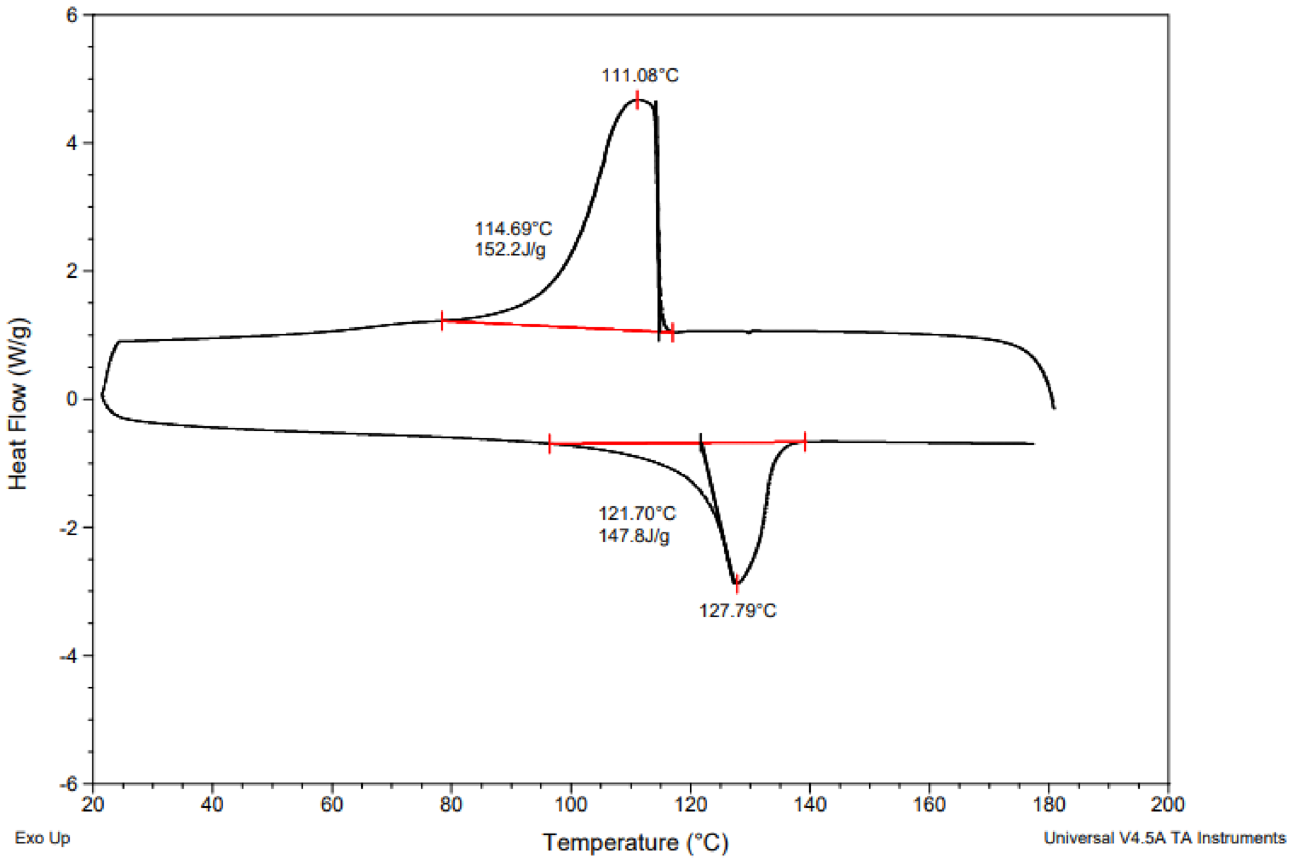

Figure S82. DSC data of the polymer from table 2, entry 17

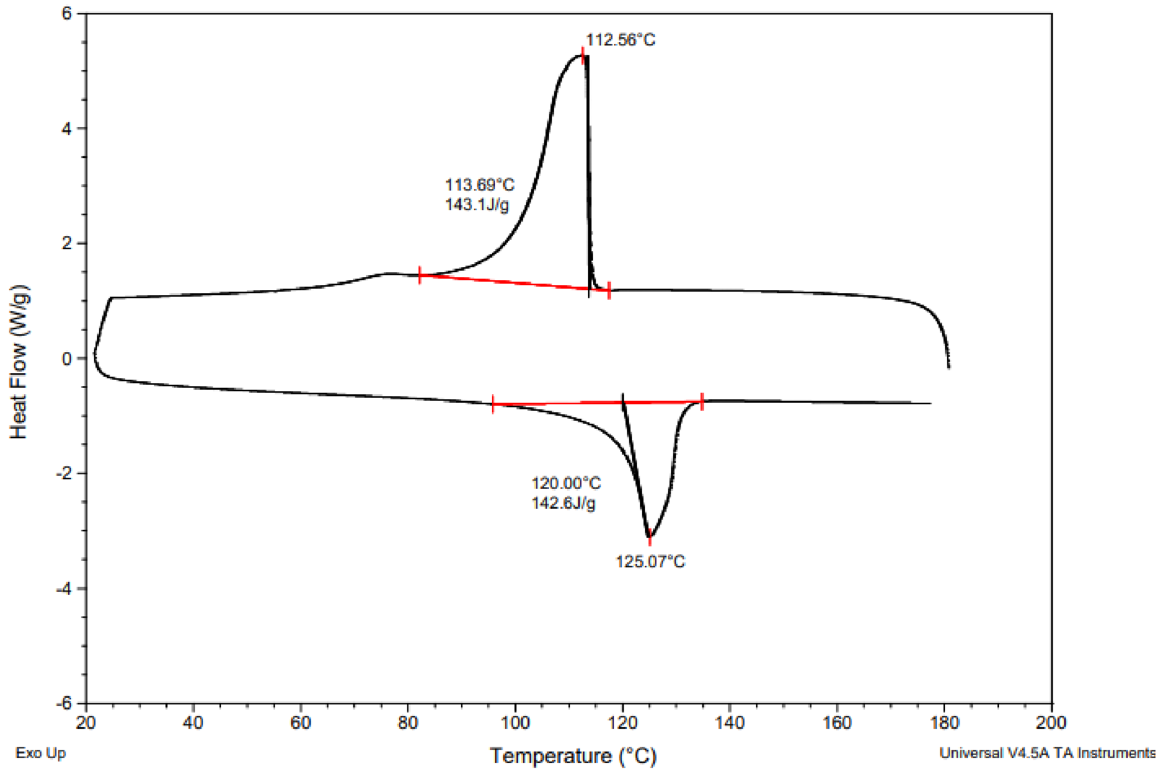

Figure S83. DSC data of the polymer from table 2, entry 18

6. IR spectra of copolymers

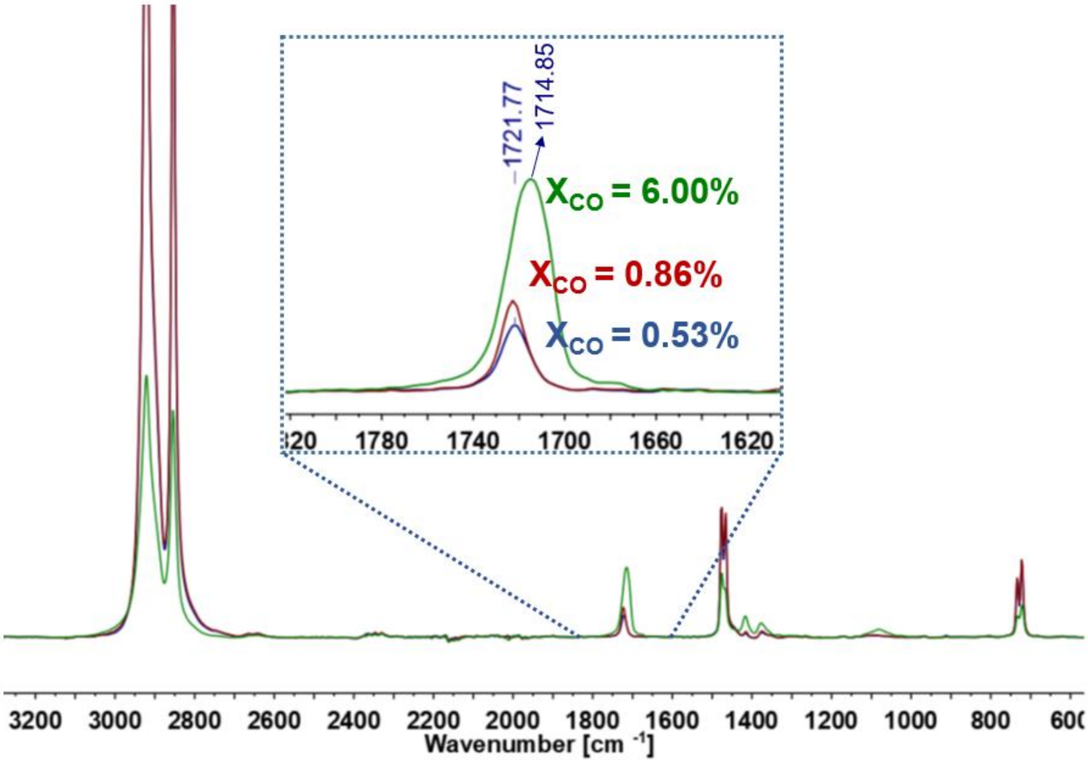

Figure S84. ATR-IR spectra of copolymers with different amounts of incorporated CO（**blue line**, table 1 entry 1; **red line**, table 1 entry 4; **green line**, table 1 entry 5;）

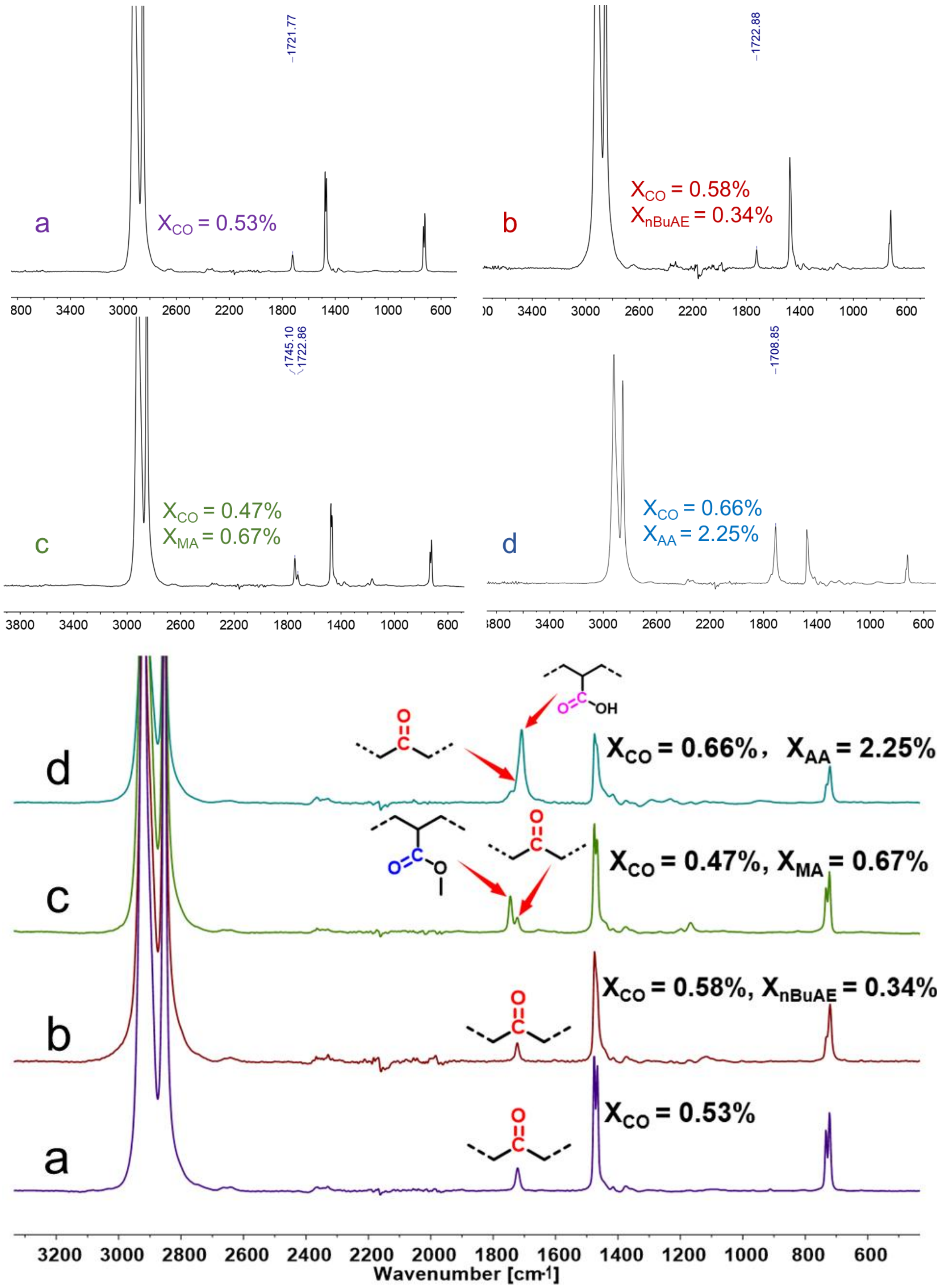

Figure S85. ATR-IR spectra of copolymers with different monomers. **a**, table 1 entry 1; **b**, table 2 entry 18; **c**, table 2 entry 2; **d**, table 2 entry 11;

7. Contact angle measurements

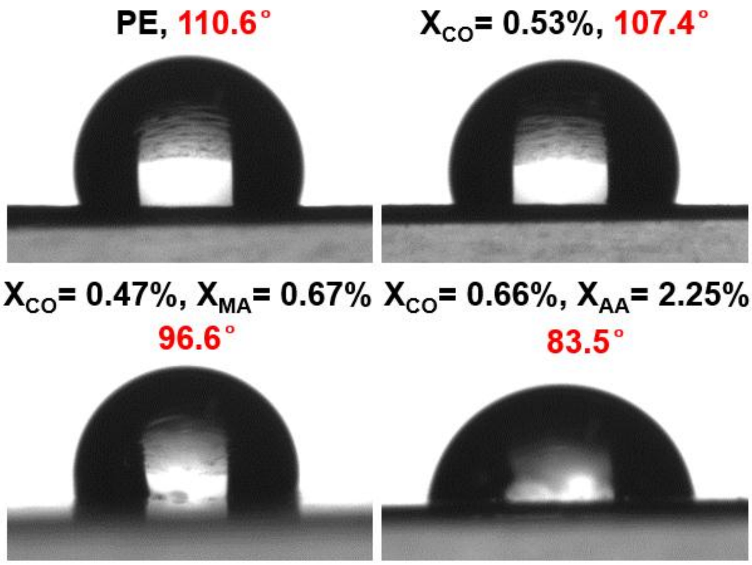

Figure S86. Drops of water on copolymers with different contents of comonomer

8. TGA data of polymers

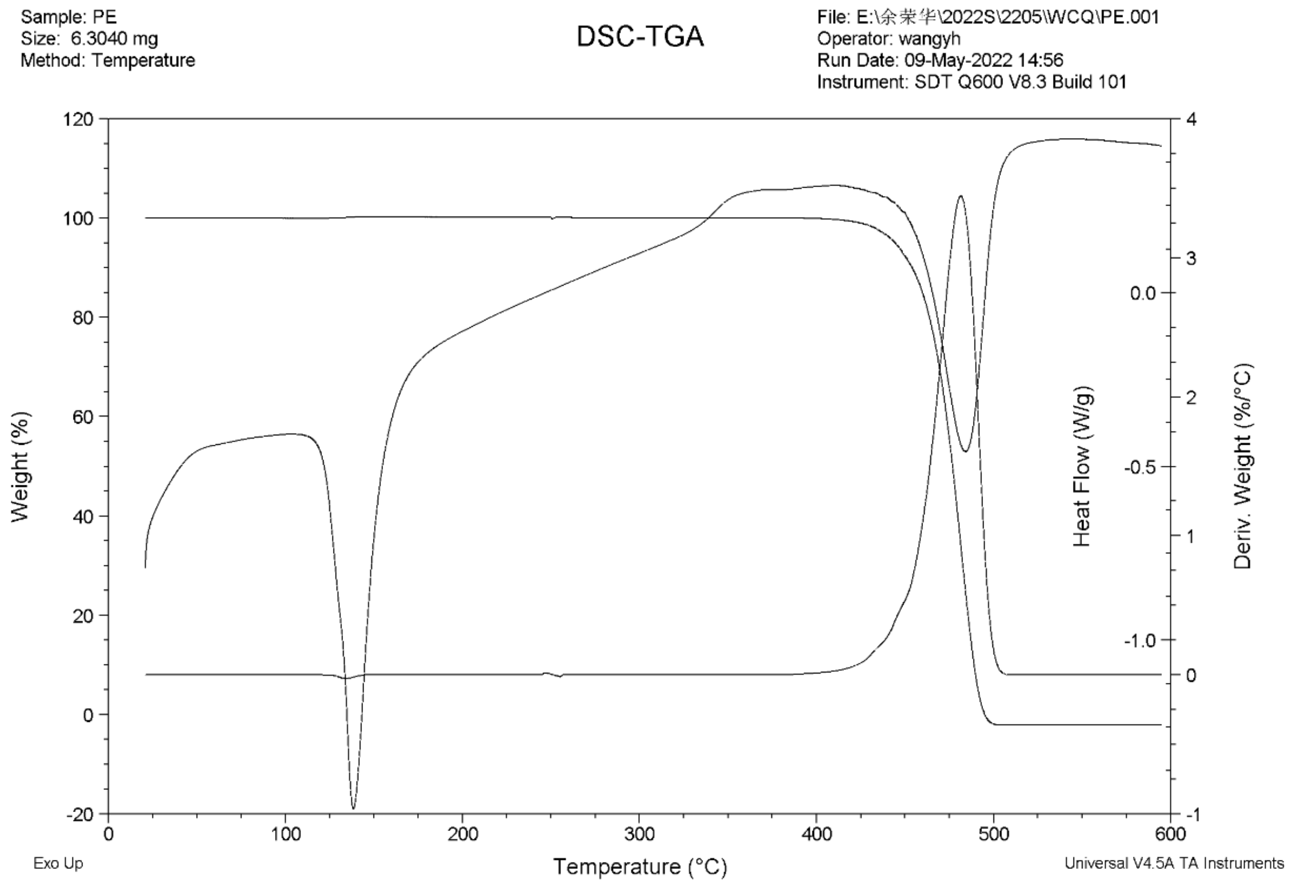

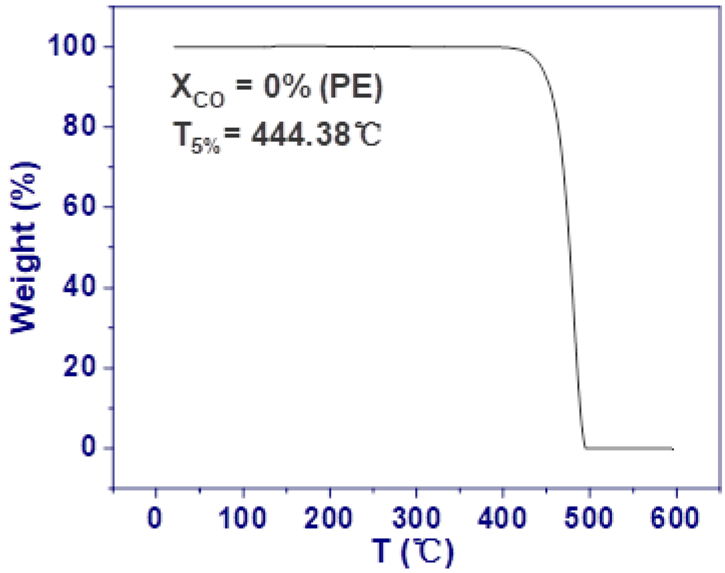

Figure S87. TGA trace of the polymer from table 1, entry 9

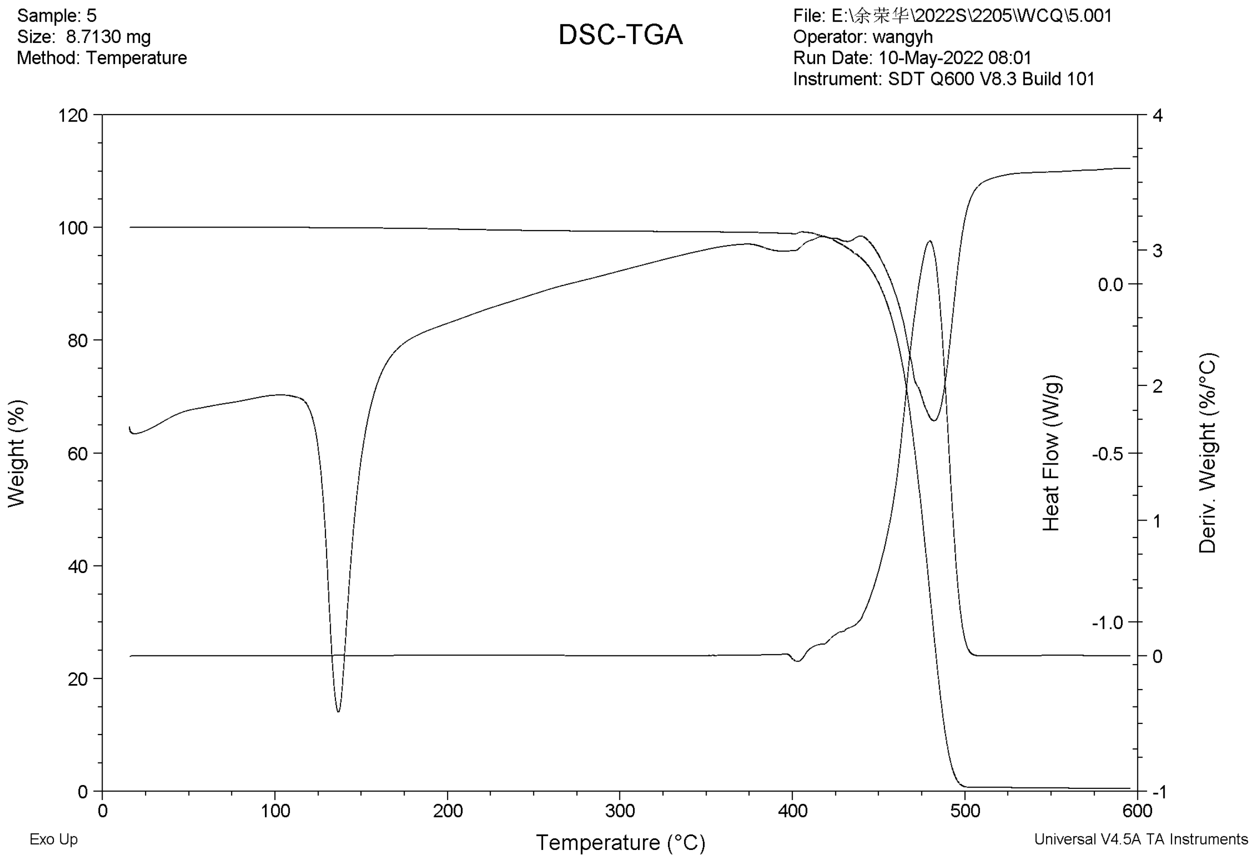

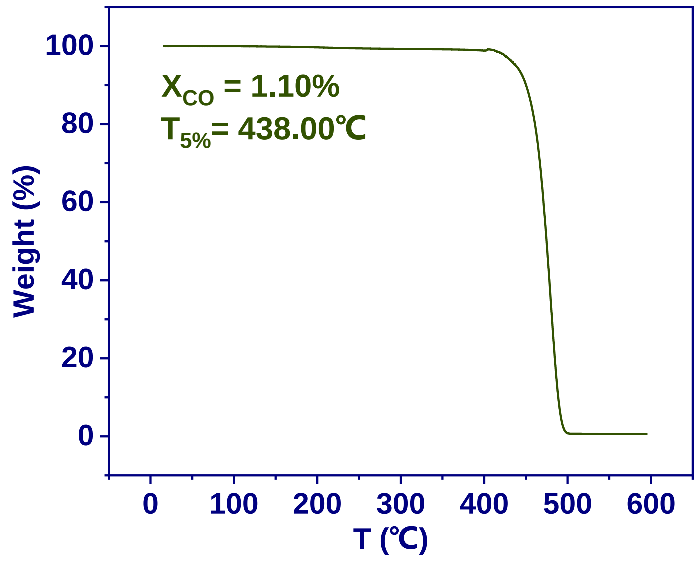

Figure S88. TGA trace of the polymer from table 1, entry 3

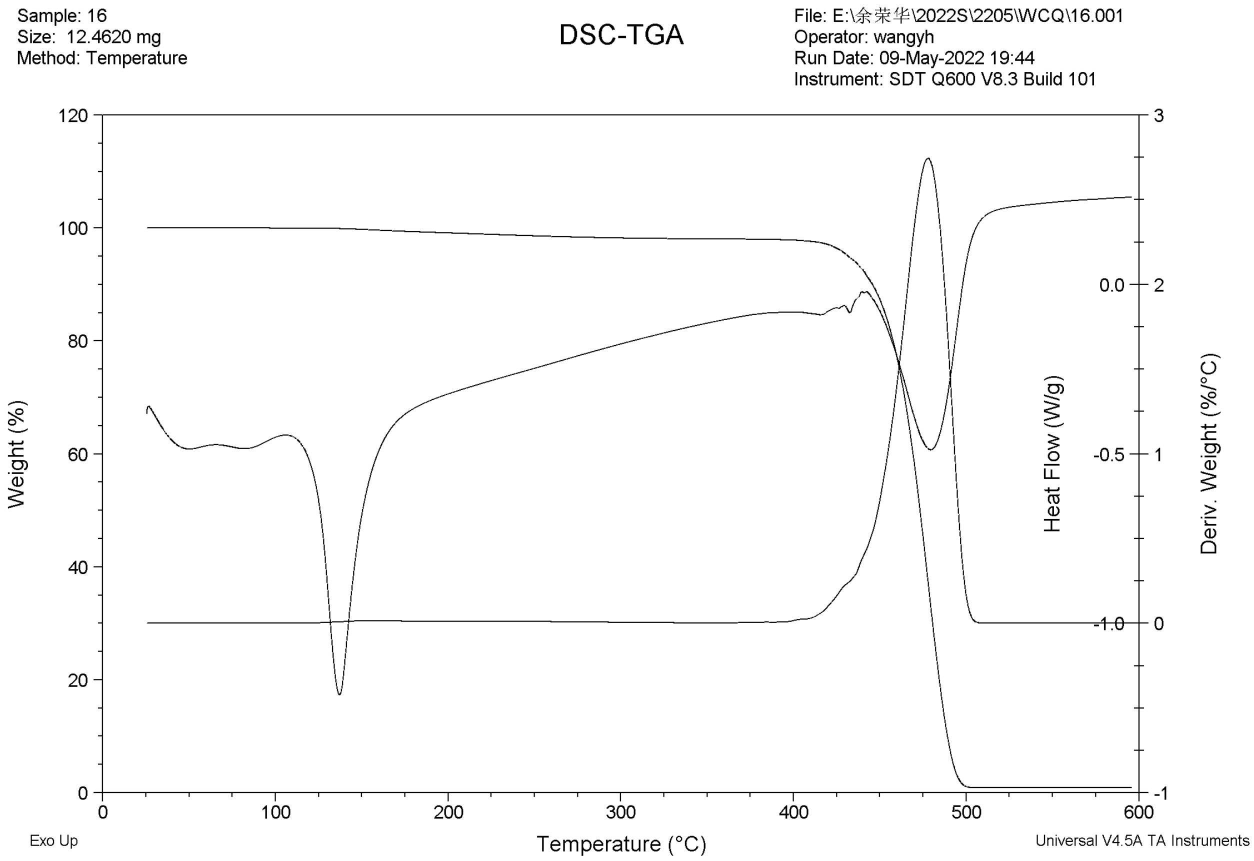

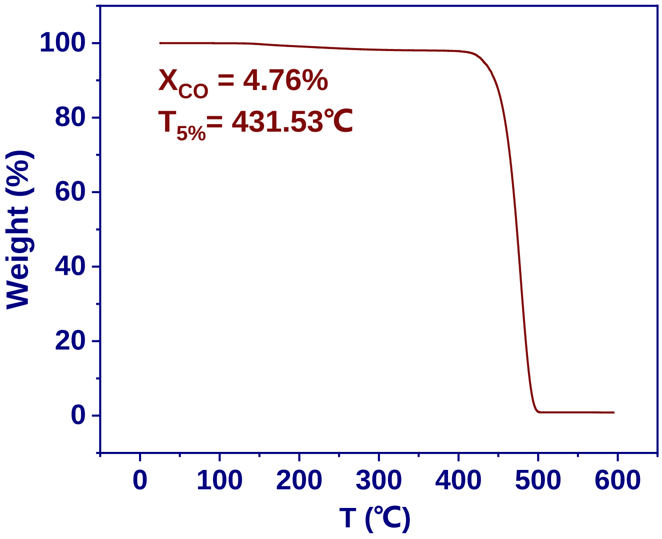

Figure S89. TGA trace of the polymer from table 1, entry 8

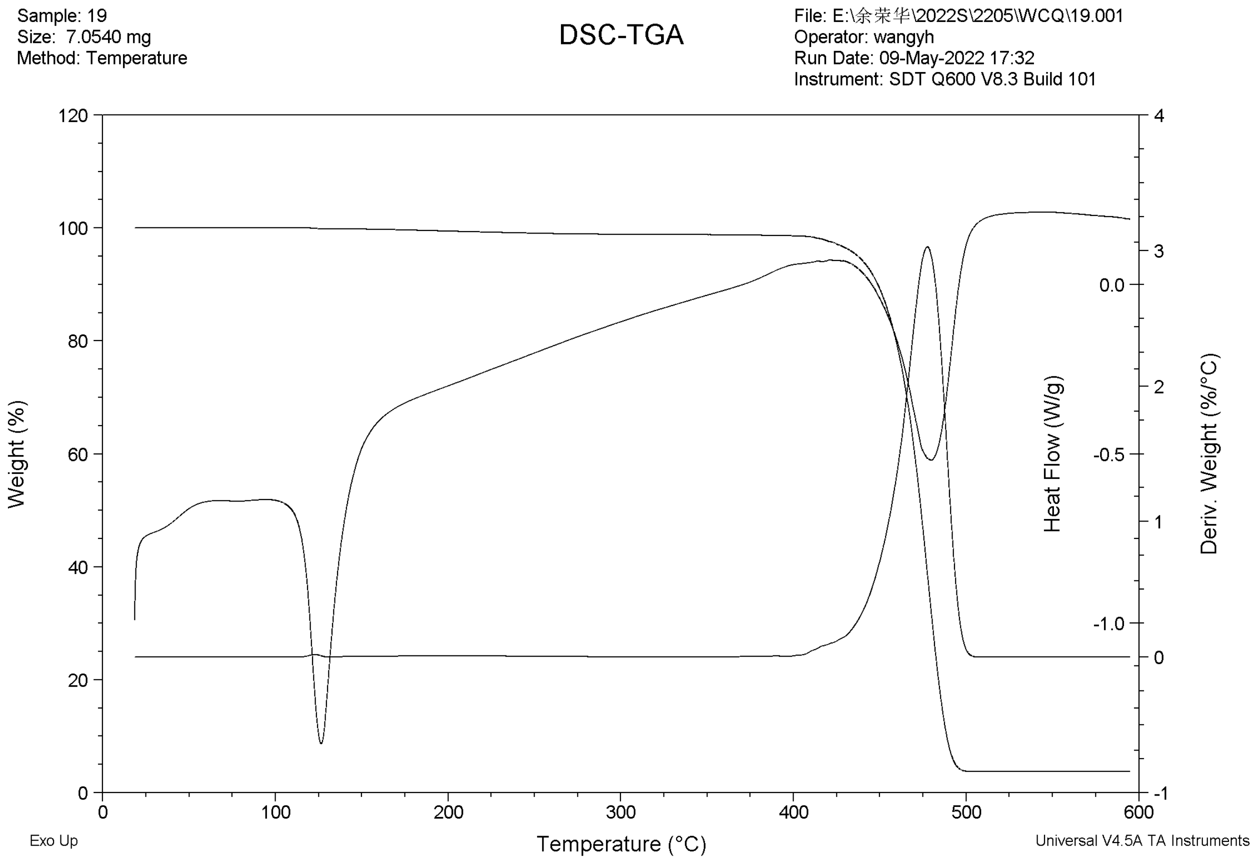

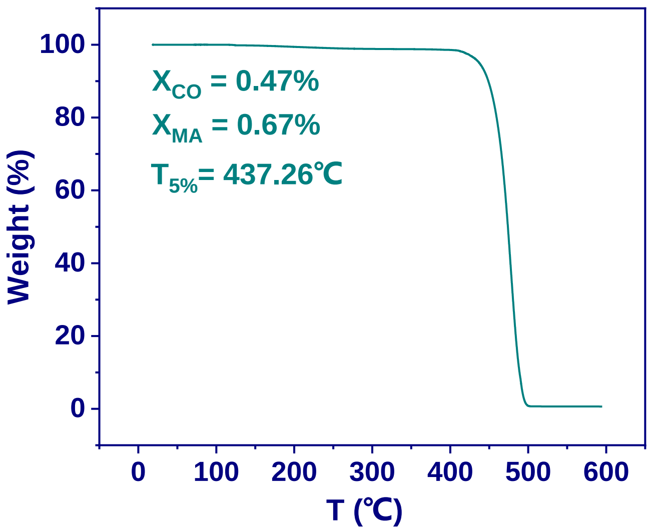

Figure S90. TGA trace of the polymer from table 2, entry 2

9. WXRD data of polymers

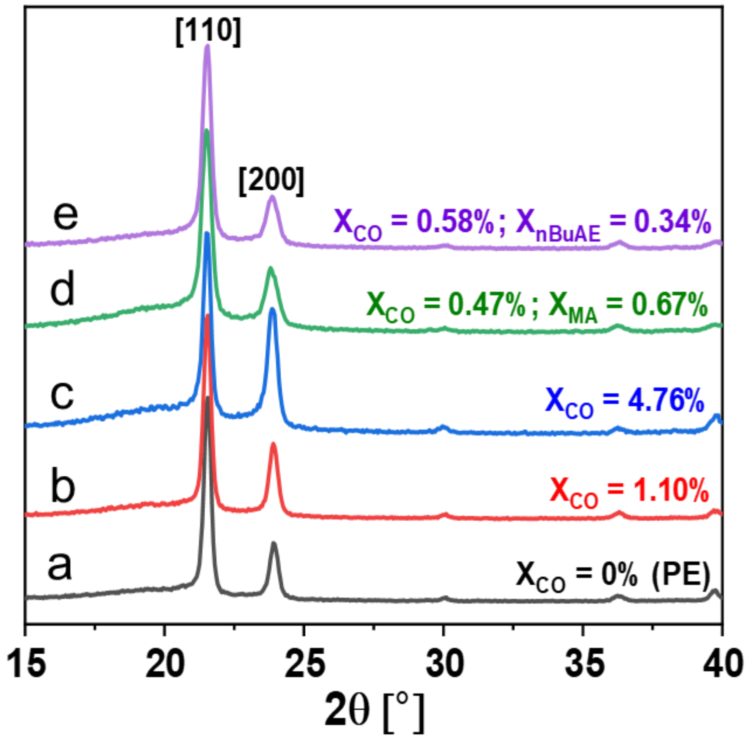

Figure S91. WXRD traces of polymers reflex in the 2θ range 15 – 40 °. (a) table 1, entry 9; (b) table 1, entry 3; (c) table 1, entry 8; (d) table 2, entry 2; (e) table 2, entry 18.

10. Tensile experiments

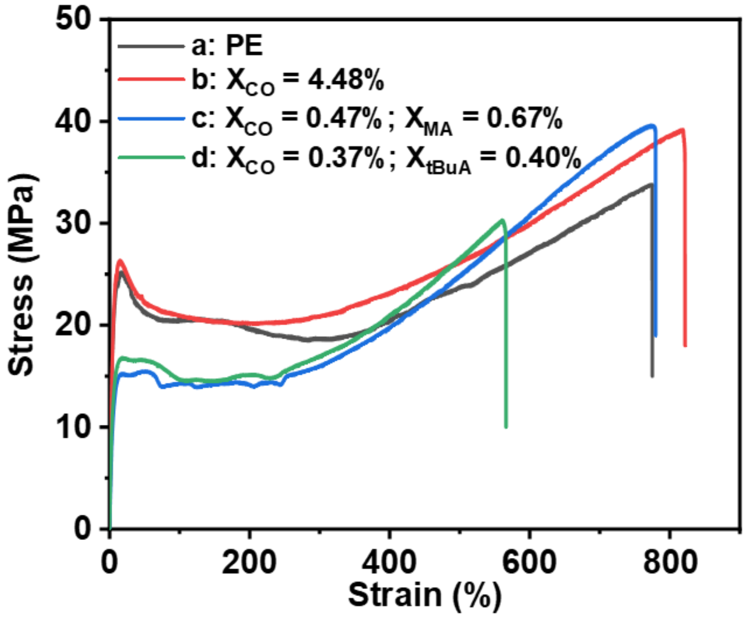

Figure S92. Stress-Strain curve of polymers. (a) Table 1, entry 9; (b) Table 1, entry 7; (c) Table 2, entry 2; (d) Table 2, entry 9.

11. Photodegradation experiment

The UV lamp (powered by a 19 V/3.4 A power supply) used in the photodegradation experiment is shown in the figure below. The front of the lamp is two rows of small LED lamps (12 each), the light wavelengths are 275nm and 255nm respectively, and the radiation density is 12 mW/cm^2. The back of the lamp is a row of aluminum alloy plates for heat dissipation. At 5cm directly below the UV lamp, the film samples (2.5cm×1cm×0.15mm) were irradiated for 2 d at 30 ℃. Molecular weight and molecular weight distribution are measured by GPC.

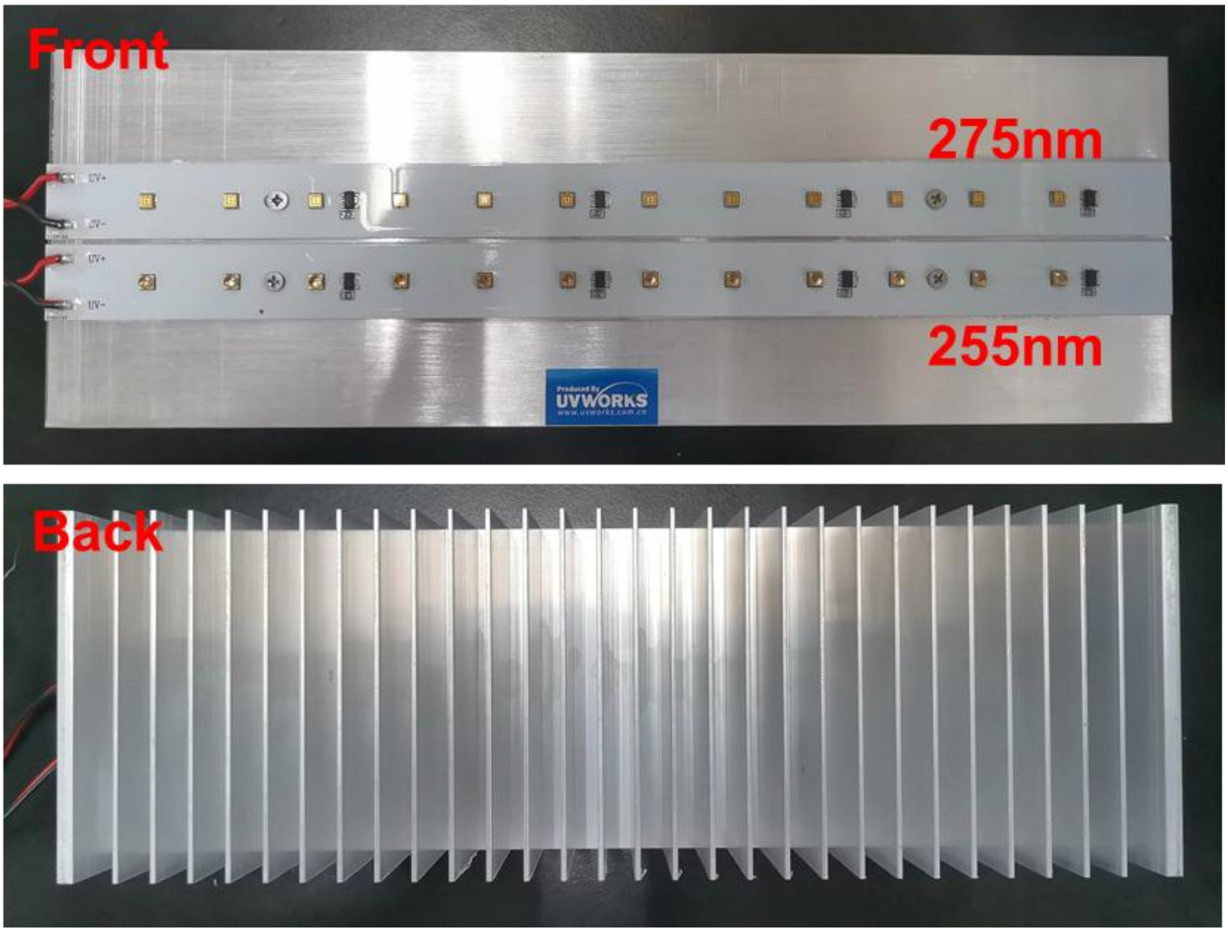

Figure S93. UV lamp

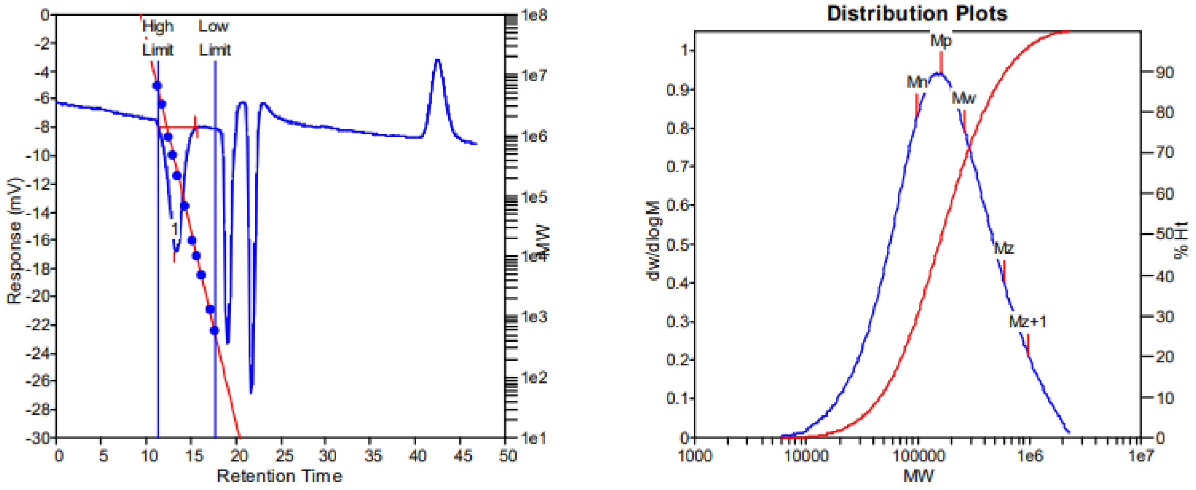

MW Averages

Peak No	Mp	Mn	Mw	Mz	Mz+1	Mv	PD
1	159506	99745	263985	591952	980976	229946	2.6466

Processed Peaks

Peak No	Name	Start RT (mins)	Max RT (mins)	End RT (mins)	Pk Height (mV)	% Height	Area (mV.secs)	% Area
1		11.42	13.27	15.55	-8.78596	100	896.086	100

Figure S94. GPC trace of the polymer (table 1, entry 9) after UV irradiation (275nm and 255nm at 30 ℃ for 2 d).

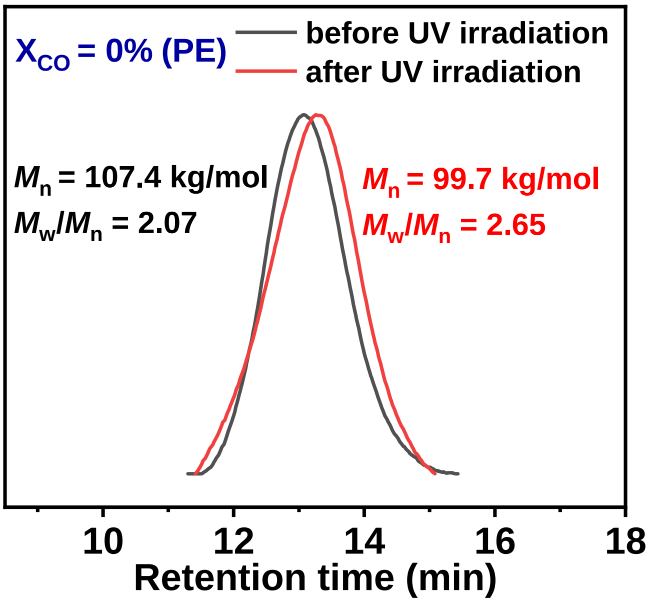

Figure S95. GPC traces of the polymer (table 1, entry 9) before and after UV irradiation (275 nm and 255 nm at 30 ℃ for 2 d).

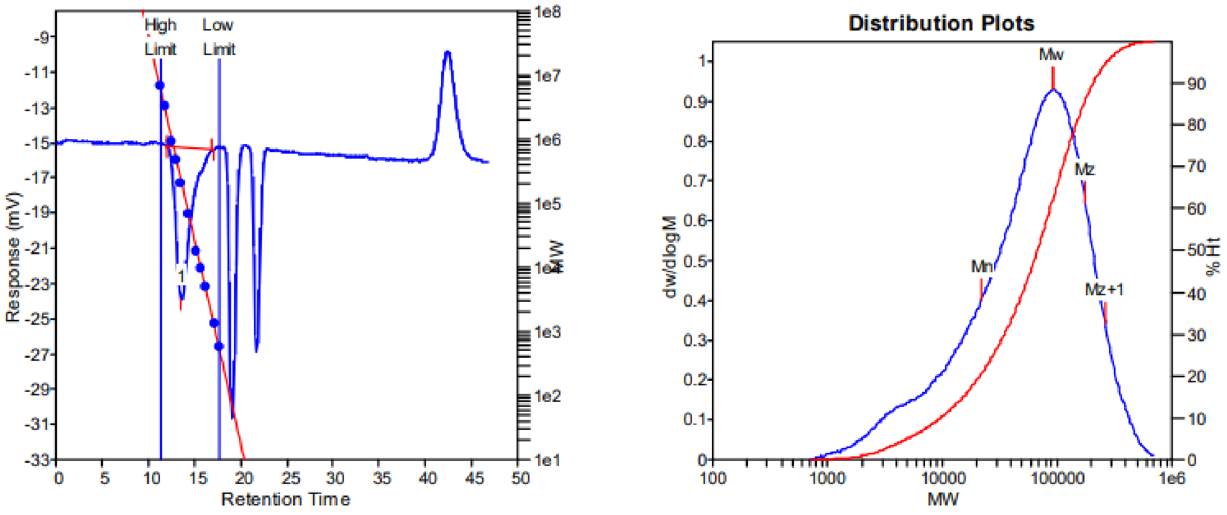

MW Averages

Peak No	Mp	Mn	Mw	Mz	Mz+1	Mv	PD
1	91833	21957	91553	177806	263384	81326	4.16965

Processed Peaks

Peak No	Name	Start RT (mins)	Max RT (mins)	End RT (mins)	Pk Height (mV)	% Height	Area (mV.secs)	% Area
1		12.12	13.53	16.95	-8.66075	100	907.475	100

Figure S96. GPC trace of the polymer (table 1, entry 3) after UV irradiation (275 nm and 255 nm at 30 ℃ for 2 d).

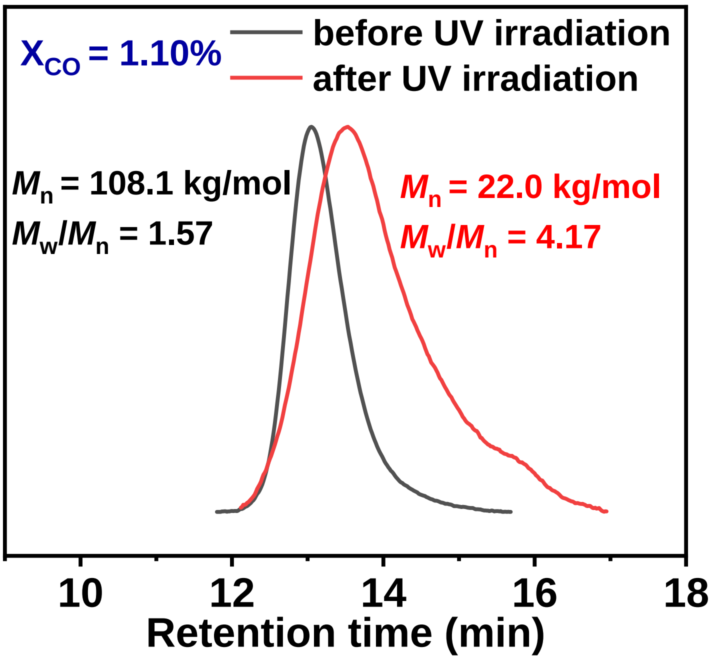

Figure S97. GPC traces of the polymer (table 1, entry 3) before and after UV irradiation (275 nm and 255 nm at 30 ℃ for 2 d).

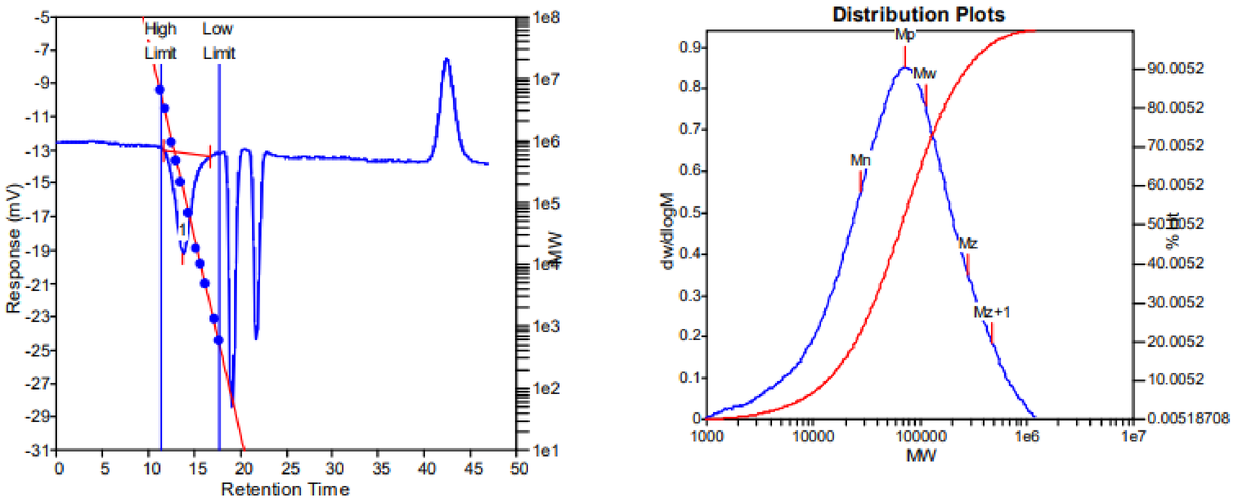

MW Averages

Peak No	Mp	Mn	Mw	Mz	Mz+1	Mv	PD
1	72487	27904	113321	282130	482816	97555	4.0611

Processed Peaks

Peak No	Name	Start RT (mins)	Max RT (mins)	End RT (mins)	Pk Height (mV)	% Height	Area (mV.secs)	% Area
1		11.72	13.70	16.77	-6.01832	100	686.477	100

Figure S98. GPC trace of the polymer (table 2, entry 2) after UV irradiation (275 nm and 255 nm at 30 ℃ for 2 d).

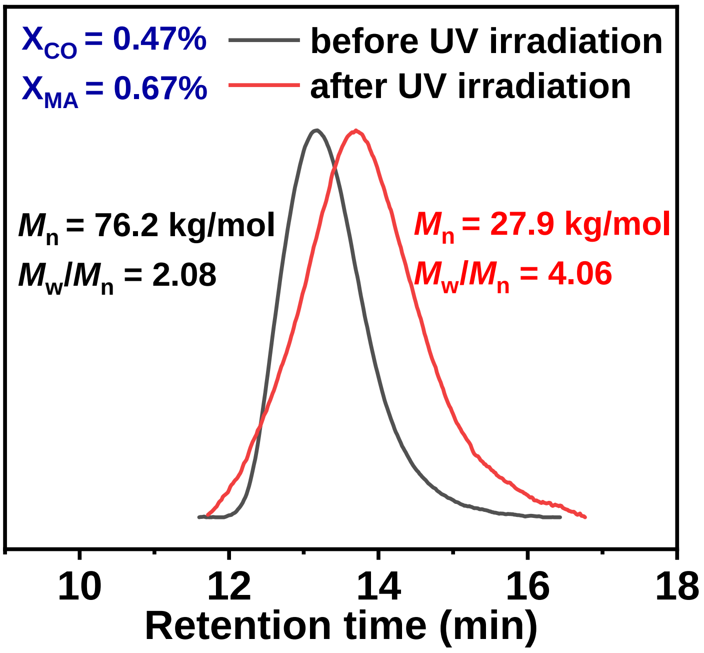

Figure S99. GPC traces of the polymer (table 2, entry 2) before and after UV irradiation (275 nm and 255 nm at 30 ℃ for 2 d).

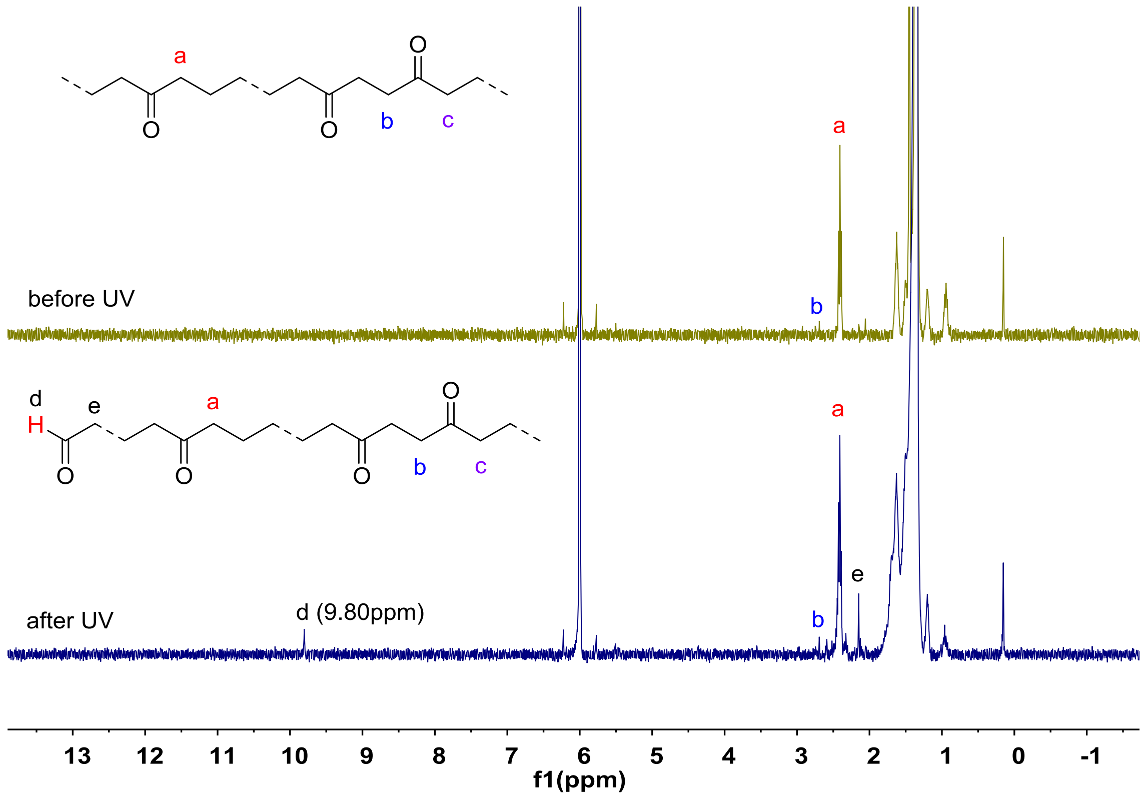

Figure S100. ^{1}H NMR spectra (400 MHz, $C_2D_2Cl_4$, 110 ℃) of the polymer (table 1, entry 3) before and after UV irradiation (275 nm and 255 nm at 30 ℃ for 2 d).

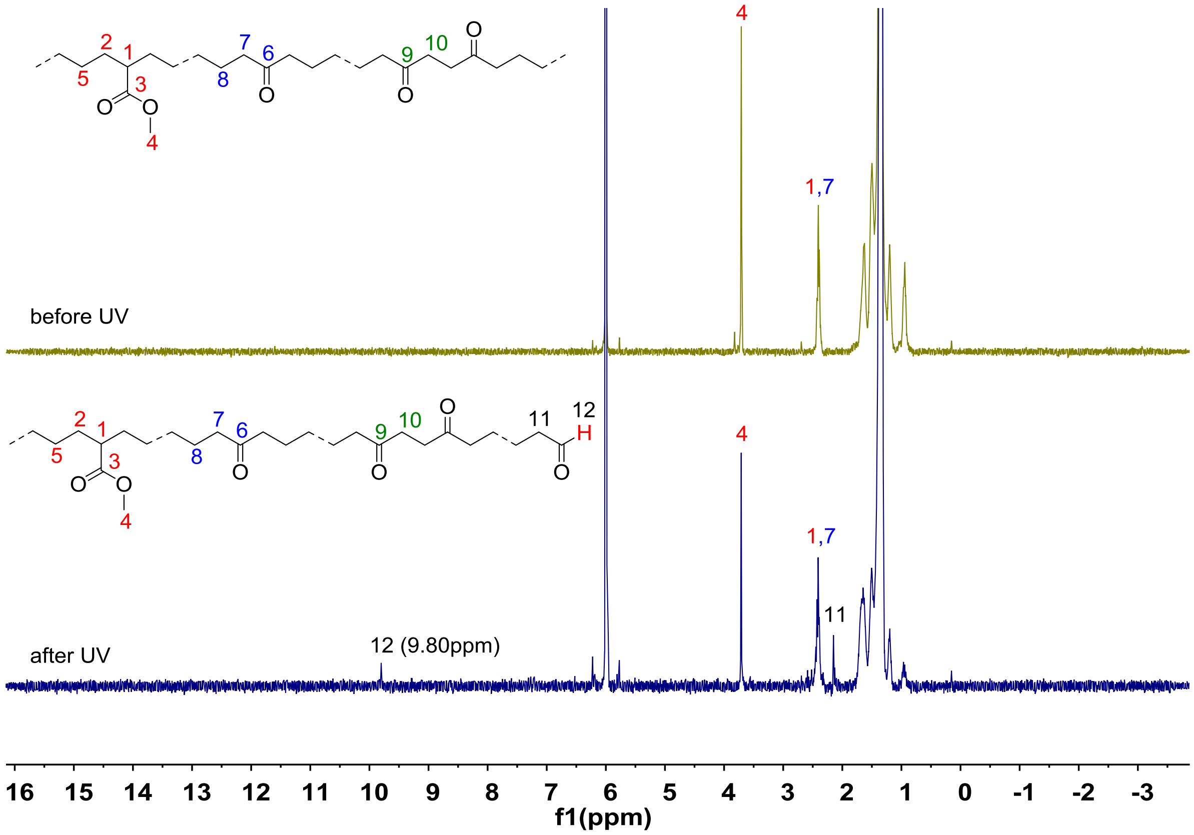

Figure S101. ^{1}H NMR spectra (400 MHz, $C_2D_2Cl_4$, 110 ℃) of the polymer (table 2, entry 2) before and after UV irradiation (275 nm and 255 nm at 30 ℃ for 2 d).

12. Crystallographic Data

	**Pd4**
Formula	$C_{24}H_{35}O_7PPdS_2$
Formula weight	637.01
Crystal dimensions (mm^3)	0.5 × 0.3 × 0.01
Crystal system	triclinic
Space group	P -1
a (Å)	8.717(4)
b (Å)	10.432(5)
c (Å)	16.095(8)
α (°)	99.007(14)
β (°)	100.775(14)
γ (°)	104.424(14)
Volume ($Å^3$)	1360.3(11)
Z	2
T (K)	294.8
D_{calcd} (g cm^{-3})	1.555
μ (mm^{-1})	0.935
F (000)	656
No. of rflns. collected	36639
No. of indep. rflns. /R_{int}	7840 / 0.0420
No. of obsd. rflns. [$I_0 > 2\sigma(I_0)$]	5634
Data / restraints / parameters	7840 / 48 / 361
R_1 / wR_2 [$I_0 > 2\sigma(I_0)$]	0.0387 / 0.0825
R_1 / wR_2 (all data)	0.0681 / 0.0912
GOF (on F^2)	1.035
Largest diff. peak and hole (e $Å^{-3}$)	0.909 / -0.714
CCDC No.	2177581

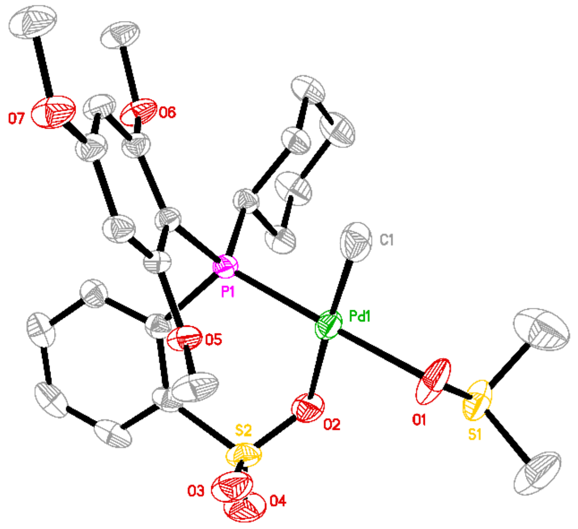

Figure S102. Molecular structure of Pd4. It is drawn with 30% probability ellipsoids and hydrogen atoms are omitted for clarity.

13. References

[1] SMART, version 5.054; Bruker AXS Inc.: Madison, WI, **2000**.

[2] SAINT and SADABS, version 6.22; Bruker AXS Inc.: Madison, WI, **2000**.

[3] (a) G. M. Sheldrick, *Acta Cryst.* **2015**, *C71*, 3-8. (b) O. V. Dolomanov, L. J. Bourhis, R. J. Gildea, J. A. K. Howard, H. Puschmann, *J. Appl. Cryst.* **2009**, *42*, 339-341.

[4] D. Guironnet, P. Roesle, T. Rünzi, I. Göttker-Schnetmann, S. Mecking, *J. Am. Chem. Soc.*, **2009**, *131*, 422–423.

[5] T. Kochi, S. Noda, K. Yoshimura, k. Nozaki, *J. Am. Chem. Soc.* **2007**, 129, 8948− 8949.

[6] J. Gao, W. Cai, Y. Hu, C. Chen, *Polym. Chem.* **2019**, 10, 1416–1422.

[7] J. Xia, Y.-F. Han, S. Kou, Y. Zhang, Z. Jian, *European Polymer Journal* **2021**, *160*, 110781.

[8] Y. Zhang; C. Wang; S. Mecking; Z. Jian, *Angew. Chem. Int. Ed.* **2020**, *59*, 14296−14302.

[9] S. Dai, X. Sui, C. Chen, *Angew. Chem. Int. Ed.* **2015**, *54*, 9948-9953.
