# Supplementary material for: Photodegradable polar-functionalized polyethylenes
Source: Natl Sci Rev. 2023 Feb 16;10(7):nwad039. doi: 10.1093/nsr/nwad039 (PMC10434297; doi:10.1093/nsr/nwad039)

checkCIF/PLATON report

Structure factors have been supplied for datablock(s) mo_22jun06wtd11_0m

THIS REPORT IS FOR GUIDANCE ONLY. IF USED AS PART OF A REVIEW PROCEDURE FOR PUBLICATION, IT SHOULD NOT REPLACE THE EXPERTISE OF AN EXPERIENCED CRYSTALLOGRAPHIC REFEREE.

No syntax errors found. CIF dictionary Interpreting this report

Datablock: mo_22jun06wtd11_0m

```
Bond precision:   C-C = 0.0041 A          Wavelength=0.71073

Cell:          a=8.717(4)         b=10.432(5)        c=16.095(8)
               alpha=99.007(14)   beta=100.775(14)   gamma=104.424(14)
Temperature:   295 K
```

	Calculated	Reported
Volume	1360.4(11)	1360.3(11)
Space group	P -1	P -1
Hall group	-P 1	-P 1
Moiety formula	C24 H35 O7 P Pd S2	C24 H35 O7 P Pd S2
Sum formula	C24 H35 O7 P Pd S2	C24 H35 O7 P Pd S2
Mr	637.01	637.01
Dx,g cm-3	1.555	1.555
Z	2	2
Mu (mm-1)	0.935	0.935
F000	656.0	656.0
F000'	654.97	
h,k,lmax	12,14,22	12,14,22
Nref	8261	7840
Tmin,Tmax	0.721,0.911	0.632,0.746
Tmin'	0.620	

```
Correction method= # Reported T Limits: Tmin=0.632 Tmax=0.746
AbsCorr = MULTI-SCAN

Data completeness= 0.949          Theta(max)= 30.457

R(reflections)= 0.0387( 5634)          wR2(reflections)= 0.0912( 7840)

S = 1.035                Npar= 361
```

The following ALERTS were generated. Each ALERT has the format
test-name_ALERT_alert-type_alert-level.
Click on the hyperlinks for more details of the test.

Alert level C

```
PLAT220_ALERT_2_C NonSolvent   Resd 1  C   Ueq(max)/Ueq(min) Range        4.0 Ratio
PLAT222_ALERT_3_C NonSolvent Resd 1  H   Uiso(max)/Uiso(min) Range        4.4 Ratio
PLAT242_ALERT_2_C Low    'MainMol' Ueq as Compared to Neighbors of        Pd1 Check
PLAT906_ALERT_3_C Large K Value in the Analysis of Variance ......      2.190 Check
PLAT911_ALERT_3_C Missing FCF Refl Between Thmin & STh/L=    0.600         14 Report
```

Alert level G

```
PLAT003_ALERT_2_G Number of Uiso or Uij Restrained non-H Atoms ...          9 Report
PLAT154_ALERT_1_G The s.u.'s on the Cell Angles are Equal ..(Note)      0.014 Degree
PLAT187_ALERT_4_G The CIF-Embedded .res File Contains RIGU Records          2 Report
PLAT301_ALERT_3_G Main Residue  Disorder .............(Resd  1  )         11% Note
PLAT720_ALERT_4_G Number of Unusual/Non-Standard Labels ..........          8 Note
PLAT793_ALERT_4_G Model has Chirality at P1          (Centro SPGR)          R Verify
PLAT860_ALERT_3_G Number of Least-Squares Restraints ............          48 Note
PLAT910_ALERT_3_G Missing # of FCF Reflection(s) Below Theta(Min).          3 Note
PLAT912_ALERT_4_G Missing # of FCF Reflections Above STh/L=  0.600        404 Note
PLAT913_ALERT_3_G Missing # of Very Strong Reflections in FCF ....          3 Note
PLAT941_ALERT_3_G Average HKL Measurement Multiplicity ..........         4.7 Low
PLAT978_ALERT_2_G Number C-C Bonds with Positive Residual Density.          3 Info
```

0 **ALERT level A** = Most likely a serious problem - resolve or explain
0 **ALERT level B** = A potentially serious problem, consider carefully
5 **ALERT level C** = Check. Ensure it is not caused by an omission or oversight
12 **ALERT level G** = General information/check it is not something unexpected

1 ALERT type 1 CIF construction/syntax error, inconsistent or missing data
4 ALERT type 2 Indicator that the structure model may be wrong or deficient
8 ALERT type 3 Indicator that the structure quality may be low
4 ALERT type 4 Improvement, methodology, query or suggestion
0 ALERT type 5 Informative message, check

It is advisable to attempt to resolve as many as possible of the alerts in all categories. Often the minor alerts point to easily fixed oversights, errors and omissions in your CIF or refinement strategy, so attention to these fine details can be worthwhile. In order to resolve some of the more serious problems it may be necessary to carry out additional measurements or structure refinements. However, the purpose of your study may justify the reported deviations and the more serious of these should normally be commented upon in the discussion or experimental section of a paper or in the "special_details" fields of the CIF. checkCIF was carefully designed to identify outliers and unusual parameters, but every test has its limitations and alerts that are not important in a particular case may appear. Conversely, the absence of alerts does not guarantee there are no aspects of the results needing attention. It is up to the individual to critically assess their own results and, if necessary, seek expert advice.

Publication of your CIF in IUCr journals

A basic structural check has been run on your CIF. These basic checks will be run on all CIFs submitted for publication in IUCr journals (*Acta Crystallographica*, *Journal of Applied Crystallography*, *Journal of Synchrotron Radiation*); however, if you intend to submit to *Acta Crystallographica Section C* or *E* or *IUCrData*, you should make sure that full publication checks are run on the final version of your CIF prior to submission.

Publication of your CIF in other journals

Please refer to the *Notes for Authors* of the relevant journal for any special instructions relating to CIF submission.

PLATON version of 18/05/2022; check.def file version of 17/05/2022

Datablock mo_22jun06wtd11_0m - ellipsoid plot

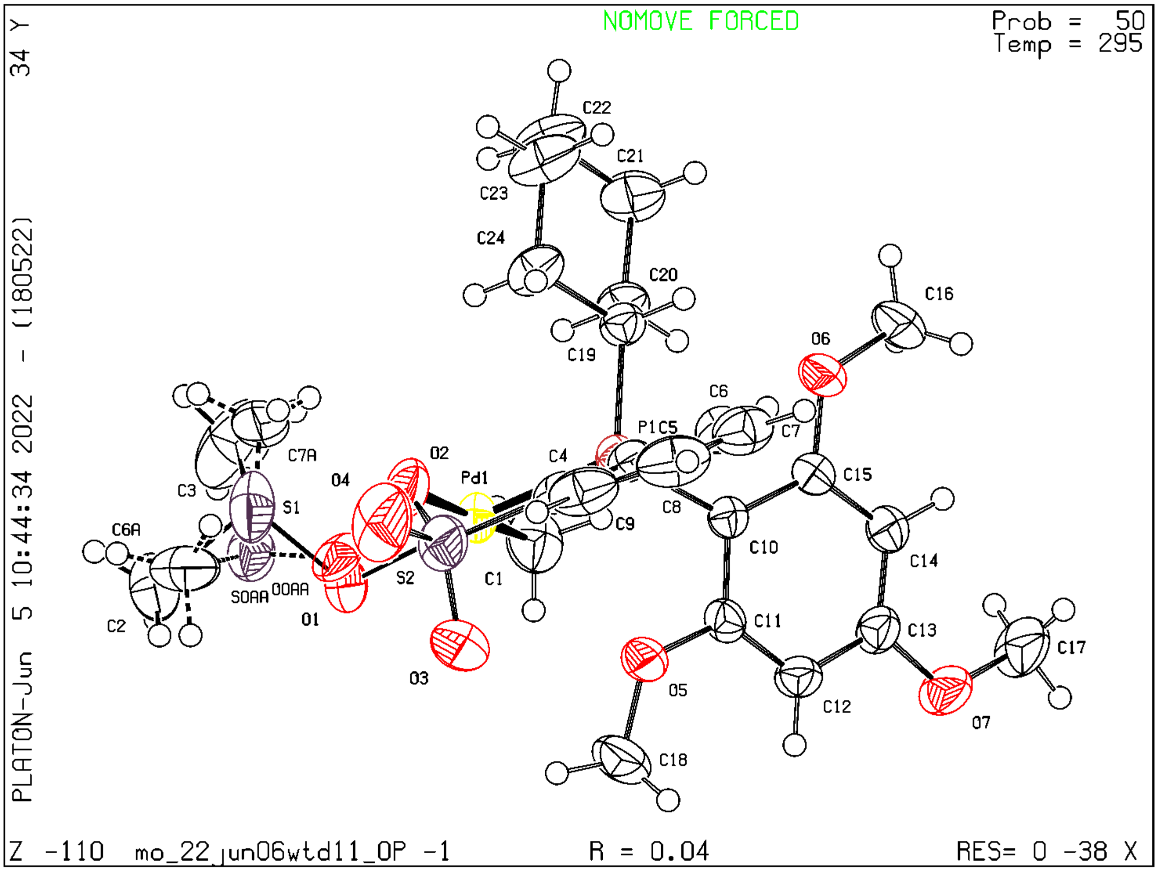

Supplement: nwad039_Supplemental_Files [file nwad039_supplemental_files.zip › checkcif_Pd4.pdf]
